# Effectiveness of Personal Protective Equipment for Healthcare Workers Caring for Patients with Filovirus Disease: A Rapid Review

**DOI:** 10.1371/journal.pone.0140290

**Published:** 2015-10-09

**Authors:** Mona Hersi, Adrienne Stevens, Pauline Quach, Candyce Hamel, Kednapa Thavorn, Chantelle Garritty, Becky Skidmore, Constanza Vallenas, Susan L. Norris, Matthias Egger, Sergey Eremin, Mauricio Ferri, Nahoko Shindo, David Moher

**Affiliations:** 1 Clinical Epidemiology Program, Centre for Practice-Changing Research, Ottawa Hospital Research Institute, Ottawa, Ontario, Canada; 2 TRIBE graduate program, University of Split School of Medicine, Split, Croatia; 3 School of Epidemiology, Public Health and Preventive Medicine, Faculty of Medicine, University of Ottawa, Ottawa, Ontario, Canada; 4 Independent Consultant, Ottawa, Ontario, Canada; 5 World Health Organization, Geneva, Switzerland; 6 Institute of Social and Preventive Medicine, University of Bern, Bern, Switzerland; 7 Centre for Infectious Disease Epidemiology and Research, School of Public Health and Family Medicine, Faculty of Health Sciences, University of Cape Town, Cape Town, South Africa; 8 Department of Community Health Sciences, Faculty of Medicine, University of Calgary, Calgary, Alberta, Canada; Division of Clinical Research, UNITED STATES

## Abstract

**Background:**

A rapid review, guided by a protocol, was conducted to inform development of the World Health Organization’s guideline on personal protective equipment in the context of the ongoing (2013–present) Western African filovirus disease outbreak, with a focus on health care workers directly caring for patients with Ebola or Marburg virus diseases.

**Methods:**

Electronic databases and grey literature sources were searched. Eligibility criteria initially included comparative studies on Ebola and Marburg virus diseases reported in English or French, but criteria were expanded to studies on other viral hemorrhagic fevers and non-comparative designs due to the paucity of studies. After title and abstract screening (two people to exclude), full-text reports of potentially relevant articles were assessed in duplicate. Fifty-seven percent of extraction information was verified. The Grading of Recommendations Assessment, Development and Evaluation framework was used to inform the quality of evidence assessments.

**Results:**

Thirty non-comparative studies (8 related to Ebola virus disease) were located, and 27 provided data on viral transmission. Reporting of personal protective equipment components and infection prevention and control protocols was generally poor.

**Conclusions:**

Insufficient evidence exists to draw conclusions regarding the comparative effectiveness of various types of personal protective equipment. Additional research is urgently needed to determine optimal PPE for health care workers caring for patients with filovirus.

## Introduction

The family *Filoviridae* includes three genera, *Cuevavirus*, *Ebolavirus*, and *Marburgvirus*. Four ebolaviruses (Bundibugyo virus, Ebola virus, Sudan virus, and Taï Forest virus) cause Ebola virus disease (EVD) and two maburgviruses (Marburg virus and Ravn virus) cause Marburg virus disease (MVD) [1]. EVD and MVD are severe illnesses in humans, with a combined mean case fatality rate of 55.4% [2]. The natural hosts of the filoviruses are unknown but fruit bats have been implicated in the transmission of Marburg virus and Ravn virus [3,4]. The majority of cases in an outbreak become infected from direct contact through non-intact skin or mucous membranes with the bodily fluids of infected symptomatic persons or the body of deceased persons. Airborne transmission of filoviruses has not been documented in humans [5,6]. The incubation period for both filovirus diseases is in the range of 2 to 21 days [7,8]. Patients are infectious once they start to exhibit symptoms which include sudden onset fever, fatigue, headaches and muscle pain followed by diarrhea, vomiting, and lethargy [7–9]. EVD patients frequently experience severe dehydration, kidney and liver dysfunction.[7] Patients of both diseases may experience internal and external bleeding in the later course of the disease, around 5–7 days [7,8,10]. Although different diseases, EVD and Marburg virus disease (MVD) have similar presentations, case fatality rate, and transmission mechanism. Both have no specific treatment to date.

Outbreaks of EVD have occurred since 1976. A rapidly evolving outbreak that has presumed to have emerged in December 2013[11], and is ongoing as of the date of publication of this manuscript, has yielded the highest number of cases and deaths. As of 30 August 2015, 11290 deaths have occurred from among 28073 confirmed, probable, and suspected cases reported from countries most affected by the outbreak, namely, Guinea, Liberia, and Sierra Leone [12]. When infection prevention and control (IPC) measures are inadequate, there is a high risk of transmission to healthcare workers (HCWs) treating those with suspected or known filovirus infection. In the three most affected countries, a total of 513 reported deaths have occurred as of 30 August 2015 among 881 HCWs known to have been infected with EVD [12]. Inadequate source control, insufficient training on appropriate IPC practices, shortages of personal protective equipment (PPE) and improper PPE use, long working hours in the face of a shortage of medical personnel, and transmission outside the patient care setting are possible explanations [12,13].

Specifications for the components of PPE to be worn during delivery of care are important, not only to reduce the likelihood of transmission to HCWs from a barrier standpoint but to also ensure comfort and safety as wearing PPE increases the risk of heat stress and the loss of dexterity [14,15]. The PPE worn should also allow for the best possible care to patients. Currently, PPE recommendations and protocols differ across organizations responding to the outbreak, including the World Health Organization (WHO) and Médecins Sans Frontières (MSF) [16,17].

WHO issued recommendations on PPE for use by HCWs managing patients with known or suspected filovirus disease in October 2014 [17]. To inform these recommendations, we performed a rapid review of the evidence on the effectiveness of different types of PPE in preventing ebolavirus transmission and on levels of dexterity and discomfort, for example, comparing gowns versus coveralls or comparing single versus doubles gloves.

## Methods

We performed a “rapid review”, a type of review produced using accelerated and/or modified systematic review methods in order to accommodate an expedited turnaround time [18]. The rapid review was conducted over a 7-week period from 28 July to 12 September 2014. This rapid review was guided by a protocol (S1 Appendix) that was developed *a priori* by the authors and then reviewed by the guideline development group–a group of external experts who were invited by WHO to formulate recommendations regarding PPE use. The protocol allowed for modifications of scope and analysis during review conduct once the nature and volume of the evidence was known. We used the PRISMA reporting guideline for systematic reviews for the reporting of our work in this paper (S2 Appendix) [19].

The research question for this review was: what are the benefits and harms of double gloves, full face protection, head cover, impermeable gowns, particulate respirators, and rubber boots as PPE when compared with alternative and potentially less robust PPE for HCWs directly caring for patients with filovirus disease? Our lens for the review starts with the prevention of transmission to the HCW and subsequent transmission prevention from HCW to other patients.

### Eligibility criteria for studies

We included studies of HCWs in health care facilities providing direct patient care to persons who had known or suspected filovirus disease (EVD or MVD) caused by any ebolavirus or marburgvirus. Health care facilities refers to both treatment centers specifically set up for managing filovirus disease (Ebola Treatment Centers), as well as to general health care treatment facilities such as health centers and hospitals.

**Fig 1:**
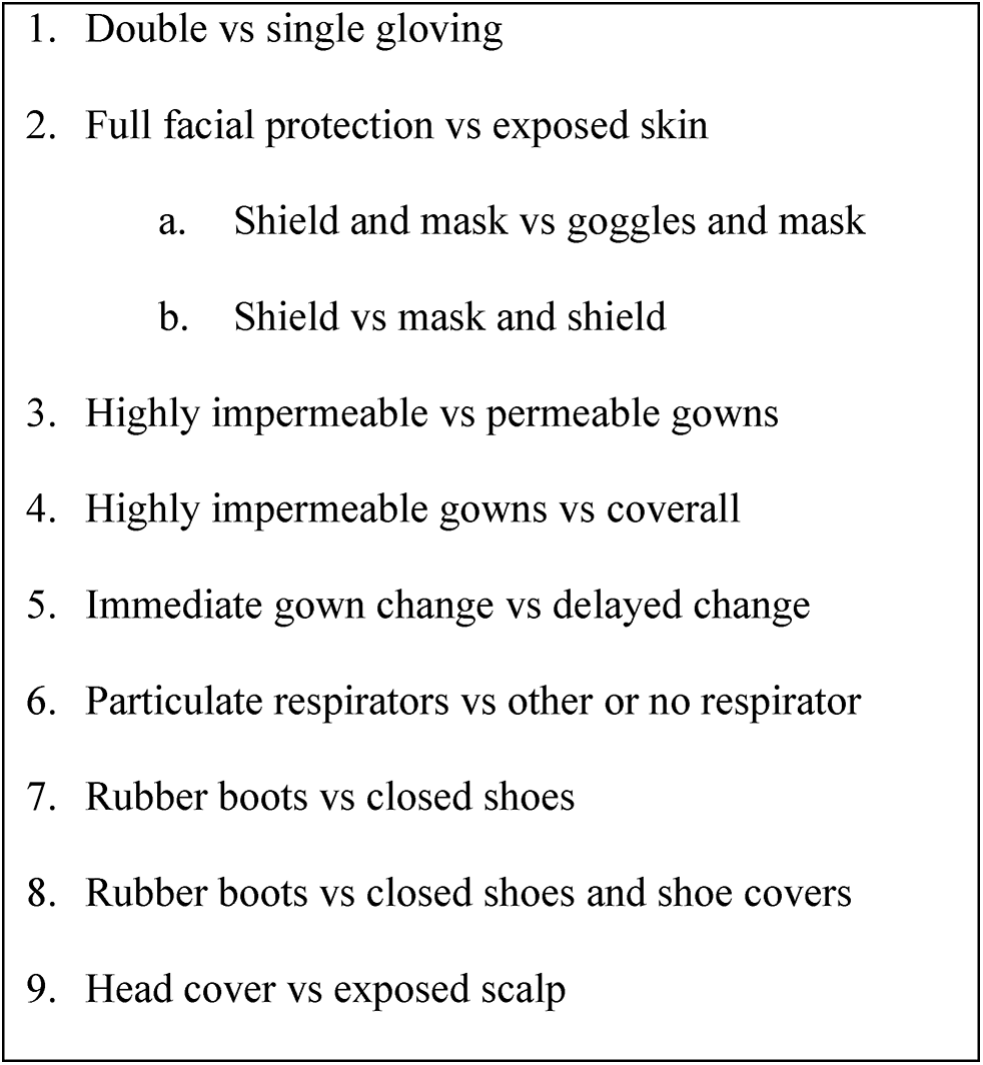
Comparisons of personal protective equipment to prevent transmission of ebolavirus to health care workers.

We defined a list of PPE components and comparisons as a guide to identify relevant studies, but remained open to other comparisons if encountered in the literature (Fig 1).

Outcomes were specified by the guideline development group and included transmission of ebolavirus to HCWs and from HCWs to patients and adverse effects of using PPE such as perceived inconvenience or discomfort, injuries (e.g. needlestick injury), dexterity, reduced visibility, and heat-related events. Other outcomes in reports were also extracted.

As per our protocol, we first sought high quality systematic reviews, evidence-based clinical practice guidelines, and health technology assessments. In their absence, primary studies were retrieved using an evidence hierarchy: randomized controlled trials; quasi-experimental designs; comparative cohort, case-control studies, and cross-sectional studies, and in the event of no comparative evidence, we searched for and included data from non-comparative studies.

We considered studies published in either English or French published in 1967 (when filovirus disease first emerged) or later. No geographical restrictions were applied.

Because our initial search identified few publications, we expanded our search (as per our protocol) to include studies reporting on Crimean-Congo hemorrhagic fever or Lassa fever as they were considered to have a similar mode of human-to-human transmission and infectivity to the filovirus diseases.

### Literature search

Electronic search strategies were developed and tested iteratively by an experienced medical information specialist. Between 28 July and 7 August 2014, we searched Ovid MEDLINE and Ovid MEDLINE In-Process & Other Non-Indexed Citations, The Cochrane Database of Systematic Reviews (limited to the Cochrane Infectious Diseases Review Group reviews and specialized register), EMBASE, and African Index Medicus. Search strategies were not limited by language or year. A combination of controlled vocabulary and text-word terms were used (S3 Appendix), where possible. The initial MEDLINE search strategy was adapted to the other databases. Study design filters were applied.

Grey literature sources were searched on 20–22 August 2014 using the ProQuest Dissertation and Theses Databases and the Google Search Engine. We also searched ClinicalTrials.gov and WHO International Clinical Trials Registry Platform search portal to seek ongoing and completed trials. References of included studies were scanned. Acquisition of articles was focused to those available electronically through the research team’s institutional subscription; some full-text reports were sought elsewhere where time permitted.

### Study selection and data extraction

De-duplicated citations in Reference Manager were uploaded to Distiller Systematic Review software for screening. Single reviewers assessed titles and abstracts with excluded records verified by a second reviewer. Any records with disagreements underwent full-text screening. Full-text reports were reviewed independently by two people, and disagreements between pairwise reviewers were resolved by consensus or a third reviewer. Screening forms were pilot-tested using 15 (title/abstract) and 10 records (full text), respectively.

Single extractors collected information from studies, and a second person verified 57% of information. The extraction form was pilot-tested on nine included studies. Authors of included studies were not contacted for additional information due to time constraints.

### Evidence Synthesis

Study characteristics are described narratively. Due to the nature and heterogeneity of included studies, meta-analysis of results was not done. Plots summarizing the proportion of HCWs reported to have experienced an outcome were produced where appropriate. The denominator included HCWs at risk for whom we knew the PPE worn.

Risk of bias assessments were not done due to the lack of validated instruments to assess the methodological quality of non-comparative designs [20].

Domains of the Grading of Recommendations Assessment Development and Evaluation (GRADE) framework were used to inform judgments on the quality of the evidence across studies for each outcome [21]. This framework initially considers evidence from observational studies as low quality and randomized controlled trials as high quality. Five domains related to quality are then assessed and used to determine the quality of the body of evidence for each outcome across studies: study limitations, consistency, directness, precision, and publication bias. Observational evidence without important threats to validity can be upgraded when there is a dose-response effect, a large magnitude of effect, or because plausible biases may have decreased the observed effect.

The study limitations domain addresses the risk of bias (internal validity) of studies [21]. Consistency addresses the degree to which studies yield similar results,while directness considers the degree to which the evidence aligns with the population, interventions, and outcomes of interest[22,23]. Precision judges the extent of random error by taking the sample size, number of observed events, and confidence intervals into consideration [24]. The publication bias domain addresses the degree to which published and unpublished studies yield systematically different findings [25].

### Protocol modifications

We were able to increase the verification of extracted information from 10% to 57% of included studies.

## Results

### Identification of relevant studies

**Fig 2:**
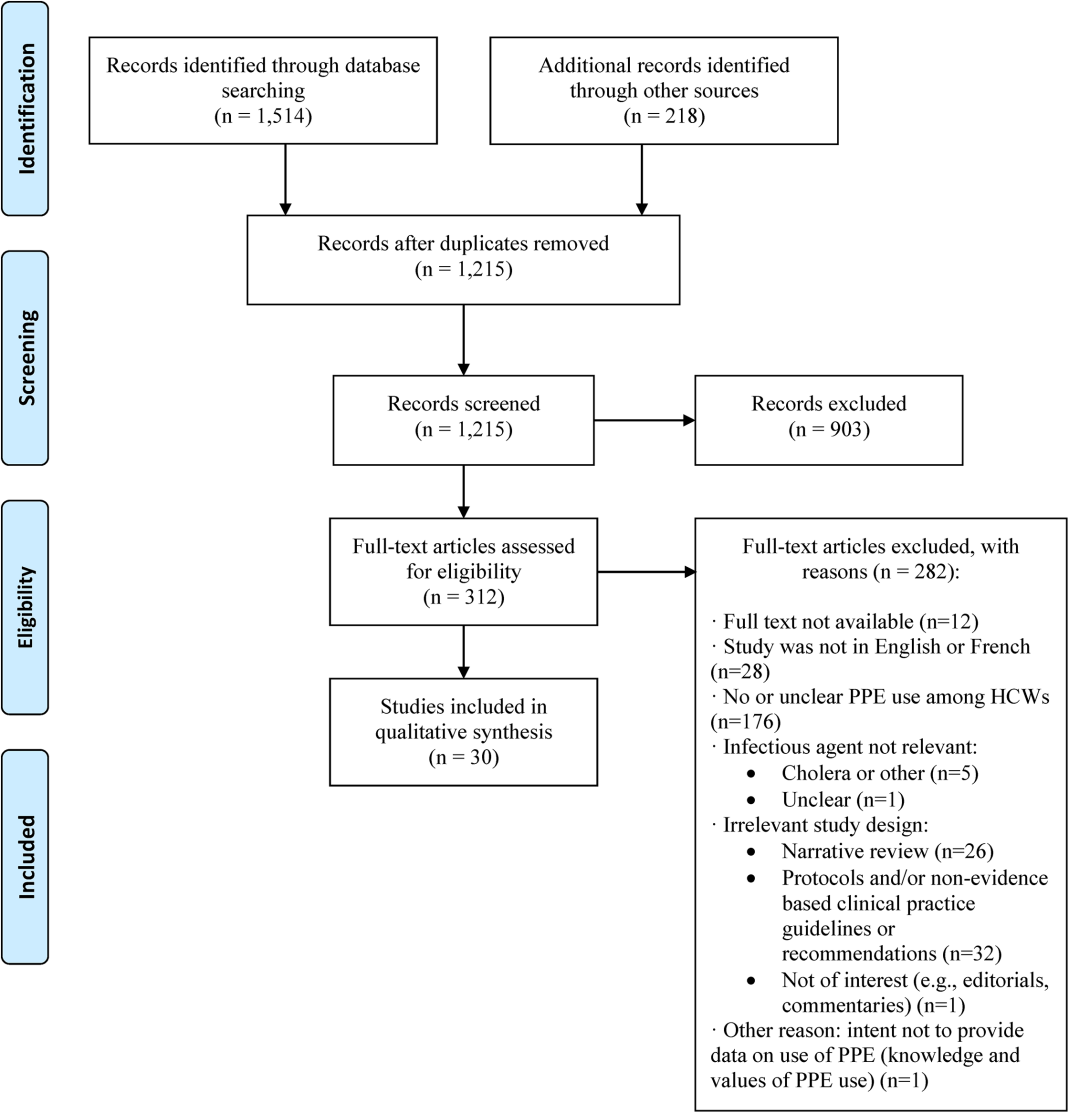
PRISMA (Preferred Reporting Items for Systematic Reviews and Meta-Analyses) flow diagram of the study selection process.

A total of 1,215 unique records were retrieved. No systematic reviews, evidence-based clinical practive guidelines or health technology assessment reports were identified. Furthermore, no comparative primary studies or ongoing trials were identified. However, 30 non-comparative studies [26–55] fulfilled the eligibility criteria (Fig 2). Ten of the 30 studies were identified through a scan of reference lists of included studies. A list of studies excluded following full-text review and reasons for exclusion are provided in S4 Appendix.

### Characteristics of studies and study populations

**Fig 3:**
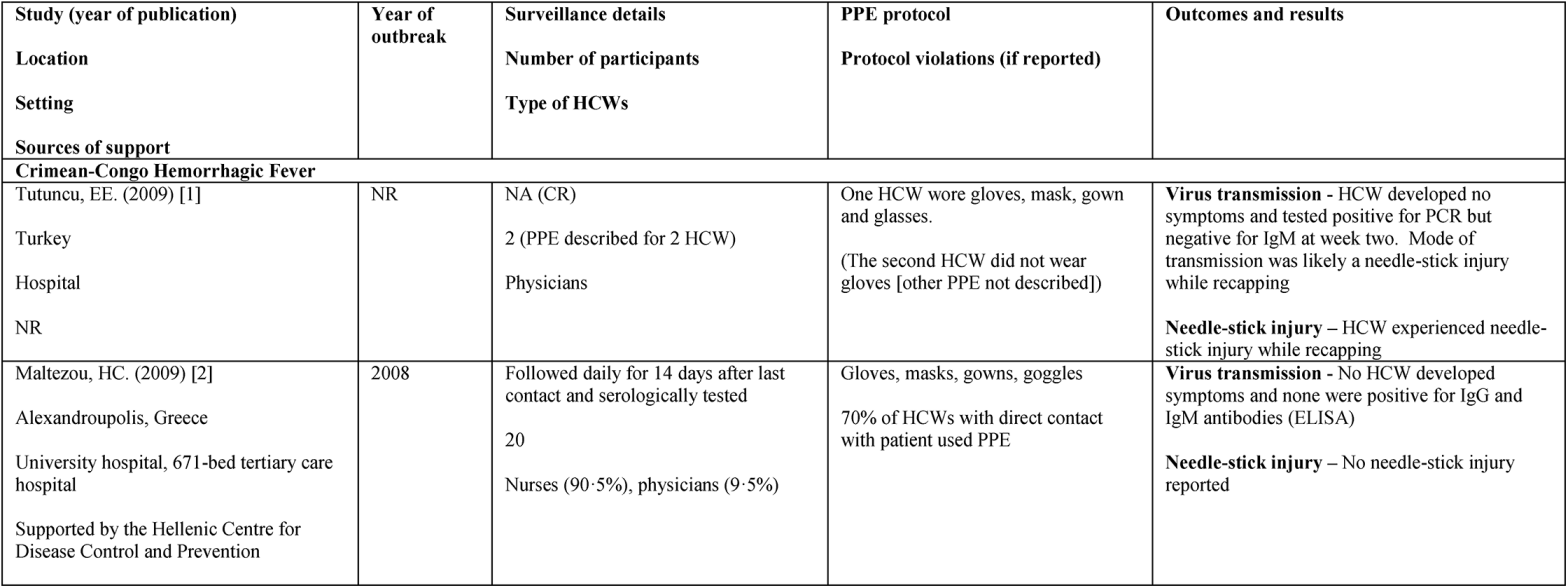
Non-comparative studies of healthcare workers wearing gloves, masks, gowns, and glasses/goggles. Abbreviations: ELISA = enzyme-linked immunosorbent assay; HCW = healthcare worker; IgG = immunoglobulin G; IgM = immunoglobulin M; NA = not applicable; NR = not reported; PPE = personal protective equipment.

The characteristics of studies reporting on gloves are provided in Fig 3. Studies reporting on other PPE combinations are summarized in S1–S16 Tables. Studies were published between 1969 and 2013 and conducted in Africa [26,27,32,33,36,38,39,49–53,55], Europe (including Turkey) [30,31,34,35,37,42,43,47,48,54], South Asia and Western Asia [28,29,46], North America [40,41,45], and one study included HCWs in Africa and HCWs in Europe because of a patient repatriated to Europe [44].

Eleven studies [26,27,32,33,35,36,39,49,50,54,55] reported on filoviruses, two on unspecified viral hemorrhagic fevers (VHF) [37,45], 11 on Crimean-Congo hemorrhagic fever [28–31,34,42–44,46,48,53], and six on Lassa fever [38,40,41,47,51,52]. Of the eight studies reporting on ebolavirus, three reported outbreaks of Sudan virus [32,33,55], four of Ebola virus [26,27,36,49], and one of Taï Forest virus [35].

While three studies were case reports of HCWs [34,46,54], a majority of studies involved contact tracing of HCWs providing care to index patients. Seven studies monitored HCWs for at least three weeks for outcomes, while others used a shorter follow-up,did not report this information, or did not actively follow participants.

Most studies examined nurses and physicians with or without other personnel providing patient care, including medical students, assistants, and other auxiliary staff members. Data from some studies included other personnel not providing direct patient care (e.g., laboratory workers, housekeeping staff, and administrative staff). Sample sizes were not consistently reported, and some studies reported the total number of contacts but did not specify the proportion of HCWs.

### Personal Protective Equipment

Only one study was designed with the intent to evaluate PPE use [30]. The PPE protocols varied across and within studies, i.e., over the duration of the care period or among HCWs. Several reports [27,36–39,41,49,50,55] described changes to the protocol, including delayed implementation of PPE or sequential introduction of PPE components during an outbreak. Three reports [29,38,44] traced HCW contacts from multiple health care facilities and described varying PPE protocols across the settings. A few studies reported varied adherence to the PPE protocol among HCWs within a given study [30,31,43] or only described the PPE used by a subset of HCW contacts (e.g., those who subsequently developed the disease) [27,28,34,46]. Three studies [33,42,50] reported adoption of established PPE guidelines for the management of patients with VHFs including those developed by WHO and the Advisory Committee on Dangerous Pathogens.

Although we did not perform a formal assessment of the completeness of reporting across studies, our impression is that the reporting of PPE protocols was poor. In most reports, only a general description was provided of the components of PPE used without indication of the quality or specific characteristics (e.g., disposability, permeability, and other specifications). Further, important details including the quantity of each component used simultaneously by a single HCW (i.e., single or double gloves or gowns) was not reported. Some studies only partially reported the PPE protocol. For example, several studies specified one element of PPE (e.g., gloves, respirators, masks) but the remaining components were not described in detail (e.g., ‘protective clothing’, ‘barrier techniques’) [26,29,41,47,54].

### Outcomes

Nearly all studies (90%; 27/30) reported on virus transmission from infected patients to HCWs. One study [45] reported no outcomes of interest as VHF was ruled out. Half of the studies measured virus transmission based on symptoms, serology and/or polymerase chain reaction (PCR) for at least a subset of HCWs [28–30,33,34,36,38–40,43,46,47,49–51,53,55]. The remaining studies used only symptoms [27,41,44,48,54], serologic or PCR/reverse transcription-PCR testing [31,35,52], or the method of ascertainment was not reported [26,32,37,42]. Three studies [30,31,52] reported on antibody prevalence among HCWs exposed to Crimean-Congo and Lassa fever virus.

The proportion of HCWs who experienced an event are presented (Figs 3–6, S1–S16 Tables), grouped by the combination of PPE elements worn. For filovirus disease, five of 11 studies reported virus transmission to HCWs having worn a variety of PPE combinations (Fig 4). One of those studies was unclear regarding timing of transmission (i.e., at what point during PPE protocol). Eight of 16 studies examining other types of VHFs reported viral transmission to HCWs having worn a various PPE combinations.

**Fig 4:**
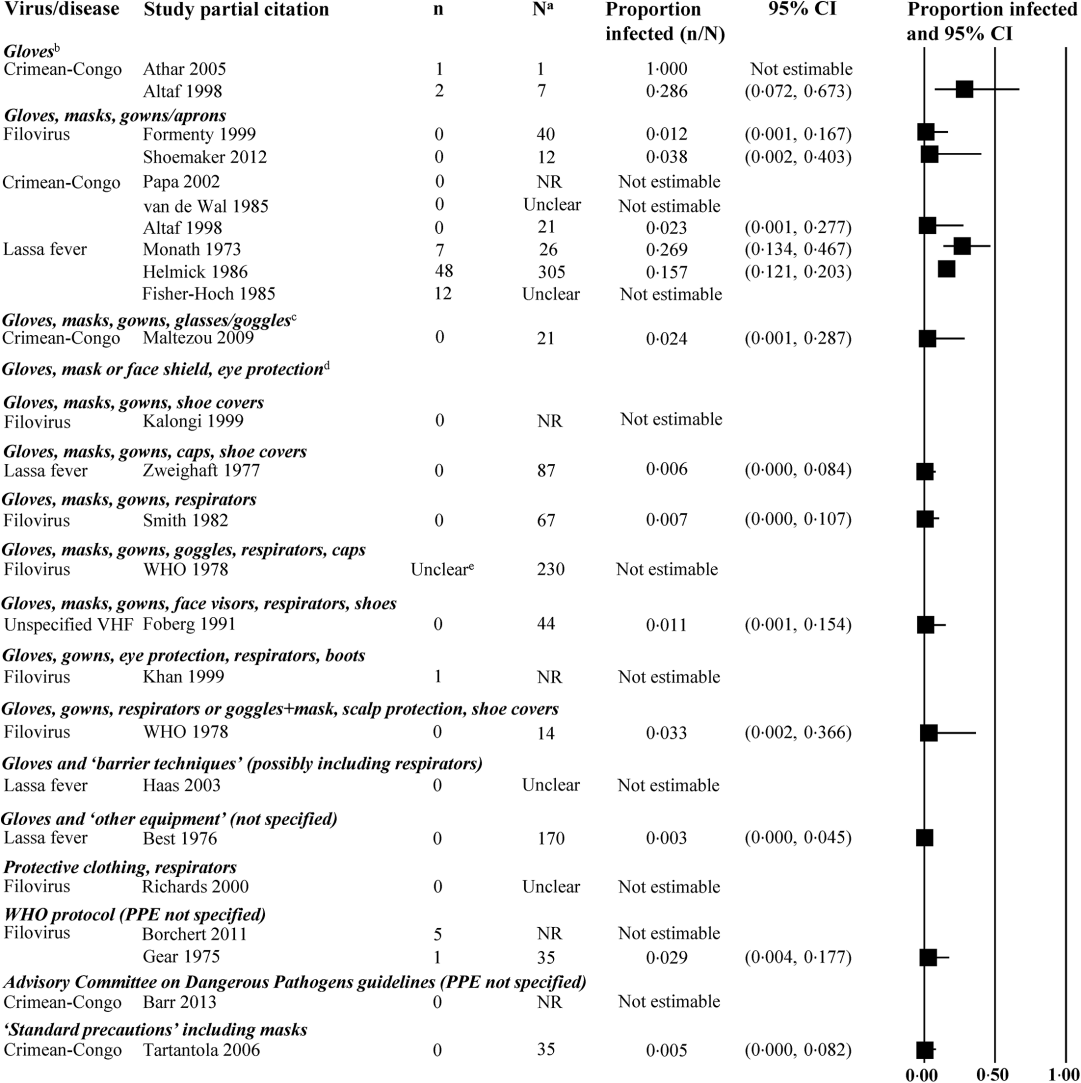
Virus transmission in non-comparative studies of healthcare workers wearing personal protective equipment. Abbreviations: CI = confidence interval; n = number of events; N = number of HCWs at risk for whom we knew the PPE worn; NR = not reported; WHO = World Health Organization. ^a^Most studies did not provide data on all healthcare workers; only workers with available data were included. ^b^Case reports: One report on filovirus (Martini 1969) and one on Crimean-Congo hemorrhagic fever (Naderi 2011) were identified. ^c^One case report on Crimean-Congo hemorrhagic fever (Tutuncu 2009) was identified. ^d^One case report on Crimean-Congo hemorrhagic fever (Naderi 2011) was identified. ^e^PPE protocol was altered during process of care; unclear whether events occurred before or after the enhanced PPE protocol was implemented.

**Fig 5:**
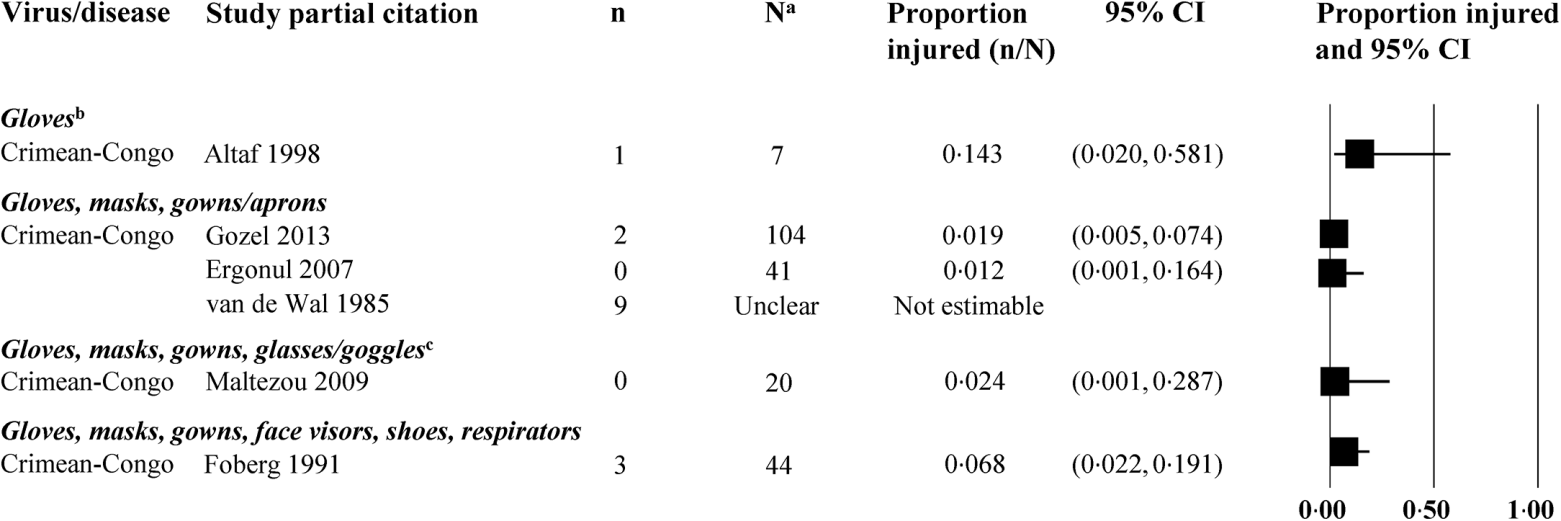
Needle stick injury in non-comparative studies of healthcare workers wearing personal protective equipment. Abbreviations: CI = confidence interval; n = number of events; N = number of HCWs at risk for whom we knew the PPE worn. ^a^Most studies did not provide data on all healthcare workers; only workers with available data were included. ^b^One case report on filovirus (Martini 1969) was also identified. ^c^One case report on Crimean-Congo hemorrhagic fever (Tutuncu 2009) was also identified.

**Fig 6:**
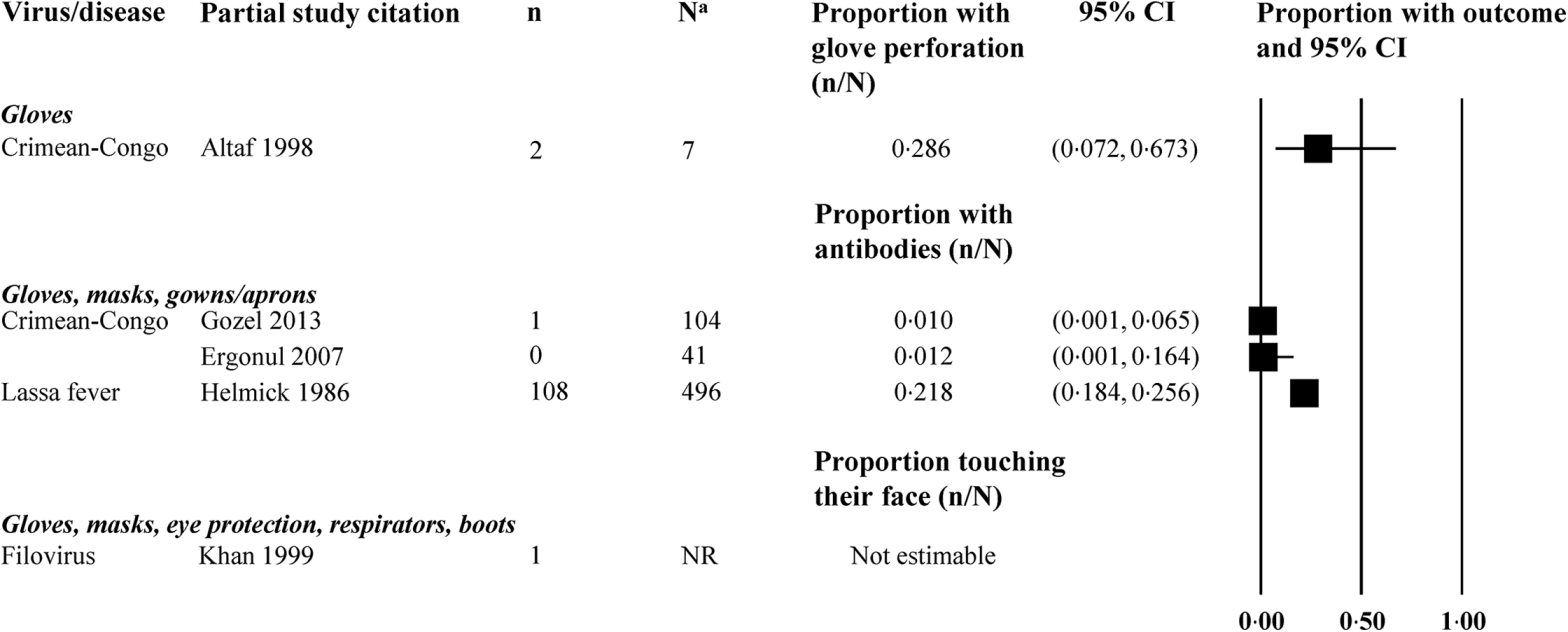
Other adverse events in non-comparative studies of healthcare workers wearing personal protective equipment. Abbreviations: CI = confidence interval; n = number of events; N = number of HCWs at risk for whom we knew the PPE worn; NR = not reported; WHO = World Health Organization. ^a^Most studies did not provide data on all healthcare workers; only workers with available data were included.

No studies reported on dexterity with the use of gloves or on adverse effects such as discomfort, reduced visibility, high temperatures, or humidity. Eight studies reported on needle stick injuries [29–31,34,37,43,53,54], one study on inadvertent touching of face with contaminated gloves [27], and one on glove perforation [29]. The proportion of HCWs with other outcomes (needle stick injury, glove perforation, and antibody prevalence) are shown in Figs 5 and 6.

### Sources of Support

One study clearly indicated their sources of financial support [47]. Four studies indicated sources of support but did not provide the nature of the support [30,32,39,43]. Four studies listed the participation of organizations in providing or inferring outbreak support [27,33,49,55]. No companies manufacturing PPE components were listed among the involved organizations.

## Discussion

In this rapid review, we identified 30 observational studies of PPE in the context of VHFs, of which 11 addressed filovirus disease. However, none of these studies compared different approaches to personal protection or different types of PPE. All studies included in this review, therefore, provide insufficient evidence on the comparative effectiveness of the different PPE protocols.

Only one study was designed with the intent to evaluate PPE use [30]. Most reports involved contact tracing of HCWs providing care to index patients.

Using the GRADE framework, the quality of the body of evidence for all outcomes was assessed as very low. Despite the lack of validated instruments for evaluating the internal validity of non-comparative studies, we can assume the literature to be at a high risk of bias. Given the body of evidence is non-comparative, this poses very serious limitations in terms of the directness of the evidence to the question of comparative effectiveness. The heterogeneity of the PPE components across and within studies over the duration of care and the heterogeneity of study designs limit the ability to assess the consistency of the data; most PPE combinations were reported by one study only. Estimates of the proportion of HCWs contracting the infection was generally based on small numbers and therefore imprecise. Publication bias could not be assessed quantitatively, and we do not know whether unpublished studies exist with systematically different findings.

Only some of the studies monitoring a cohort of HCWs over a period of time did so for at least three weeks, the maximum incubation period described in the literature for filoviruses. Some cases of transmission may, therefore, have been missed. WHO recommends reverse transcriptase polymerase chain reaction (RT-PCR) or enzyme linked immunosorbent assay (ELISA) tests for the diagnosis of EVD, and we know at least some of the included studies incorporated these methods in the diagnostic work-up. However, a positive PCR test indicates the presence of viral particles which may or may not be infectious; therefore cell culture is needed for definitive diagnosis for EVD and MVD. Some of the studies relied solely on self-reported symptoms and temperature readings. Such studies are susceptible to bias as data on PPE was self-reported and, in a number of cases, retrospectively ascertained. In addition, it was unclear for many of the studies whether all HCWs in contact with patients were followed. It is possible that the proportion of HCWs infected were overestimated because those contracting EVD were probably more likely to be identified than those not contracting the disease.

When the PPE protocol was described, the reporting was poor and often lacked important details on the characteristics of the equipment (e.g., quality, disposability, permeability) and methods of donning and doffing. In some studies, outcomes could not be attributed to a particular PPE protocol because the PPE components were either not reported or poorly reported, or the protocol was altered during the process of care. The PPE worn by all exposed HCWs was not always adequately described. In some studies the recommended PPE was reported, while adherence was not. Lastly, determining the proportion of HCWs infected was often precluded by inadequate reporting of the sample size of exposed HCWs.

Two EVD studies postulated that transmission was due to protocol violations[33], including possible inadvertent touching of the face [27]. Where transmission was observed, it was difficult to attribute causation because of limitations of the observational studies included in this review, the possibility of other sources of transmission (for example, EVD cases in the community), and poor reporting. This was true even where needle stick injuries or glove perforations occurred as necessary details to definitively attribute causation (e.g., details and timing of PPE use) were not provided. Phylogenetic analyses can contribute to estimating the source of infection but such analyses were not done in the studies of HCWs included in this review.

Finally, it is important to consider that PPE is only one factor within the larger context of IPC. Other factors such as hand hygiene and environmental cleaning were beyond the scope of this review, but are critical elements in the development and implementation of IPC.

### Strengths and limitations of the rapid review

This rapid review was guided by protocol developed *a priori*. Although we limited the extent of bibliographic database searching, we searched African Index Medicus and grey literature sources to reduce the risk of location and publication biases. We used standard systematic approaches for study selection, data extraction, and synthesis. We also assessed the quality of the body of evidence using GRADE. Although intended as a rapid review, our work closely approximated that of a systematic review.

Limitations of our work stem from time constraints: search strategies were not peer-reviewed, we were unable to locate twelve full-text articles (S4 Appendix); and outcome extractions for 40% of studies were not verified by a second person.

### Future research

Circumstances surrounding the ongoing EVD outbreak, including extremely challenging working conditions, scarce resourses, population mobility, and deteriorated healthcare systems and infrastructures, make it difficult to collect data for inclusion in this review and possibly for any future review update. However, efforts to collect data should be undertaken wherever possible. For example, comparative observational studies are needed to supplement the current evidence. Although case-control studies may be the most feasible, comparative cohort studies would be scientifically stronger. Further, randomized trials of the various components of PPE may be difficult to implement. More specifically, studies evaluating the current PPE protocols of organizations should be undertaken. For example, the WHO guidance released in on 31 October 2014 recommends the use of face shields or googles for eye protection; however, empirical data comparing those forms of eye protection regarding transmissions and adverse effects (e.g., fogging, visibility) are currently not available.

Future research would benefit from standardized data collection instruments and population-based registries enabling synthesis of larger sample sizes. Also, an ongoing environmental scan of in-progress studies from various healthcare organizations providing service to African nations should be conducted. Although outside the scope of the rapid review, evidence is also needed for workers not providing direct clinical care.

Phylogenetic tracing should be performed where possible to estimate source of infection. Attention should also be given to collecting adverse outcomes of PPE use (e.g., inconvenience, discomfort, heat-related events, impaired dexterity, etc.).

Future studies need to be carefully and completely reported, using reporting guidelines such as CARE for clinical case reports, STROBE for observational studies, and CONSORT for randomized controlled trials. The use of such guidelines will facilitate adequate reporting and thus increase the usability of research reports, which will, in turn, facilitate decision making.

Another aspect of future research involves materials science and engineering. Technological advances to improve generally understood safety issues with PPE use, such as dexterity, comfort, and heat, and also to minimize the risk of contamination during the donning and doffing process while acting as a barrier to virus transmission during use would be ideal.

### Implications for policy and practice

While we await better evidence to inform this topic, organizations and individuals need to consider how best to move forward with recommendations regarding PPE use. Although some may consider a zero-tolerance (100% effectiveness) approach, a number of reasons may preclude this as a strategy. First, we located no evidence that suggests totally impermeable materials are more effective than ‘only’ fluid-resistant materials for reducing virus transmission. Second, as mentioned earlier, a number of reasons may account for virus transmission, such as long working hours and transmission outside the patient care setting; WHO is in the process of finalizing an epidemiologic analysis of a subset of HCWs to better understand transmission cause. Although the adequacy of the PPE worn is important, correct donning and doffing of the equipment is an integral process for preventing infection [56]. There is general agreement as per knowledge and understanding among co-authors from this recent EVD outbreak that lack of adequate training on IPC, including donning and doffing, was an important factor for virus transmission. Further, a recent literature review identified inappropriate use of PPE and inadequate training as risk factors for HCW infections [57]. As a result, provision of training to HCWs in affected regions was identified as a “key strategy” for preventing ebolavirus transmission. In collaboration with other organizations responding to the ongoing EVD outbreak, WHO has developed job aids for HCWs on how to put on and remove PPE, and provided training on clinical management (including IPC measures) to over 4500 health responders on the ground in Guinea, Liberia, and Sierra Leone as of June 2015 [58,59]. Additional measures, such as ongoing guidance and monitoring of HCWs through the donning and doffing procedure, were also implemented in MSF Ebola treatment centers [56]. Readers can refer to WHO rapid advice guidance as an example of recommendations made by this organization following the completion of this rapid review [17].

## Conclusion

Insufficient comparative evidence exists to draw conclusions regarding the effectiveness and harms of robust personal protective equipment compared with alternative, and potentially less robust personal protective equipment for healthcare workers providing direct patient care to those with filovirus disease.

## Supporting Information

S1 Appendix Rapid review protocol. (PDF)

S2 Appendix Completed PRISMA checklist. (PDF)

S3 Appendix Search strategy. (DOCX)

S4 Appendix List of studies excluded during full-text screening. (DOCX)

S1 Table Study characteristics table of non-comparative studies of healthcare workers wearing gloves. (DOCX)

S2 Table Study characteristics table of non-comparative studies of healthcare workers wearing gloves, gowns/aprons, and masks. (DOCX)

S3 Table Study characteristics of non-comparative studies of healthcare workers wearing gloves, mask or face shield, and eye protection. (DOCX)

S4 Table Study characteristics of non-comparative studies of healthcare workers wearing gloves, masks, gowns, and shoe covers. (DOCX)

S5 Table Study characteristics of non-comparative studies of healthcare workers wearing gloves, masks, gowns, caps, and shoe covers. (DOCX)

S6 Table Study characteristics of non-comparative studies of healthcare workers wearing gloves, masks, gowns, and respirators. (DOCX)

S7 Table Study characteristics of non-comparative studies of healthcare workers wearing gloves, masks, gowns, face visors, respirators, and shoes. (DOCX)

S8 Table Study characteristics of non-comparative studies of healthcare workers wearing gloves, gowns, eye protection, respirators, and boots. (DOCX)

S9 Table Study characteristics of non-comparative studies of healthcare workers wearing gloves, gowns, respirators or goggles plus mask, scalp protection, and shoe covers. (DOCX)

S10 Table Study characteristics of non-comparative studies of healthcare workers wearing gloves, masks, gowns, goggles, respirators, and caps. (DOCX)

S11 Table Study characteristics of non-comparative studies of healthcare workers wearing gloves and barrier techniques (possibly including respirators). (DOCX)

S12 Table Study characteristics of non-comparative studies of healthcare workers wearing gowns and other equipment (not specified). (DOCX)

S13 Table Study characteristics of non-comparative studies of healthcare workers wearing protective clothing and respirators. (DOCX)

S14 Table Study characteristics of non-comparative studies of healthcare workers wearing PPE according to the WHO protocol. (DOCX)

S15 Table Study characteristics of non-comparative studies of healthcare workers wearing PPE according to the Advisory Committee on Dangerous Pathogens guidelines. (DOCX)

S16 Table Study characteristics of non-comparative studies of healthcare workers wearing PPE according to ‘standard precautions’ including masks. (DOCX)
