# Supplementary material for: Effectiveness of Personal Protective Equipment for Healthcare Workers Caring for Patients with Filovirus Disease: A Rapid Review
Source: PLoS One. 2015 Oct 9;10(10):e0140290. doi: 10.1371/journal.pone.0140290 (PMC4599797; doi:10.1371/journal.pone.0140290)
Supplement: S1 Appendix — (PDF) [file pone.0140290.s001.pdf]

Ebola PPE rapid review protocol

July 30, 2014

Overarching question:

What is the evidence of effectiveness (benefits and harms) of double gloves, full face protection, head cover, gowns with high impermeability rating, particulate respirators, and rubber boots as PPE, when compared to alternative less robust PPE, for workers in healthcare facilities caring for patients with filovirus disease?

Rapid review methods

A rapid review will be conducted to meet the needs of the WHO Guideline Development Group to formulate recommendations. Rapid reviews are a type of literature review produced using accelerated and/or modified systematic review methods in order to make concessions to accommodate an expedited turnaround time. This rapid review will be guided by a protocol that includes allowances for modifications regarding scope and analysis during the conduct of the rapid review as decisions are made once the nature and volume of the evidence is known. These decisions will be made in consultation with the WHO Steering Group.

Study inclusion and exclusion criteria for the rapid review

Population

Workers in healthcare facilities refer to individuals who are treating patients who have or are suspected to have contracted filovirus disease.

Health care facilities refer to filovirus outbreak treatment centres or dedicated wards in general hospitals in the context of an outbreak.

If the evidence regarding the context of treating patients with filovirus disease is limited (which is the likely scenario), we will broaden the scope to include other infectious diseases with a similar route of transmission and infectivity. Specific diseases will be determined in consultation with the WHO steering committee.

Interventions*

1. **Double gloves**: Any combination or any type of gloves worn in two layers.

2. **Full face protection**: any kind of equipment or combination that prevents facial skin exposure (e.g., face shield and mask, facial cover, goggles and mask)

3. **Gowns with high impermeability rating:** different types of non-woven water-resistant, long-sleeved gowns. Frequency of gown change as a secondary intervention.

4. **Head covers**: hood or cap covering the entirety of the scalp.

5. **Particulate respirators**: N95 or equivalent mask.

6. **Rubber boots**.

* Interventions maybe referring to usage guidelines. Interventions maybe considered separately or in any combination (bundles) according to each individual study design. Inclusion of studies with different interventions of similar equipment will be decided by the WHO steering committee.

Comparisons*

The following are examples of proposed comparisons that maybe examined:

1. Double gloving versus single gloving.
2. Full facial protection versus exposed skin (e.g., face shield and medical mask versus eye goggles and medical mask, face shield alone versus face shield and medical mask).
3. Highly impermeable gowns versus permeable.
4. Highly impermeable gowns versus coverall.
5. Immediate gown change versus delayed change.
6. Rubber boots versus closed shoes.
7. Rubber boots versus closed shoes and shoe covers.
8. Head cover versus exposed scalp.

*Comparisons maybe considered separately or in any combination (bundles) according to each individual study design. Inclusion of studies with different comparisons of similar equipment will be decided by the WHO steering committee.

Outcomes

The following outcomes are identified as relevant to the planned guideline. Depending on the volume of relevant literature, it may be decided post hoc to limit the review to a subset of outcomes in order to meet the timeline set for this guideline. The finalization and prioritization of the list of outcomes was

made in consultation with the WHO Steering Group and the WHO Guideline Development Group. Outcomes are with respect to health care workers. Patient data will be extracted if feasible.

Double gloves	Full face protection	Impermeable gowns
1. Transmission of the virus to healthcare workers<br>2. Transmission to patients<br>3. Dexterity<br>4. Adverse effects of using gloves (e.g., inconvenience, discomfort)<br>5. Other outcomes**	1. Transmission of the virus to healthcare workers<br>2. Transmission to patients<br>3. Adverse effects of using face protection (e.g., reduced visibility, inadvertent touching of face)<br>4. Other outcomes**	1. Transmission of the virus to healthcare workers<br>2. Transmission to patients<br>3. Adverse effects associated with gown use (e.g., issues relating to temperature)<br>4. Other outcomes**

**An ‘other’ category may be used to capture additional reported outcomes, if feasible to do so. This will allow for identification and further exploration of such outcomes outside the scope of this rapid review. Potential areas to be explored include needlesticks/sharps injuries and glove perforation rates.

Head cover	Particulate respirators	Rubber boots
1. Transmission of the virus to healthcare workers<br>2. Transmission to patients<br>3. Adverse effects of using head covers.<br>4. Other outcomes**	1. Transmission of the virus to healthcare workers<br>2. Adverse effects of using face protection (e.g., reduced visibility, inadvertent touching of face)<br>3. Other outcomes**	1. Transmission of the virus to healthcare workers<br>2. Transmission to patients<br>3. Adverse effects associated with wearing rubber boots.<br>4. Other outcomes**

**An ‘other’ category may be used to capture additional reported outcomes, if feasible to do so. This will allow for identification and further exploration of such outcomes outside the scope of this rapid review. Potential areas to be explored include needlesticks/sharps injuries and glove perforation rates.

Timing

The literature will be limited to reports published or produced from 1967 onwards, when filovirus disease first emerged.

Setting

No geographical restrictions will be applied to the literature.

Study design

The emphasis will first be placed on locating and summarizing evidence from relevant and high quality systematic reviews, meta-analyses, health technology assessments, and/or relevant evidence-based clinical practice guidelines that pertain to filovirus disease. In the absence of high quality systematic reviews, primary studies will be included, using a staged approach for study inclusion and/or analysis. For example, if evidence from comparative studies is sufficient, non-comparative studies will not be included or the analysis of non-comparative studies would be limited. As an iterative process, the methodology for rapid reviews necessitates post-hoc decisions depending on study yield and feasibility.

Depending on the nature and volume of specific study designs that meet our eligibility criteria, it may be decided post-hoc to limit to higher levels of evidence according to study design classifications, and/or to include only those studies that meet a certain level of quality and are lower risk of bias. All such decisions will be made in conjunction with the WHO Steering Group.

Systematic reviews (including health technology assessments and the evidence base of clinical practice guidelines) will be defined according to the definition outlined in the WHO Handbook for Guideline Development (2012). Eligible reviews will have "a specific and clearly focused question (in PICO format); an explicit, reproducible method including pre-defined eligibility criteria; a comprehensive, exhaustive and systematic search for primary studies; a selection of studies using clear and reproducible eligibility criteria; critical appraisal of included studies for quality; and a systematic presentation and synthesis of the characteristics and findings of the included studies."

Reports will be included regardless of publication status. Only full-text reports will be included; reports in abstract form only will be listed as potentially relevant studies.

Language

For practical consideration, we will include only studies written in English or French in the final cohort of evidence. We will provide a list of potentially relevant records written in other languages for the final report, as our search will not be restricted by language of publication (see below).

Evidence identification and retrieval

Search Methods

Searching will be limited to Ovid MEDLINE database, The Cochrane Infectious Diseases Review Group reviews and Specialized Register, Embase, and African Index Medicus database. MEDLINE is well-known source of medical research information with substantial journal coverage. Embase is another key source of medical research information with substantial journal coverage. For feasibility, we are restricting our inclusion of Cochrane resources to review and resources of the most relevant Cochrane review group, The Cochrane Infectious Diseases Group, who keep a database of relevant studies from bibliographic and handsearching activities. The African Index Medicus will include health information published in or related to Africa and include unpublished information not indexed elsewhere.[i]

The search strategies will include controlled vocabulary (e.g., Medical Subject Headings [MeSH] terms) and text word terms. Searches will not be limited by language.

In addition, a few key grey literature sources (internet websites) will be identified through consultation with the WHO information retrieval specialist staff at WHO headquarters (under the direction of Mr. Tomas Allen). Collaboration with this team is essential to capture regional and country-level data that may be relevant to the ongoing Ebola virus disease outbreak.

Preliminary search strategies (Appendix 1) were developed by a senior Information Specialist with extensive experience developing and executing search strategies for systematic reviews and with graduate library science training. The systematic review team will work with the WHO information retrieval specialist staff to further refine strategies via peer-review using the PRESS guideline.[ii]

For practical consideration, only full text reports that are available electronically to the systematic review team will be retrieved; exclusion of records without available full text reports will documented.

Study Screening and Selection

Citations de-duplicated in Reference Manager will be uploaded into the Distiller Systematic Review (Distiller SR) Software© for Levels 1 and 2 screening. At Level 1 screening, titles and abstracts will be assessed by one reviewer for potential relevance; a second reviewer will verify those records deemed not relevant. At level 2 screening, full-text reports will be assessed for eligibility by two independent reviewers. Disagreements during full-text screening between pairwise reviewers will be resolved through consensus or by a third team member. Reports that are co-publications or multiple reports of the same study will be identified as such. The eligibility criteria apply to all reports, and for secondary sources such as systematic reviews, this will involve comparing their eligibility criteria with that of this rapid review. Only evidence reflective of the rapid review's eligibility criteria within those secondary sources will be included. Screening forms in Distiller SR will be pilot-tested by the systematic review team using a subset of records before implementation.

Data Extraction

One reviewer will extract data and a second reviewer will verify information for a 10% random sample of studies. The following information will be extracted:

Population: demographic variables such as age and gender; type of health care worker; type of health care facility; patient illness

Intervention: description of protective equipment used

Comparator: description of protective equipment used

Outcomes: data as provided in the report

Study description: country; study design; sample size; duration of follow-up; funding source; year of publication or production of report; existence of co-publication or companion report(s)

As above, data extraction forms will be developed in Distiller SR and pilot -tested on a sample of studies by the systematic review team.

Quality assessment and risk of bias

The risk of bias of included reports will be assessed by one reviewer with verification of at least 10% of studies by a second reviewer. Disagreements will be resolved by consensus or third person adjudication. For systematic reviews, the 11-point validated AMSTAR tool (A MeaSurement Tool to Assess Reviews) will be used.[iii].[iv] RCT and nonRCTs will be evaluated according to the Cochrane risk of bias tool.[v] The Cochrane Effective Practice and Organization of Care (EPOC) tool will be used to evaluate the risk of bias of CBS and ITS.[vi] The Newcastle-Ottawa scale will be used for relevant observational designs.

Tools were chosen as Cochrane is an internationally recognized standard for systematic review methodology and AMSTAR has been used by several organizations (e.g., Canadian Agency for Drugs and Technologies in Health: Rx for Change; McMaster's Health Systems Evidence database) and has published validity and reliability.[vii,viii]

Evidence synthesis

The evidence synthesis will be primary descriptive due to the tight timelines imposed by the rapid review process. If time permits and the nature of the data allow for it, we will consider pooling studies using standard meta-analytic techniques. Studies will be pooled only if sufficient number and similar in PICOTS parameters and risk of bias. Decisions with respect to conducting meta-analyses and assessing clinical heterogeneity will be conducted in consultation with the WHO Steering Group.

If evidence from existing systematic reviews is used, we will consider the currency of the review. If it is unlikely that new primary studies are available, then the review will be deemed up-to-date. If feasible, a review that is not current may be updated to include the primary evidence located in the literature. If multiple systematic reviews exist, then they will be examined for methodological rigour, study overlap, and currency to determine whether the focus is placed on one review or whether multiple reviews of similar quality are explored for discordance and discussion of the overlap of included literature.

Quality assessment of the body of evidence for each outcome

The GRADE framework will be considered when judging the quality of the evidence for outcomes that are considered important and critical to decision-making. The extent to which the full GRADE framework can be used will depend on the nature of evidence (systematic reviews versus primary studies) included in the guideline, and also on feasibility. These decisions will be made in discussion with the WHO Steering Group.

References

[i] African Index Medicus [Internet]. Available at http://www.asksource.info/resources/african-index-medicus-aim. Last accessed: 18-07-2014.

[ii] Sampson M, McGowan J, Cogo E, et al. An evidence-based practice guideline for the peer review of electronic search strategies. J Clin Epidemiol 2009 Sep; 62(9): 944-52. PMID: 19230612.

[iii] Shea BJ, Grimshaw JM, Wells GA, et al. Development of AMSTAR: a measurement tool to assess the methodological quality of systematic reviews. BMC Med Res Methodol 2007; 7:10. PMID: 17302989.

[iv] CADTH - Canadian Agency for Drugs and Technologies in Health. Rx for Change: Methods for Development. [Internet]. Available at http://www.cadth.ca/en/resources/rx-for-change/methods-for-development. Last accessed: 18-07-2014.

[v] Higgins J, Altman DG. Assessing risk of bias in included studies. In: Cochrane handbook for systematic reviews of interventions, Wiley Online Library, 2008. p.187-241.

[vi] Cochrane Effective Practice and Organisation of Care Group. Suggested risk of bias criteria for EPOC reviews [Internet]. Available at http://epoc.cochrane.org/sites/epoc.cochrane.org/files/uploads/Suggested%20risk%20of%20bias%20criteria%20for%20EPOC%20reviews.pdf. Last Accessed: 16-07-2014.

[vii] Shea BJ, Hamel C, Wells GA, et al. AMSTAR is a reliable and valid measurement tool to assess the methodological quality of systematic reviews. J Clin Epidemiol. 2009 Oct; 62(10):1013-20. PMID: 19230606.

[viii] Shea BJ, Bouter LM, Peterson J, et al. External Validation of a Measurement Tool to Assess Systematic Reviews (AMSTAR). PLoS ONE. 2007; 2(12): e1350. PMCID: PMC2131785.

ANNEX 1. Preliminary Scoping Search

Our intention to limit the search to studies published or produced from 1976 onwards is not reflected in the following preliminary search strategy.

Database: Ovid MEDLINE(R) In-Process & Other Non-Indexed Citations and Ovid MEDLINE(R) <1946 to Present>

Date of search: 2014 JUL 17

1 expFiloviridae/ (1476)
2 expFiloviridae Infections/ (1118)
3 filovirida*.tw. (141)
4 (Ebolavirus* or Ebola virus*).tw. (1252)
5 (BDBV or EBOV or RESTV or SUDV or TAFV).tw. (295)
6 Hemorrhagic Fever, Ebola/ (801)
7 (ebola* adj3 (disease* or fever* or virus*)).tw. (1446)
8 (marburg* adj3 (disease* or fever* or virus*)).tw. (661)
9 or/1-8 (2362)
10 Communicable Disease Control/ (17820)
11 exp Infection Control/ (50520)
12 exp Cross Infection/pc (19649)
13 exp Disease Outbreaks/pc (12298)
14 exp Disease Transmission, Infectious/pc (9832)
15 exp Viruses/pc (4)
16 ((diseas* or infect* or virus*) adj5 (prevent* or precaution* or control* or eliminat* or manag* or reduc* or stop*)).tw. (338056)
17 ((epidemic* or outbreak* or pandemic*) adj5 (prevent* or precaution* or control* or eliminat* or manag* or reduc* or stop*)).tw. (11405)
18 or/10-17 (418538)
19 9 or 18 (420475)
20 Protective Devices/ (5910)
21 exp Protective Clothing/ (9910)
22 ((precaution* or protect*) adj3 (apparel* or attire* or barrier* or cloth* or device* or equipment* or gear or layer* or material* or workwear)).tw. (11253)
23 PPE.tw. (1849)
24 Eye Protective Devices/ (1480)
25 (eyecover* or eyewear$1 or glasses* or goggle$1).tw. (8562)
26 Masks/ (3353)
27 (mask$1 or facemask* or facialmask* or faceshield* or facialshield*).tw. (25208)
28 ((eye or eyes or face or faces or facial* or ear or ears or mouth$1 or body or bodies or skin) adj3 (cover* or hygien* or precaution* or protect* or shield*)).tw. (11736)
29 (glove$1 or gloving or single-glov* or double-glov* or triple-glov*).tw. (7747)
30 Surgical Attire/ (18)
31 (apron* or gown or gowns or scrubs or theatre blues).tw. (1708)
32 ((surgical or surger*) adj3 (apparel* or attire* or cloth* or workwear)).tw. (89)
33 exp Occupational exposure/pc (4840)

34 (occupation* adj3 expos* adj5 (prevent* or precaution* or control* or eliminat* or manag* or reduc* or stop*)).tw. (1293)
35 ((personnel* or HCW or HCWs or worker* or health professional*) adj3 expos* adj5 (prevent* or precaution* or control* or eliminat* or manag* or reduc* or stop*)).tw. (1541)
36 (occupational adj3 (hygiene or safety)).tw. (5450)
37 Universal precautions/ (1488)
38 ((safety or universal) adj3 precaution*).tw. (1844)
39 Equipment Contamination/pc (3104)
40 (contaminat* adj5 (prevent* or precaution* or control* or eliminat* or manag* or reduc* or stop*)).tw. (10832)
41 or/20-40 (100692)
42 19 and 41 (11180)
43 exp Animals/ not (exp Animals/ and Humans/) (3968668)
44 42 not 43 (10698)
45 limit 44 to systematic reviews (426)
46 meta analysis.pt. (49821)
47 exp meta-analysis as topic/ (13890)
48 (meta-analy* or metanaly* or metaanaly* or met analy* or integrative research or integrative review* or integrative overview* or research integration or research overview* or collaborative review*).tw. (68176)
49 (systematic review* or systematic overview* or evidence-based review* or evidence-based overview* or (evidence adj3 (review* or overview*)) or meta-review* or meta-overview* or meta-synthes* or "review of reviews" or technology assessment* or HTA or HTAs).tw. (85290)
50 exp Technology assessment, biomedical/ (9209)
51 (cochrane or health technology assessment or evidence report).jw. (12486)
52 or/46-51 (162164)
53 44 and 52 (177)
54 45 or 53 (484) [REVIEWS]
55 exp Guidelines as Topic/ (115154)
56 exp Clinical Protocols/ (125416)
57 Guideline.pt. (15581)
58 Practice Guideline.pt. (19300)
59 Consensus Development Conference.pt. (9183)
60 (guideline* or standards or recommendation*).ti. (91316)
61 or/55-60 (313894)
62 44 and 61 (1118) [GUIDELINES]
63 (controlled clinical trial or randomized controlled trial).pt. (462935)
64 clinical trials as topic.sh. (171051)
65 (randomi#ed or randomly or RCT$1 or placebo*).tw. (630600)
66 ((singl* or doubl* or trebl* or tripl*) adj (mask* or blind* or dumm*)).tw. (131133)
67 trial.ti. (128804)
68 or/63-67 (952414)
69 44 and 68 (684) [RCTS]
70 controlled clinical trial.pt. (88847)
71 Controlled Clinical Trial/ or Controlled Clinical Trials as Topic/ (93809)
72 (control* adj2 trial*).tw. (147510)
73 (nonrandom* or non-random* or quasi-random* or quasi-experiment*).tw. (34302)

74 (nRCT or nRCTs or non-RCT$1).tw. (286)
75 (control* adj3 ("before and after" or "before after")).tw. (2710)
76 time series.tw. (16201)
77 (pre- adj3 post-).tw. (43458)
78 (pretest adj3 posttest).tw. (3013)
79 (control* adj2 stud$3).tw. (156342)
80 Control Groups/ (1468)
81 (control$ adj2 group$1).tw. (322094)
82 or/70-81 (733221)
83 44 and 82 (630) [NON-RCTS]
84 54 or 62 or 69 or 83 (2280)
85 (comment or editorial or interview or letter or news).pt. (1523008)
86 84 not 85 (2195)
