# Supplementary material for: Effectiveness of Personal Protective Equipment for Healthcare Workers Caring for Patients with Filovirus Disease: A Rapid Review
Source: PLoS One. 2015 Oct 9;10(10):e0140290. doi: 10.1371/journal.pone.0140290 (PMC4599797; doi:10.1371/journal.pone.0140290)
Supplement: S3 Appendix — (DOCX) [file pone.0140290.s003.docx]

| **S3 Appendix. Search Strategy** | | |
| --- | --- | --- |
| **Ovid MEDLINE®**  **Ovid MEDLINE® In-Process & Other Non-Indexed Citations** | | |
| **Virus Type:**Filoviruses (Ebola, Marburg)  **PPE:** Gloves, Face protection and Gowns  **Search Date:**July 28, 2014  **Study Design Types:** Systematic reviews, Meta-Analyses, Health Technology Assessments, Guidelines,  Protocols, Randomized Controlled Trials, Quasi-Experimental designs, Comparative  Cohort and Case-Control Studies, Single Arm Cohorts. | | |
| 1 | | expFiloviridae/ (1478) |
| 2 | | expFiloviridae Infections/ (1120) |
| 3 | | filovirida*.tw. (141) |
| 4 | | (Ebolavir* or Ebola vir* or Marburgvir* or Marburg vir* or Filovir* or Filo vir*).tw. (1784) |
| 5 | | (BDBV or EBOV or RESTV or SUDV or TAFV).tw. (297) |
| 6 | | Hemorrhagic Fever, Ebola/ (803) |
| 7 | | (ebola* adj10 (disease* or fever* or infect* or strain$1 or virus* or epidemic* or outbreak* or pandemic*)).tw. (1654) |
| 8 | | (marburg* adj10 (disease* or fever* or infect* or strain$1 or virus* or epidemic* or outbreak* or pandemic*)).tw. (882) |
| 9 | | or/1-8 (2623) |
| 10 | | Communicable Disease Control/ (17830) |
| 11 | | exp Infection Control/ (50544) |
| 12 | | exp Cross Infection/pc (19658) |
| 13 | | exp Disease Outbreaks/pc (12307) |
| 14 | | exp Disease Transmission, Infectious/pc (9844) |
| 15 | | exp Virus Diseases/pc (124976) |
| 16 | | ((diseas* or infect* or virus*) adj5 (prevent* or precaution* or control* or eliminat* or manag* or reduc* or stop*)).tw. (338816) |
| 17 | | ((epidemic* or outbreak* or pandemic*) adj5 (prevent* or precaution* or control* or eliminat* or manag* or reduc* or stop*)).tw. (11439) |
| 18 | | or/10-17 (509452) |
| 19 | | 9 and 18 (700) |
| 20 | | Protective Devices/ (5914) |
| 21 | | exp Protective Clothing/ (9917) |
| 22 | | ((precaution* or protect* or prophyla*) adj3 (apparel* or attire* or barrier* or cloth* or device* or equipment* or garment* or gear or layer* or material* or workwear)).tw. (11645) |
| 23 | | PPE.tw. (1857) |
| 24 | | Eye Protective Devices/ (1481) |
| 25 | | (eyecover* or eyeglasses or eyevisor* or eyewear* or glasses* or goggle$1 or (eye$1 adj2 visor*)).tw. (9077) |
| 26 | | Masks/ (3354) |
| 27 | | (mask$1 or facemask* or facialmask* or faceseal* or facialseal* or faceshield* or facialshield*).tw. (25264) |
| 28 | | ((eye or eyes or face or faces or facial* or ear or ears or mouth$1 or body or bodies or skin or hand or hands) adj3 (cover* or hygien* or precaution* or protect* or prophyla* or seal* or shield*)).tw. (15834) |
| 29 | | (glove$1 or gloving or single-glov* or double-glov* or triple-glov*).tw. (7760) |
| 30 | | Surgical Attire/ (18) |
| 31 | | (apron* or gown or gowns or scrubs or theatre blues).tw. (1710) |
| 32 | | ((surgical or surger*) adj3 (apparel* or attire* or cloth* or garment* or workwear)).tw. (99) |
| 33 | | exp Occupational exposure/pc (4848) |
| 34 | | (occupation* adj3 (expos* or contaminat* or infect* or transmission* or transmit*) adj5 (prevent* or precaution* or control* or eliminat* or manag* or reduc* or stop*)).tw. (1507) |
| 35 | | ((personnel* or HCW or HCWs or worker* or (health* adj3 professional*) or clinician* or doctor* or nurse* or physician* or practitioner*) adj3 (expos* or contaminat* or infect* or transmission* or transmit*) adj5 (prevent* or precaution* or control* or eliminat* or manag* or reduc* or stop*)).tw. (3166) |
| 36 | | (occupational adj3 (hygiene or safety)).tw. (5457) |
| 37 | | Universal precautions/ (1488) |
| 38 | | ((safety or standard* or universal) adj3 precaution*).tw. (2340) |
| 39 | | Equipment Contamination/pc (3105) |
| 40 | | (contaminat* adj5 (prevent* or precaution* or control* or eliminat* or manag* or reduc* or stop*)).tw. (10855) |
| 41 | | or/20-40 (106637) |
| 42 | | 9 and 41 (51) |
| 43 | | 19 or 42 (720) |
| 44 | | exp Animals/ not (exp Animals/ and Humans/) (3970931) |
| 45 | | 43 not 44 (605) |
| 46 | | limit 45 to systematic reviews (7) |
| 47 | | meta analysis.pt. (49929) |
| 48 | | exp meta-analysis as topic/ (13901) |
| 49 | | (meta-analy* or metanaly* or metaanaly* or met analy* or integrative research or integrative review* or integrative overview* or research integration or research overview* or collaborative review*).tw. (68624) |
| 50 | | (systematic review* or systematic overview* or evidence-based review* or evidence-based overview* or (evidence adj3 (review* or overview*)) or meta-review* or meta-overview* or meta-synthes* or "review of reviews" or technology assessment* or HTA or HTAs).tw. (85760) |
| 51 | | exp Technology assessment, biomedical/ (9216) |
| 52 | | (cochrane or health technology assessment or evidence report).jw. (12492) |
| 53 | | or/47-52 (162936) |
| 54 | | 45 and 53 (1) |
| 55 | | 46 or 54 (7) |
| 56 | | exp Guidelines as Topic/ (115325) |
| 57 | | exp Clinical Protocols/ (125537) |
| 58 | | Guideline.pt. (15585) |
| 59 | | Practice Guideline.pt. (19311) |
| 60 | | Consensus Development Conference.pt. (9187) |
| 61 | | (guideline* or standards or recommendation*).ti. (91508) |
| 62 | | or/56-61 (314324) |
| 63 | | 45 and 62 (21) |
| 64 | | (controlled clinical trial or randomized controlled trial).pt. (463434) |
| 65 | | clinical trials as topic.sh. (171159) |
| 66 | | (randomi#ed or randomly or RCT$1 or placebo*).tw. (632465) |
| 67 | | ((singl* or doubl* or trebl* or tripl*) adj (mask* or blind* or dumm*)).tw. (131354) |
| 68 | | trial.ti. (129236) |
| 69 | | or/64-68 (954580) |
| 70 | | 45 and 69 (11) |
| 71 | | controlled clinical trial.pt. (88883) |
| 72 | | Controlled Clinical Trial/ or Controlled Clinical Trials as Topic/ (93847) |
| 73 | | (control* adj2 trial*).tw. (148093) |
| 74 | | (nonrandom* or non-random* or quasi-random* or quasi-experiment*).tw. (34397) |
| 75 | | (nRCT or nRCTs or non-RCT$1).tw. (287) |
| 76 | | (control* adj3 ("before and after" or "before after")).tw. (2720) |
| 77 | | time series.tw. (16255) |
| 78 | | (pre- adj3 post-).tw. (43617) |
| 79 | | (pretest adj3 posttest).tw. (3027) |
| 80 | | (control* adj2 stud$3).tw. (156764) |
| 81 | | Control Groups/ (1468) |
| 82 | | (control$ adj2 group$1).tw. (323086 |
| 83 | | or/71-82 (735252) |
| 84 | | 45 and 83 (8) |
| 85 | | exp Cohort Studies/ (1369432) |
| 86 | | cohort$1.tw. (286487) |
| 87 | | (longitudinal or prospective or retrospective).tw. (748230) |
| 88 | | ((followup or follow-up) adj (study or studies)).tw. (38581) |
| 89 | | exp Case-Control Studies/ (672213) |
| 90 | | ((case-control* or case-based or case-comparison) adj (study or studies)).tw. (67082) |
| 91 | | or/85-90 (1886383) |
| 92 | | 45 and 91 (18) |
| 93 | | 55 or 63 or 70 or 84 or 92 (56) |
| 94 | | (comment or editorial or interview or letter or news).pt. (1525448) |
| 95 | | 93 not 94 (52) |
|  | | |
| **Virus Type:** Filoviruses (Ebola, Marburg)  **PPE:** Gloves, Face protection and Gowns  **Search Date:** July 31, 2014  **Study Design Types:** Case-Reports and Cross-Sectional Design | | |
| 1 | | exp Filoviridae/ (1478) |
| 2 | | exp Filoviridae Infections/ (1121) |
| 3 | | filovirida*.tw. (141) |
| 4 | | (Ebolavir* or Ebola vir* or Marburgvir* or Marburg vir* or Filovir* or Filo vir*).tw. (1787) |
| 5 | | (BDBV or EBOV or RESTV or SUDV or TAFV).tw. (297) |
| 6 | | Hemorrhagic Fever, Ebola/ (804) |
| 7 | | (ebola* adj10 (disease* or fever* or infect* or strain$1 or virus* or epidemic* or outbreak* or pandemic*)).tw. (1659) |
| 8 | | (marburg* adj10 (disease* or fever* or infect* or strain$1 or virus* or epidemic* or outbreak* or pandemic*)).tw. (882) |
| 9 | | or/1-8 (2629) |
| 10 | | Communicable Disease Control/ (17834) |
| 11 | | exp Infection Control/ (50561) |
| 12 | | exp Cross Infection/pc (19665) |
| 13 | | exp Disease Outbreaks/pc (12313) |
| 14 | | exp Disease Transmission, Infectious/pc (9848) |
| 15 | | exp Virus Diseases/pc (125054) |
| 16 | | ((diseas* or infect* or virus*) adj5 (prevent* or precaution* or control* or eliminat* or manag* or reduc* or stop*)).tw. (339078) |
| 17 | | ((epidemic* or outbreak* or pandemic*) adj5 (prevent* or precaution* or control* or eliminat* or manag* or reduc* or stop*)).tw. (11438) |
| 18 | | or/10-17 (509805) |
| 19 | | 9 and 18 (702) |
| 20 | | Protective Devices/ (5916) |
| 21 | | exp Protective Clothing/ (9923) |
| 22 | | ((precaution* or protect* or prophyla*) adj3 (apparel* or attire* or barrier* or cloth* or device* or equipment* or garment* or gear or layer* or material* or workwear)).tw. (11651) |
| 23 | | PPE.tw. (1858) |
| 24 | | Eye Protective Devices/ (1481) |
| 25 | | (eyecover* or eyeglasses or eyevisor* or eyewear* or glasses* or goggle$1 or (eye$1 adj2 visor*)).tw. (9080) |
| 26 | | Masks/ (3356) |
| 27 | | (mask$1 or facemask* or facialmask* or faceseal* or facialseal* or faceshield* or facialshield*).tw. (25285) |
| 28 | | ((eye or eyes or face or faces or facial* or ear or ears or mouth$1 or body or bodies or skin or hand or hands) adj3 (cover* or hygien* or precaution* or protect* or prophyla* or seal* or shield*)).tw. (15841) |
| 29 | | (glove$1 or gloving or single-glov* or double-glov* or triple-glov*).tw. (7762) |
| 30 | | Surgical Attire/ (18) |
| 31 | | (apron* or gown or gowns or scrubs or theatre blues).tw. (1713) |
| 32 | | ((surgical or surger*) adj3 (apparel* or attire* or cloth* or garment* or workwear)).tw. (99) |
| 33 | | exp Occupational exposure/pc (4854) |
| 34 | | (occupation* adj3 (expos* or contaminat* or infect* or transmission* or transmit*) adj5 (prevent* or precaution* or control* or eliminat* or manag* or reduc* or stop*)).tw. (1506) |
| 35 | | ((personnel* or HCW or HCWs or worker* or (health* adj3 professional*) or clinician* or doctor* or nurse* or physician* or practitioner*) adj3 (expos* or contaminat* or infect* or transmission* or transmit*) adj5 (prevent* or precaution* or control* or eliminat* or manag* or reduc* or stop*)).tw. (3167) |
| 36 | | (occupational adj3 (hygiene or safety)).tw. (5466) |
| 37 | | Universal precautions/ (1488) |
| 38 | | ((safety or standard* or universal) adj3 precaution*).tw. (2339) |
| 39 | | Equipment Contamination/pc (3106) |
| 40 | | (contaminat* adj5 (prevent* or precaution* or control* or eliminat* or manag* or reduc* or stop*)).tw. (10859) |
| 41 | | or/20-40 (106715) |
| 42 | | 9 and 41 (51) |
| 43 | | 19 or 42 (722) |
| 44 | | exp Animals/ not (exp Animals/ and Humans/) (3972902) |
| 45 | | 43 not 44 (607) |
| 46 | | limit 45 to systematic reviews (7) |
| 47 | | meta analysis.pt. (50023) |
| 48 | | exp meta-analysis as topic/ (13910) |
| 49 | | (meta-analy* or metanaly* or metaanaly* or met analy* or integrative research or integrative review* or integrative overview* or research integration or research overview* or collaborative review*).tw. (68777) |
| 50 | | (systematic review* or systematic overview* or evidence-based review* or evidence-based overview* or (evidence adj3 (review* or overview*)) or meta-review* or meta-overview* or meta-synthes* or "review of reviews" or technology assessment* or HTA or HTAs).tw. (85916) |
| 51 | | exp Technology assessment, biomedical/ (9235) |
| 52 | | (cochrane or health technology assessment or evidence report).jw. (12488) |
| 53 | | or/47-52 (163213) |
| 54 | | 45 and 53 (1) |
| 55 | | 6 or 54 (7) |
| 56 | | exp Guidelines as Topic/ (115457) |
| 57 | | exp Clinical Protocols/ (125681) |
| 58 | | Guideline.pt. (15585) |
| 59 | | Practice Guideline.pt. (19336) |
| 60 | | Consensus Development Conference.pt. (9198) |
| 61 | | (guideline* or standards or recommendation*).ti. (91559) |
| 62 | | or/56-61 (314621) |
| 63 | | 45 and 62 (21) |
| 64 | | (controlled clinical trial or randomized controlled trial).pt. (463758) |
| 65 | | clinical trials as topic.sh. (171230) |
| 66 | | (randomi#ed or randomly or RCT$1 or placebo*).tw. (633036) |
| 67 | | ((singl* or doubl* or trebl* or tripl*) adj (mask* or blind* or dumm*)).tw. (131432) |
| 68 | | trial.ti. (129405) |
| 69 | | or/64-68 (955346) |
| 70 | | 45 and 69 (11) |
| 71 | | controlled clinical trial.pt. (88919) |
| 72 | | Controlled Clinical Trial/ or Controlled Clinical Trials as Topic/ (93885) |
| 73 | | (control* adj2 trial*).tw. (148259) |
| 74 | | (nonrandom* or non-random* or quasi-random* or quasi-experiment*).tw. (34429) |
| 75 | | (nRCT or nRCTs or non-RCT$1).tw. (287) |
| 76 | | (control* adj3 ("before and after" or "before after")).tw. (2721) |
| 77 | | time series.tw. (16273) |
| 78 | | (pre- adj3 post-).tw. (43648) |
| 79 | | (pretest adj3 posttest).tw. (3030) |
| 80 | | (control* adj2 stud$3).tw. (156907) |
| 81 | | Control Groups/ (1468) |
| 82 | | (control$ adj2 group$1).tw. (323397) |
| 83 | | or/71-82 (735918) |
| 84 | | 45 and 83 (8) |
| 85 | | exp Cohort Studies/ (1370879) |
| 86 | | cohort$1.tw. (286854) |
| 87 | | (longitudinal or prospective or retrospective).tw. (749021) |
| 88 | | ((followup or follow-up) adj (study or studies)).tw. (38587) |
| 89 | | exp Case-Control Studies/ (673186) |
| 90 | | ((case-control* or case-based or case-comparison) adj (study or studies)).tw. (67167) |
| 91 | | or/85-90 (1888261) |
| 92 | | 45 and 91 (18) |
| 93 | | 55 or 63 or 70 or 84 or 92 (56) |
| 94 | | (comment or editorial or interview or letter or news).pt. (1526324) |
| 95 | | 93 not 94 (52) |
| 96 | | Case Reports.pt. (1693418) |
| 97 | | (case adj (report$1 or study or studies or history or histories or series)).tw. (341362) |
| 98 | | Cross-Sectional Studies/ (179862) |
| 99 | | (cross-section* adj3 (study or studies or analy* or survey*)).tw. (126814) |
| 100 | | or/95-99 (2042383) |
| 101 | | 45 and 100 (70) |
| 102 | | 101 not 93 (18) |
| 103 | | 102 not 94 (17) |
|  | | |
| **Virus Type:**Filoviruses & Viral Hemorrhagic Fevers  **PPE:** Gloves, Face Protection and Gowns  **Search Date:** July 28, 2014  **Study Design Types:** Systematic reviews, Meta-Analyses, Health Technology Assessments, Guidelines,  Protocols, Randomized Controlled Trials, Quasi-Experimental designs, Comparative  Cohort and Case-Control Studies, Single Arm Cohorts. | | |
| 1 | | expFiloviridae/ (1478) |
| 2 | | expFiloviridae Infections/ (1120) |
| 3 | | filovirida*.tw. (141) |
| 4 | | (Ebolavir* or Ebola vir* or Marburgvir* or Marburg vir* or Filovir* or Filo vir*).tw. (1784) |
| 5 | | (BDBV or EBOV or RESTV or SUDV or TAFV).tw. (297) |
| 6 | | Hemorrhagic Fever, Ebola/ (803) |
| 7 | | (ebola* adj10 (disease* or fever* or infect* or strain$1 or virus* or epidemic* or outbreak* or pandemic*)).tw. (1654) |
| 8 | | Marburg Virus Disease/ (358) |
| 9 | | (marburg* adj10 (disease* or fever* or infect* or strain$1 or virus* or epidemic* or outbreak* or pandemic*)).tw. (882) |
| 10 | | Hemorrhagic Fevers, Viral/ (1130) |
| 11 | | (h?emorrhagic fever$1 adj10 (virus* or viral*)).tw. (2565) |
| 12 | | Nairovirus/ (38) |
| 13 | | (nairovir* or nairovir* or dugbevir* or dugbevir*).tw. (170) |
| 14 | | Hemorrhagic Fever Virus, Crimean-Congo/ (506) |
| 15 | | Hemorrhagic Fever, Crimean/ (572) |
| 16 | | ((congo* or crimean*) adj10 (disease* or fever* or infect* or strain$1 or virus* or epidemic* or outbreak* or pandemic*)).tw. (2672) |
| 17 | | Arenaviridae Infections/ (477) |
| 18 | | (arenavir* or arena vir*).tw. (1025) |
| 19 | | Lassa Fever/ (456) |
| 20 | | Lassa virus/ (479) |
| 21 | | (lassa adj10 (disease* or fever* or infect* or strain$1 or virus* or epidemic* or outbreak* or pandemic*)).tw. (847) |
| 22 | | Cholera/ (7310) |
| 23 | | Vibrio cholerae/ (6859) |
| 24 | | (cholera* or choleric*).tw. (25676) |
| 25 | | or/1-24 (36397) |
| 26 | | Communicable Disease Control/ (17830) |
| 27 | | exp Infection Control/ (50544) |
| 28 | | exp Cross Infection/pc (19658) |
| 29 | | exp Disease Outbreaks/pc (12307) |
| 30 | | exp Disease Transmission, Infectious/pc (9844) |
| 31 | | exp Virus Diseases/pc (124976) |
| 32 | | ((diseas* or infect* or virus*) adj5 (prevent* or precaution* or control* or eliminat* or manag* or reduc* or stop*)).tw. (338816) |
| 33 | | ((epidemic* or outbreak* or pandemic*) adj5 (prevent* or precaution* or control* or eliminat* or manag* or reduc* or stop*)).tw. (11439) |
| 34 | | or/26-33 (509452) |
| 35 | | 25 and 34 (3490) |
| 36 | | Protective Devices/ (5914) |
| 37 | | exp Protective Clothing/ (9917) |
| 38 | | ((precaution* or protect* or prophyla*) adj3 (apparel* or attire* or barrier* or cloth* or device* or equipment* or garment* or gear or layer* or material* or workwear)).tw. (11645) |
| 39 | | PPE.tw. (1857) |
| 40 | | Eye Protective Devices/ (1481) |
| 41 | | (eyecover* or eyeglasses or eyevisor* or eyewear* or glasses* or goggle$1 or (eye$1 adj2 visor*)).tw. (9077) |
| 42 | | Masks/ (3354) |
| 43 | | (mask$1 or facemask* or facialmask* or faceseal* or facialseal* or faceshield* or facialshield*).tw. (25264) |
| 44 | | ((eye or eyes or face or faces or facial* or ear or ears or mouth$1 or body or bodies or skin or hand or hands) adj3 (cover* or hygien* or precaution* or protect* or prophyla* or seal* or shield*)).tw. (15834) |
| 45 | | (glove$1 or gloving or single-glov* or double-glov* or triple-glov*).tw. (7760) |
| 46 | | Surgical Attire/ (18) |
| 47 | | (apron* or gown or gowns or scrubs or theatre blues).tw. (1710) |
| 48 | | ((surgical or surger*) adj3 (apparel* or attire* or cloth* or garment* or workwear)).tw. (99) |
| 49 | | exp Occupational exposure/pc (4848) |
| 50 | | (occupation* adj3 (expos* or contaminat* or infect* or transmission* or transmit*) adj5 (prevent* or precaution* or control* or eliminat* or manag* or reduc* or stop*)).tw. (1507) |
| 51 | | ((personnel* or HCW or HCWs or worker* or (health* adj3 professional*) or clinician* or doctor* or nurse* or physician* or practitioner*) adj3 (expos* or contaminat* or infect* or transmission* or transmit*) adj5 (prevent* or precaution* or control* or eliminat* or manag* or reduc* or stop*)).tw. (3166) |
| 52 | | (occupational adj3 (hygiene or safety)).tw. (5457) |
| 53 | | Universal precautions/ (1488) |
| 54 | | ((safety or standard* or universal) adj3 precaution*).tw. (2340) |
| 55 | | Equipment Contamination/pc (3105) |
| 56 | | (contaminat* adj5 (prevent* or precaution* or control* or eliminat* or manag* or reduc* or stop*)).tw. (10855) |
| 57 | | or/36-56 (106637) |
| 58 | | 25 and 57 (190) |
| 59 | | 35 or 58 (3592) |
| 60 | | exp Animals/ not (exp Animals/ and Humans/) (3970931) |
| 61 | | 59 not 60 (2707) |
| 62 | | limit 61 to systematic reviews (24) |
| 63 | | meta analysis.pt. (49929) |
| 64 | | exp meta-analysis as topic/ (13901) |
| 65 | | (meta-analy* or metanaly* or metaanaly* or met analy* or integrative research or integrative review* or integrative overview* or research integration or research overview* or collaborative review*).tw. (68624) |
| 66 | | (systematic review* or systematic overview* or evidence-based review* or evidence-based overview* or (evidence adj3 (review* or overview*)) or meta-review* or meta-overview* or meta-synthes* or "review of reviews" or technology assessment* or HTA or HTAs).tw. (85760) |
| 67 | | exp Technology assessment, biomedical/ (9216) |
| 68 | | (cochrane or health technology assessment or evidence report).jw. (12492) |
| 69 | | or/63-68 (162936) |
| 70 | | 61 and 69 (7) |
| 71 | | 62 or 70 (24) |
| 72 | | exp Guidelines as Topic/ (115325) |
| 73 | | exp Clinical Protocols/ (125537) |
| 74 | | Guideline.pt. (15585) |
| 75 | | Practice Guideline.pt. (19311) |
| 76 | | Consensus Development Conference.pt. (9187) |
| 77 | | (guideline* or standards or recommendation*).ti. (91508) |
| 78 | | or/72-77 (314324) |
| 79 | | 61 and 78 (65) |
| 80 | | (controlled clinical trial or randomized controlled trial).pt. (463434) |
| 81 | | clinical trials as topic.sh. (171159) |
| 82 | | (randomi#ed or randomly or RCT$1 or placebo*).tw. (632465) |
| 83 | | ((singl* or doubl* or trebl* or tripl*) adj (mask* or blind* or dumm*)).tw. (131354) |
| 84 | | trial.ti. (129236) |
| 85 | | or/80-84 (954580) |
| 86 | | 61 and 85 (74) |
| 87 | | controlled clinical trial.pt. (88883) |
| 88 | | Controlled Clinical Trial/ or Controlled Clinical Trials as Topic/ (93847) |
| 89 | | (control* adj2 trial*).tw. (148093) |
| 90 | | (nonrandom* or non-random* or quasi-random* or quasi-experiment*).tw. (34397) |
| 91 | | (nRCT or nRCTs or non-RCT$1).tw. (287) |
| 92 | | (control* adj3 ("before and after" or "before after")).tw. (2720) |
| 93 | | time series.tw. (16255) |
| 94 | | (pre- adj3 post-).tw. (43617) |
| 95 | | (pretest adj3 posttest).tw. (3027) |
| 96 | | (control* adj2 stud$3).tw. (156764) |
| 97 | | Control Groups/ (1468) |
| 98 | | (control$ adj2 group$1).tw. (323086) |
| 99 | | or/87-98 (735252) |
| 100 | | 61 and 99 (109) |
| 101 | | exp Cohort Studies/ (1369432) |
| 102 | | cohort$1.tw. (286487) |
| 103 | | (longitudinal or prospective or retrospective).tw. (748230) |
| 104 | | ((followup or follow-up) adj (study or studies)).tw. (38581) |
| 105 | | exp Case-Control Studies/ (672213) |
| 106 | | ((case-control* or case-based or case-comparison) adj (study or studies)).tw. (67082) |
| 107 | | or/101-106 (1886383) |
| 108 | | 61 and 107 (140) |
| 109 | | 71 or 79 or 86 or 100 or 108 (306) |
| 110 | | (comment or editorial or interview or letter or news).pt. (1525448) |
| 111 | | 109 not 110 (297) |
|  | | |
| **Virus Type:**Filoviruses & Viral Hemorrhagic Fevers  **PPE:** Gloves, Face Protection and Gowns  **Search Date:** July 31, 2014  **Study Design Types:** Case-Reports and Cross-Sectional Design | | |
| 1 | | expFiloviridae/ (1478) |
| 2 | | expFiloviridae Infections/ (1121) |
| 3 | | filovirida*.tw. (141) |
| 4 | | (Ebolavir* or Ebola vir* or Marburgvir* or Marburg vir* or Filovir* or Filo vir*).tw. (1787) |
| 5 | | (BDBV or EBOV or RESTV or SUDV or TAFV).tw. (297) |
| 6 | | Hemorrhagic Fever, Ebola/ (804) |
| 7 | | (ebola* adj10 (disease* or fever* or infect* or strain$1 or virus* or epidemic* or outbreak* or pandemic*)).tw. (1659) |
| 8 | | Marburg Virus Disease/ (358) |
| 9 | | (marburg* adj10 (disease* or fever* or infect* or strain$1 or virus* or epidemic* or outbreak* or pandemic*)).tw. (882) |
| 10 | | Hemorrhagic Fevers, Viral/ (1130) |
| 11 | | (h?emorrhagic fever$1 adj10 (virus* or viral*)).tw. (2574) |
| 12 | | Nairovirus/ (39) |
| 13 | | (nairovir* or nairovir* or dugbevir* or dugbevir*).tw. (172) |
| 14 | | Hemorrhagic Fever Virus, Crimean-Congo/ (506) |
| 15 | | Hemorrhagic Fever, Crimean/ (572) |
| 16 | | ((congo* or crimean*) adj10 (disease* or fever* or infect* or strain$1 or virus* or epidemic* or outbreak* or pandemic*)).tw. (2675) |
| 17 | | Arenaviridae Infections/ (477) |
| 18 | | (arenavir* or arena vir*).tw. (1029) |
| 19 | | Lassa Fever/ (456) |
| 20 | | Lassa virus/ (479) |
| 21 | | (lassa adj10 (disease* or fever* or infect* or strain$1 or virus* or epidemic* or outbreak* or pandemic*)).tw. (851) |
| 22 | | Cholera/ (7311) |
| 23 | | Vibrio cholerae/ (6863) |
| 24 | | (cholera* or choleric*).tw. (25694) |
| 25 | | or/1-24 (36431) |
| 26 | | Communicable Disease Control/ (17834) |
| 27 | | exp Infection Control/ (50561) |
| 28 | | exp Cross Infection/pc (19665) |
| 29 | | exp Disease Outbreaks/pc (12313) |
| 30 | | exp Disease Transmission, Infectious/pc (9848) |
| 31 | | exp Virus Diseases/pc (125054) |
| 32 | | ((diseas* or infect* or virus*) adj5 (prevent* or precaution* or control* or eliminat* or manag* or reduc* or stop*)).tw. (339078) |
| 33 | | ((epidemic* or outbreak* or pandemic*) adj5 (prevent* or precaution* or control* or eliminat* or manag* or reduc* or stop*)).tw. (11438) |
| 34 | | or/26-33 (509805) |
| 35 | | 25 and 34 (3492) |
| 36 | | Protective Devices/ (5916) |
| 37 | | exp Protective Clothing/ (9923) |
| 38 | | ((precaution* or protect* or prophyla*) adj3 (apparel* or attire* or barrier* or cloth* or device* or equipment* or garment* or gear or layer* or material* or workwear)).tw. (11651) |
| 39 | | PPE.tw. (1858) |
| 40 | | Eye Protective Devices/ (1481) |
| 41 | | (eyecover* or eyeglasses or eyevisor* or eyewear* or glasses* or goggle$1 or (eye$1 adj2 visor*)).tw. (9080) |
| 42 | | Masks/ (3356) |
| 43 | | (mask$1 or facemask* or facialmask* or faceseal* or facialseal* or faceshield* or facialshield*).tw. (25285) |
| 44 | | ((eye or eyes or face or faces or facial* or ear or ears or mouth$1 or body or bodies or skin or hand or hands) adj3 (cover* or hygien* or precaution* or protect* or prophyla* or seal* or shield*)).tw. (15841) |
| 45 | | (glove$1 or gloving or single-glov* or double-glov* or triple-glov*).tw. (7762) |
| 46 | | Surgical Attire/ (18) |
| 47 | | (apron* or gown or gowns or scrubs or theatre blues).tw. (1713) |
| 48 | | ((surgical or surger*) adj3 (apparel* or attire* or cloth* or garment* or workwear)).tw. (99) |
| 49 | | exp Occupational exposure/pc (4854) |
| 50 | | (occupation* adj3 (expos* or contaminat* or infect* or transmission* or transmit*) adj5 (prevent* or precaution* or control* or eliminat* or manag* or reduc* or stop*)).tw. (1506) |
| 51 | | ((personnel* or HCW or HCWs or worker* or (health* adj3 professional*) or clinician* or doctor* or nurse* or physician* or practitioner*) adj3 (expos* or contaminat* or infect* or transmission* or transmit*) adj5 (prevent* or precaution* or control* or eliminat* or manag* or reduc* or stop*)).tw. (3167) |
| 52 | | (occupational adj3 (hygiene or safety)).tw. (5466) |
| 53 | | Universal precautions/ (1488) |
| 54 | | ((safety or standard* or universal) adj3 precaution*).tw. (2339) |
| 55 | | Equipment Contamination/pc (3106) |
| 56 | | (contaminat* adj5 (prevent* or precaution* or control* or eliminat* or manag* or reduc* or stop*)).tw. (10859) |
| 57 | | or/36-56 (106715) |
| 58 | | 25 and 57 (190) |
| 59 | | 35 or 58 (3594) |
| 60 | | exp Animals/ not (exp Animals/ and Humans/) (3972902) |
| 61 | | 59 not 60 (2708) |
| 62 | | limit 61 to systematic reviews (24) |
| 63 | | meta analysis.pt. (50023) |
| 64 | | exp meta-analysis as topic/ (13910) |
| 65 | | (meta-analy* or metanaly* or metaanaly* or met analy* or integrative research or integrative review* or integrative overview* or research integration or research overview* or collaborative review*).tw. (68777) |
| 66 | | (systematic review* or systematic overview* or evidence-based review* or evidence-based overview* or (evidence adj3 (review* or overview*)) or meta-review* or meta-overview* or meta-synthes* or "review of reviews" or technology assessment* or HTA or HTAs).tw. (85916) |
| 67 | | exp Technology assessment, biomedical/ (9235) |
| 68 | | (cochrane or health technology assessment or evidence report).jw. (12488) |
| 69 | | or/63-68 (163213) |
| 70 | | 61 and 69 (7) |
| 71 | | 62 or 70 (24) |
| 72 | | exp Guidelines as Topic/ (115457) |
| 73 | | exp Clinical Protocols/ (125681) |
| 74 | | Guideline.pt. (15585) |
| 75 | | Practice Guideline.pt. (19336) |
| 76 | | Consensus Development Conference.pt. (9198) |
| 77 | | (guideline* or standards or recommendation*).ti. (91559) |
| 78 | | or/72-77 (314621) |
| 79 | | 61 and 78 (65) |
| 80 | | (controlled clinical trial or randomized controlled trial).pt. (463758) |
| 81 | | clinical trials as topic.sh. (171230) |
| 82 | | (randomi#ed or randomly or RCT$1 or placebo*).tw. (633036) |
| 83 | | ((singl* or doubl* or trebl* or tripl*) adj (mask* or blind* or dumm*)).tw. (131432) |
| 84 | | trial.ti. (129405) |
| 85 | | or/80-84 (955346) |
| 86 | | 61 and 85 (73) |
| 87 | | controlled clinical trial.pt. (88919) |
| 88 | | Controlled Clinical Trial/ or Controlled Clinical Trials as Topic/ (93885) |
| 89 | | (control* adj2 trial*).tw. (148259) |
| 90 | | (nonrandom* or non-random* or quasi-random* or quasi-experiment*).tw. (34429) |
| 91 | | (nRCT or nRCTs or non-RCT$1).tw. (287) |
| 92 | | (control* adj3 ("before and after" or "before after")).tw. (2721) |
| 93 | | time series.tw. (16273) |
| 94 | | (pre- adj3 post-).tw. (43648) |
| 95 | | (pretest adj3 posttest).tw. (3030) |
| 96 | | (control* adj2 stud$3).tw. (156907) |
| 97 | | Control Groups/ (1468) |
| 98 | | (control$ adj2 group$1).tw. (323397) |
| 99 | | or/87-98 (735918) |
| 100 | | 61 and 99 (109) |
| 101 | | exp Cohort Studies/ (1370879) |
| 102 | | cohort$1.tw. (286854) |
| 103 | | (longitudinal or prospective or retrospective).tw. (749021) |
| 104 | | ((followup or follow-up) adj (study or studies)).tw. (38587) |
| 105 | | exp Case-Control Studies/ (673186) |
| 106 | | ((case-control* or case-based or case-comparison) adj (study or studies)).tw. (67167) |
| 107 | | or/101-106 (1888261) |
| 108 | | 61 and 107 (139) |
| 109 | | 71 or 79 or 86 or 100 or 108 (304) |
| 110 | | (comment or editorial or interview or letter or news).pt. (1526324) |
| 111 | | 109 not 110 (295) |
| 112 | | Case Reports.pt. (1693418) |
| 113 | | (case adj (report$1 or study or studies or history or histories or series)).tw. (341362) |
| 114 | | Cross-Sectional Studies/ (179862) |
| 115 | | (cross-section* adj3 (study or studies or analy* or survey*)).tw. (126814) |
| 116 | | or/112-115 (2042337) |
| 117 | | 61 and 116 (125) |
| 118 | | 117 not 109 (108) |
| 119 | | 118 not 110 (104) |
|  | | |
| **Virus Type:**Filoviruses & Viral Hemorrhagic Fevers  **PPE:** Respirators, Foot and Scalp Protection  **Search Date:** August 7, 2014  **Study Design Types:** Systematic reviews, Meta-Analyses, Health Technology Assessments, Guidelines,  Protocols, Randomized Controlled Trials, Quasi-Experimental designs, Comparative  Cohort and Case-Control Studies, Single Arm Cohorts, Case-Reports and Cross-  Sectional Design | | |
| 1 | | expFiloviridae/ (1487) |
| 2 | | expFiloviridae Infections/ (1127) |
| 3 | | filovirida*.tw. (142) |
| 4 | | (Ebolavir* or Ebola vir* or Marburgvir* or Marburg vir* or Filovir* or Filo vir*).tw. (1801) |
| 5 | | (BDBV or EBOV or RESTV or SUDV or TAFV).tw. (303) |
| 6 | | Hemorrhagic Fever, Ebola/ (809) |
| 7 | | (ebola* adj10 (disease* or fever* or infect* or strain$1 or virus* or epidemic* or outbreak* or pandemic*)).tw. (1671) |
| 8 | | Marburg Virus Disease/ (360) |
| 9 | | (marburg* adj10 (disease* or fever* or infect* or strain$1 or virus* or epidemic* or outbreak* or pandemic*)).tw. (887) |
| 10 | | Hemorrhagic Fevers, Viral/ (1133) |
| 11 | | (h?emorrhagic fever$1 adj10 (virus* or viral*)).tw. (2592) |
| 12 | | Nairovirus/ (39) |
| 13 | | (nairovir* or nairovir* or dugbevir* or dugbevir*).tw. (173) |
| 14 | | Hemorrhagic Fever Virus, Crimean-Congo/ (508) |
| 15 | | Hemorrhagic Fever, Crimean/ (576) |
| 16 | | ((congo* or crimean*) adj10 (disease* or fever* or infect* or strain$1 or virus* or epidemic* or outbreak* or pandemic*)).tw. (2686) |
| 17 | | Arenaviridae Infections/ (480) |
| 18 | | (arenavir* or arena vir*).tw. (1039) |
| 19 | | Lassa Fever/ (457) |
| 20 | | Lassa virus/ (482) |
| 21 | | (lassa adj10 (disease* or fever* or infect* or strain$1 or virus* or epidemic* or outbreak* or pandemic*)).tw. (855) |
| 22 | | or/1-21 (8904) |
| 23 | | Shoes/ (4840) |
| 24 | | (shoe$1 or boot$1 or bootwear* or footwear* or shoewear*).tw. (9229) |
| 25 | | ((foot or feet) adj3 (apparel* or cover* or hygien* or precaution* or protect* or prophyla* or seal* or shield* or wear*)).tw. (669) |
| 26 | | Scalp/ (10169) |
| 27 | | ((head$1 or hair$1 or scalp$1) adj3 (apparel* or cover* or hygien* or precaution* or protect* or prophyla* or seal* or shield* or wear*)).tw. (2097) |
| 28 | | Respiratory Protective Devices/ (1645) |
| 29 | | (respirator or respirators or gasmask*).tw. (4241) |
| 30 | | (respiratory adj3 (device* or mask$1 or facecover* or face cover* or facialcover* or facial cover* or facemask* or facialmask* or facepiece* or face piece* or facialpiece* or facial piece* or faceseal* or face seal* or facialseal* or facial seal* or faceshield* or face shield* or facialshield* or facial shield*)).tw. (640) |
| 31 | | ((N95 or N-95) adj3 (device* or mask$1 or facecover* or face cover* or facialcover* or facial cover* or facemask* or facialmask* or facepiece* or face piece* or facialpiece* or facial piece* or faceseal* or face seal* or facialseal* or facial seal* or faceshield* or face shield* or facialshield* or facial shield*)).tw. (178) |
| 32 | | or/23-31 (29201) |
| 33 | | 22 and 32 (2) |
| 34 | | exp Animals/ not (exp Animals/ and Humans/) (3992529) |
| 35 | | 33 not 34 (2) |
| 36 | | limit 35 to systematic reviews (0) |
| 37 | | meta analysis.pt. (50768) |
| 38 | | exp meta-analysis as topic/ (13972) |
| 39 | | (meta-analy* or metanaly* or metaanaly* or met analy* or integrative research or integrative review* or integrative overview* or research integration or research overview* or collaborative review*).tw. (69760) |
| 40 | | (systematic review* or systematic overview* or evidence-based review* or evidence-based overview* or (evidence adj3 (review* or overview*)) or meta-review* or meta-overview* or meta-synthes* or "review of reviews" or technology assessment* or HTA or HTAs).tw. (87200) |
| 41 | | exp Technology assessment, biomedical/ (9259) |
| 42 | | (cochrane or health technology assessment or evidence report).jw. (12804) |
| 43 | | or/37-42 (165330) |
| 44 | | 35 and 43 (0) |
| 45 | | 36 or 44 (0) |
| 46 | | exp Guidelines as Topic/ (116095) |
| 47 | | exp Clinical Protocols/ (126595) |
| 48 | | Guideline.pt. (15629) |
| 49 | | Practice Guideline.pt. (19491) |
| 50 | | Consensus Development Conference.pt. (9270) |
| 51 | | (guidance* or guideline* or standards or recommendation*).ti. (102995) |
| 52 | | or/46-51 (325757) |
| 53 | | 35 and 52 (0) |
| 54 | | (controlled clinical trial or randomized controlled trial).pt. (470027) |
| 55 | | clinical trials as topic.sh. (171898) |
| 56 | | (randomi#ed or randomly or RCT$1 or placebo*).tw. (641308) |
| 57 | | ((singl* or doubl* or trebl* or tripl*) adj (mask* or blind* or dumm*)).tw. (133206) |
| 58 | | trial.ti. (131618) |
| 59 | | or/54-58 (965683) |
| 60 | | 35 and 59 (0) |
| 61 | | controlled clinical trial.pt. (89633) |
| 62 | | Controlled Clinical Trial/ or Controlled Clinical Trials as Topic/ (94627) |
| 63 | | (control* adj2 trial*).tw. (151046) |
| 64 | | (nonrandom* or non-random* or quasi-random* or quasi-experiment*).tw. (34979) |
| 65 | | (nRCT or nRCTs or non-RCT$1).tw. (295) |
| 66 | | (control* adj3 ("before and after" or "before after")).tw. (2749) |
| 67 | | time series.tw. (16382) |
| 68 | | (pre- adj3 post-).tw. (44147) |
| 69 | | (pretest adj3 posttest).tw. (3064) |
| 70 | | (control* adj2 stud$3).tw. (158885) |
| 71 | | Control Groups/ (1472) |
| 72 | | (control$ adj2 group$1).tw. (327030) |
| 73 | | or/61-72 (744583) |
| 74 | | 35 and 73 (0) |
| 75 | | exp Cohort Studies/ (1386410) |
| 76 | | cohort$1.tw. (291176) |
| 77 | | (longitudinal or prospective or retrospective).tw. (759183) |
| 78 | | ((followup or follow-up) adj (study or studies)).tw. (38935) |
| 79 | | exp Case-Control Studies/ (681415) |
| 80 | | ((case-control* or case-based or case-comparison) adj (study or studies)).tw. (67944) |
| 81 | | or/75-80 (1909307) |
| 82 | | 35 and 81 (0) |
| 83 | | Case Reports.pt. (1701068) |
| 84 | | (case adj (report$1 or study or studies or history or histories or series)).tw. (343815) |
| 85 | | Cross-Sectional Studies/ (182784) |
| 86 | | (cross-section* adj3 (study or studies or analy* or survey*)).tw. (129166) |
| 87 | | or/83-86 (2054593) |
| 88 | | 35 and 87 (1) |
| 89 | | 45 or 53 or 60 or 74 or 82 or 88 (1) |
| 90 | | (comment or editorial or interview or letter or news).pt. (1530005) |
| 91 | | 89 not 90 (1) |
|  | | |
| **EMBASE** | | |
| **Virus Type:** Filoviruses (Ebola, Marburg)  **PPE:** Gloves, Face protection and Gowns  **Search Date:** July 30, 2014  **Study Design Types:** Systematic reviews, Meta-Analyses, Health Technology Assessments, Guidelines,  Protocols, Randomized Controlled Trials, Quasi-Experimental designs, Comparative Cohort and  Case-Control Studies, Single Arm Cohorts. | | |
| 1 | | expfiloviridae/ (2550) |
| 2 | | expfilovirus infection/ (454) |
| 3 | | filovirida*.tw. (147) |
| 4 | | (Ebolavir* or Ebola vir* or Marburgvir* or Marburg vir* or Filovir* or Filo vir*).tw. (1901) |
| 5 | | (BDBV or EBOV or RESTV or SUDV or TAFV).tw. (293) |
| 6 | | (ebola* adj10 (disease* or fever* or infect* or strain$1 or virus* or epidemic* or outbreak* or pandemic*)).tw. (1770) |
| 7 | | (marburg* adj10 (disease* or fever* or infect* or strain$1 or virus* or epidemic* or outbreak* or pandemic*)).tw. (1046) |
| 8 | | or/1-7 (3401) |
| 9 | | exp infection control/ (81662) |
| 10 | | cross infection/pc [Prevention] (9917) |
| 11 | | epidemic/pc [Prevention] (6764) |
| 12 | | exp disease transmission/pc [Prevention] (3791) |
| 13 | | exp virus infection/pc [Prevention] (123409) |
| 14 | | ((diseas* or infect* or virus*) adj5 (prevent* or precaution* or control* or eliminat* or manag* or reduc* or stop*)).tw. (434319) |
| 15 | | ((epidemic* or outbreak* or pandemic*) adj5 (prevent* or precaution* or control* or eliminat* or manag* or reduc* or stop*)).tw. (13540) |
| 16 | | or/9-15 (600526) |
| 17 | | 8 and 16 (849) |
| 18 | | protective equipment/ (9597) |
| 19 | | protective clothing/ (10016) |
| 20 | | ((precaution* or protect* or prophyla*) adj3 (apparel* or attire* or barrier* or cloth* or device* or equipment* or garment* or gear or layer* or material* or workwear)).tw. (15792) |
| 21 | | PPE.tw. (2251) |
| 22 | | eye protective device/ (988) |
| 23 | | (eyecover* or eyeglasses or eyevisor* or eyewear* or glasses* or goggle$1 or (eye$1 adj2 visor*)).tw. (9868) |
| 24 | | mask/ (2358) |
| 25 | | exp face mask/ (4348) |
| 26 | | surgical mask/ (474) |
| 27 | | (mask$1 or facemask* or facialmask* or faceseal* or facialseal* or faceshield* or facialshield*).tw. (31871) |
| 28 | | ((eye or eyes or face or faces or facial* or ear or ears or mouth$1 or body or bodies or skin or hand or hands) adj3 (cover* or hygien* or precaution* or protect* or prophyla* or seal* or shield*)).tw. (22956) |
| 29 | | glove/ (4005) |
| 30 | | (glove$1 or gloving or single-glov* or double-glov* or triple-glov*).tw. (10466) |
| 31 | | exp surgical attire/ (3632) |
| 32 | | (apron* or gown or gowns or scrubs or theatre blues).tw. (2352) |
| 33 | | ((surgical or surger*) adj3 (apparel* or attire* or cloth* or garment* or workwear)).tw. (126) |
| 34 | | occupational exposure/pc [Prevention] (2665) |
| 35 | | (occupation* adj3 (expos* or contaminat* or infect* or transmission* or transmit*) adj5 (prevent* or precaution* or control* or eliminat* or manag* or reduc* or stop*)).tw. (1783) |
| 36 | | ((personnel* or HCW or HCWs or worker* or (health* adj3 professional*) or clinician* or doctor* or nurse* or physician* or practitioner*) adj3 (expos* or contaminat* or infect* or transmission* or transmit*) adj5 (prevent* or precaution* or control* or eliminat* or manag* or reduc* or stop*)).tw. (4027) |
| 37 | | (occupational adj3 (hygiene or safety)).tw. (7629) |
| 38 | | ((safety or standard* or universal) adj3 precaution*).tw. (2934) |
| 39 | | equipment contamination/pc [Prevention] (83) |
| 40 | | (contaminat* adj5 (prevent* or precaution* or control* or eliminat* or manag* or reduc* or stop*)).tw. (13992) |
| 41 | | or/18-40 (135502) |
| 42 | | 8 and 41 (73) |
| 43 | | 17 or 42 (872) |
| 44 | | exp animal experimentation/ or exp models animal/ or exp animal experiment/ or nonhuman/ or exp vertebrate/ (20714718) |
| 45 | | exp humans/ or exp human experimentation/ or exp human experiment/ (15136790) |
| 46 | | 44 not 45 (5578940) |
| 47 | | 43 not 46 (687) |
| 48 | | exp meta-analysis as topic/ (14465) |
| 49 | | (meta-analy* or metanaly* or metaanaly* or met analy* or integrative research or integrative review* or integrative overview* or research integration or research overview* or collaborative review*).tw. (85502) |
| 50 | | (systematic review* or systematic overview* or evidence-based review* or evidence-based overview* or (evidence adj3 (review* or overview*)) or meta-review* or meta-overview* or meta-synthes* or "review of reviews" or technology assessment* or HTA or HTAs).tw. (101128) |
| 51 | | exp Technology assessment, biomedical/ (11384) |
| 52 | | (cochrane or health technology assessment or evidence report).jw. (12623) |
| 53 | | or/48-52 (188713) |
| 54 | | 47 and 53 (4) |
| 55 | | limit 47 to (meta analysis or "systematic review") (1) |
| 56 | | 54 or 55 (5) |
| 57 | | exp practice guideline/ (316164) |
| 58 | | (guideline* or standards or recommendation*).ti. (115558) |
| 59 | | (expert consensus or consensus statement* or consensus conference* or practice parameter* or position statement* or policy statement* or CPG or CPGs).tw. (42244) |
| 60 | | or/57-59 (412787) |
| 61 | | 47 and 60 (29) |
| 62 | | randomized controlled trial/ or controlled clinical trial/ (483022) |
| 63 | | exp "clinical trial (topic)"/ (110903) |
| 64 | | (randomi#ed or randomly or RCT$1 or placebo*).tw. (815927) |
| 65 | | ((singl* or doubl* or trebl* or tripl*) adj (mask* or blind* or dumm*)).tw. (168863) |
| 66 | | trial.ti. (169283) |
| 67 | | or/62-66 (1156433) |
| 68 | | 47 and 67 (15) |
| 69 | | controlled clinical trial/ (386257) |
| 70 | | "controlled clinical trial (topic)"/ (3043) |
| 71 | | (control* adj2 trial*).tw. (183027) |
| 72 | | (nonrandom* or non-random* or quasi-random* or quasi-experiment*).tw. (40468) |
| 73 | | (nRCT or nRCTs or non-RCT$1).tw. (390) |
| 74 | | (control* adj3 ("before and after" or "before after")).tw. (3419) |
| 75 | | time series analysis/ (14223) |
| 76 | | time series.tw. (18324) |
| 77 | | pretest posttest control group design/ (203) |
| 78 | | (pre- adj3 post-).tw. (69822) |
| 79 | | (pretest adj3 posttest).tw. (3347) |
| 80 | | controlled study/ (4382229) |
| 81 | | (control* adj2 stud$3).tw. (195456) |
| 82 | | control group/ (75547) |
| 83 | | (control$ adj2 group$1).tw. (441322) |
| 84 | | trial.ti. (169283) |
| 85 | | or/69-84 (4921749) |
| 86 | | 47 and 85 (82) |
| 87 | | cohort analysis/ (173485) |
| 88 | | cohort$1.tw. (408355) |
| 89 | | retrospective study/ (354313) |
| 90 | | longitudinal study/ (68961) |
| 91 | | prospective study/ (258015) |
| 92 | | (longitudinal or prospective or retrospective).tw. (1008397) |
| 93 | | follow up/ (842704) |
| 94 | | ((followup or follow-up) adj (study or studies)).tw. (51557) |
| 95 | | observational study/ (58555) |
| 96 | | (observation$2 adj (study or studies)).tw. (66834) |
| 97 | | population research/ (69573) |
| 98 | | ((population or population-based) adj (study or studies or analys#s)).tw. (14861) |
| 99 | | ((multidimensional or multi-dimensional) adj (study or studies)).tw. (110) |
| 100 | | exp comparative study/ (1025275) |
| 101 | | ((comparative or comparison) adj (study or studies)).tw. (107065) |
| 102 | | exp case control study/ (86948) |
| 103 | | ((case-control* or case-based or case-comparison) adj (study or studies)).tw. (78329) |
| 104 | | or/87-103 (3197633) |
| 105 | | 47 and 104 (33) |
| 106 | | 56 or 61 or 68 or 86 or 105 (151) |
| 107 | | (editorial or letter).pt. (1304084) |
| 108 | | 106 not 107 (148) |
|  | | |
| **Virus Type:** Filoviruses (Ebola, Marburg)  **PPE:** Gloves, Face protection and Gowns  **Search Date:** July 31, 2014  **Study Design Types:** Case-Reports and Cross-Sectional Design | | |
| 1 | | exp filoviridae/ (2583) |
| 2 | | exp filovirus infection/ (483) |
| 3 | | filovirida*.tw. (149) |
| 4 | | (Ebolavir* or Ebola vir* or Marburgvir* or Marburg vir* or Filovir* or Filo vir*).tw. (1924) |
| 5 | | (BDBV or EBOV or RESTV or SUDV or TAFV).tw. (296) |
| 6 | | (ebola* adj10 (disease* or fever* or infect* or strain$1 or virus* or epidemic* or outbreak* or pandemic*)).tw. (1793) |
| 7 | | (marburg* adj10 (disease* or fever* or infect* or strain$1 or virus* or epidemic* or outbreak* or pandemic*)).tw. (1048) |
| 8 | | or/1-7 (3448) |
| 9 | | exp infection control/ (82181) |
| 10 | | cross infection/pc [Prevention] (9936) |
| 11 | | epidemic/pc [Prevention] (6795) |
| 12 | | exp disease transmission/pc [Prevention] (3803) |
| 13 | | exp virus infection/pc [Prevention] (123988) |
| 14 | | ((diseas* or infect* or virus*) adj5 (prevent* or precaution* or control* or eliminat* or manag* or reduc* or stop*)).tw. (438833) |
| 15 | | ((epidemic* or outbreak* or pandemic*) adj5 (prevent* or precaution* or control* or eliminat* or manag* or reduc* or stop*)).tw. (13664) |
| 16 | | or/9-15 (605874) |
| 17 | | 8 and 16 (859) |
| 18 | | protective equipment/ (9661) |
| 19 | | protective clothing/ (10054) |
| 20 | | ((precaution* or protect* or prophyla*) adj3 (apparel* or attire* or barrier* or cloth* or device* or equipment* or garment* or gear or layer* or material* or workwear)).tw. (15947) |
| 21 | | PPE.tw. (2276) |
| 22 | | eye protective device/ (991) |
| 23 | | (eyecover* or eyeglasses or eyevisor* or eyewear* or glasses* or goggle$1 or (eye$1 adj2 visor*)).tw. (9954) |
| 24 | | mask/ (2369) |
| 25 | | exp face mask/ (4386) |
| 26 | | surgical mask/ (482) |
| 27 | | (mask$1 or facemask* or facialmask* or faceseal* or facialseal* or faceshield* or facialshield*).tw. (32118) |
| 28 | | ((eye or eyes or face or faces or facial* or ear or ears or mouth$1 or body or bodies or skin or hand or hands) adj3 (cover* or hygien* or precaution* or protect* or prophyla* or seal* or shield*)).tw. (23167) |
| 29 | | glove/ (4045) |
| 30 | | (glove$1 or gloving or single-glov* or double-glov* or triple-glov*).tw. (10543) |
| 31 | | exp surgical attire/ (3664) |
| 32 | | (apron* or gown or gowns or scrubs or theatre blues).tw. (2376) |
| 33 | | ((surgical or surger*) adj3 (apparel* or attire* or cloth* or garment* or workwear)).tw. (129) |
| 34 | | occupational exposure/pc [Prevention] (2675) |
| 35 | | (occupation* adj3 (expos* or contaminat* or infect* or transmission* or transmit*) adj5 (prevent* or precaution* or control* or eliminat* or manag* or reduc* or stop*)).tw. (1795) |
| 36 | | ((personnel* or HCW or HCWs or worker* or (health* adj3 professional*) or clinician* or doctor* or nurse* or physician* or practitioner*) adj3 (expos* or contaminat* or infect* or transmission* or transmit*) adj5 (prevent* or precaution* or control* or eliminat* or manag* or reduc* or stop*)).tw. (4052) |
| 37 | | (occupational adj3 (hygiene or safety)).tw. (7682) |
| 38 | | ((safety or standard* or universal) adj3 precaution*).tw. (2951) |
| 39 | | equipment contamination/pc [Prevention] (91) |
| 40 | | (contaminat* adj5 (prevent* or precaution* or control* or eliminat* or manag* or reduc* or stop*)).tw. (14110) |
| 41 | | or/18-40 (136572) |
| 42 | | 8 and 41 (75) |
| 43 | | 17 or 42 (884) |
| 44 | | exp animal experimentation/ or exp models animal/ or exp animal experiment/ or nonhuman/ or exp vertebrate/ (20860433) |
| 45 | | exp humans/ or exp human experimentation/ or exp human experiment/ (15258179) |
| 46 | | 44 not 45 (5603266) |
| 47 | | 43 not 46 (699) |
| 48 | | exp meta-analysis as topic/ (15226) |
| 49 | | (meta-analy* or metanaly* or metaanaly* or met analy* or integrative research or integrative review* or integrative overview* or research integration or research overview* or collaborative review*).tw. (87412) |
| 50 | | (systematic review* or systematic overview* or evidence-based review* or evidence-based overview* or (evidence adj3 (review* or overview*)) or meta-review* or meta-overview* or meta-synthes* or "review of reviews" or technology assessment* or HTA or HTAs).tw. (103089) |
| 51 | | exp Technology assessment, biomedical/ (11397) |
| 52 | | (cochrane or health technology assessment or evidence report).jw. (12640) |
| 53 | | or/48-52 (192379) |
| 54 | | 47 and 53 (4) |
| 55 | | limit 47 to (meta analysis or "systematic review") (1) |
| 56 | | 54 or 55 (5) |
| 57 | | exp practice guideline/ (318956) |
| 58 | | (guideline* or standards or recommendation*).ti. (116574) |
| 59 | | (expert consensus or consensus statement* or consensus conference* or practice parameter* or position statement* or policy statement* or CPG or CPGs).tw. (42804) |
| 60 | | or/57-59 (416558) |
| 61 | | 47 and 60 (29) |
| 62 | | randomized controlled trial/ or controlled clinical trial/ (486395) |
| 63 | | exp "clinical trial (topic)"/ (115588) |
| 64 | | (randomi#ed or randomly or RCT$1 or placebo*).tw. (825950) |
| 65 | | ((singl* or doubl* or trebl* or tripl*) adj (mask* or blind* or dumm*)).tw. (170396) |
| 66 | | trial.ti. (171432) |
| 67 | | or/62-66 (1170799) |
| 68 | | 47 and 67 (15) |
| 69 | | controlled clinical trial/ (387077) |
| 70 | | "controlled clinical trial (topic)"/ (3208) |
| 71 | | (control* adj2 trial*).tw. (185737) |
| 72 | | (nonrandom* or non-random* or quasi-random* or quasi-experiment*).tw. (40908) |
| 73 | | (nRCT or nRCTs or non-RCT$1).tw. (402) |
| 74 | | (control* adj3 ("before and after" or "before after")).tw. (3451) |
| 75 | | time series analysis/ (14402) |
| 76 | | time series.tw. (18562) |
| 77 | | pretest posttest control group design/ (206) |
| 78 | | (pre- adj3 post-).tw. (71132) |
| 79 | | (pretest adj3 posttest).tw. (3383) |
| 80 | | controlled study/ (4416471) |
| 81 | | (control* adj2 stud$3).tw. (197636) |
| 82 | | control group/ (77657) |
| 83 | | (control$ adj2 group$1).tw. (446609) |
| 84 | | trial.ti. (171432) |
| 85 | | or/69-84 (4963379) |
| 86 | | 47 and 85 (82) |
| 87 | | cohort analysis/ (177126) |
| 88 | | cohort$1.tw. (416993) |
| 89 | | retrospective study/ (359734) |
| 90 | | longitudinal study/ (69916) |
| 91 | | prospective study/ (262123) |
| 92 | | (longitudinal or prospective or retrospective).tw. (1023748) |
| 93 | | follow up/ (856652) |
| 94 | | ((followup or follow-up) adj (study or studies)).tw. (51916) |
| 95 | | observational study/ (60502) |
| 96 | | (observation$2 adj (study or studies)).tw. (68350) |
| 97 | | population research/ (70139) |
| 98 | | ((population or population-based) adj (study or studies or analys#s)).tw. (15017) |
| 99 | | ((multidimensional or multi-dimensional) adj (study or studies)).tw. (112) |
| 100 | | exp comparative study/ (1030986) |
| 101 | | ((comparative or comparison) adj (study or studies)).tw. (107772) |
| 102 | | exp case control study/ (88196) |
| 103 | | ((case-control* or case-based or case-comparison) adj (study or studies)).tw. (79355) |
| 104 | | or/87-103 (3235731) |
| 105 | | 47 and 104 (35) |
| 106 | | 56 or 61 or 68 or 86 or 105 (153) |
| 107 | | (editorial or letter).pt. (1311499) |
| 108 | | 106 not 107 (150) |
| 109 | | case report/ (1975737) |
| 110 | | case study/ (36934) |
| 111 | | (case adj (report$1 or study or studies or history or histories or series)).tw. (476374) |
| 112 | | cross-sectional study/ (121131) |
| 113 | | (cross-section* adj3 (study or studies or analy* or survey*)).tw. (155348) |
| 114 | | or/109-113 (2342790) |
| 115 | | 47 and 114 (20) |
| 116 | | 115 not 106 (15) |
| 117 | | 116 not 107 (15) |
|  | | |
| **Virus Type:**Filoviruses & Viral Hemorrhagic Fevers  **PPE:** Gloves, Face Protection and Gowns  **Search Date:** July 30, 2014  **Study Design Types:** Systematic reviews, Meta-Analyses, Health Technology Assessments, Guidelines,  Protocols, Randomized Controlled Trials, Quasi-Experimental designs, Comparative  Cohort and Case-Control Studies, Single Arm Cohorts. | | |
| 1 | | expfiloviridae/ (2550) |
| 2 | | expfilovirus infection/ (454) |
| 3 | | filovirida*.tw. (147) |
| 4 | | (Ebolavir* or Ebola vir* or Marburgvir* or Marburg vir* or Filovir* or Filo vir*).tw. (1901) |
| 5 | | (BDBV or EBOV or RESTV or SUDV or TAFV).tw. (293) |
| 6 | | (ebola* adj10 (disease* or fever* or infect* or strain$1 or virus* or epidemic* or outbreak* or pandemic*)).tw. (1770) |
| 7 | | (marburg* adj10 (disease* or fever* or infect* or strain$1 or virus* or epidemic* or outbreak* or pandemic*)).tw. (1046) |
| 8 | | Hemorrhagic Fever, Ebola/ (342) |
| 9 | | (h?emorrhagic fever$1 adj10 (virus* or viral*)).tw. (3042) |
| 10 | | Nairo virus/ (655) |
| 11 | | Nairovirus infection/ (4) |
| 12 | | (nairovir* or nairovir* or dugbevir* or dugbevir*).tw. (216) |
| 13 | | Crimean Congo hemorrhagic fever/ (540) |
| 14 | | ((congo* or crimean*) adj10 (disease* or fever* or infect* or strain$1 or virus* or epidemic* or outbreak* or pandemic*)).tw. (3347) |
| 15 | | arenavirus infection/ (114) |
| 16 | | (arenavir* or arena vir*).tw. (1104) |
| 17 | | Lassa fever/ (635) |
| 18 | | Lassa virus/ (696) |
| 19 | | (lassa adj10 (disease* or fever* or infect* or strain$1 or virus* or epidemic* or outbreak* or pandemic*)).tw. (947) |
| 20 | | Cholera/ (10881) |
| 21 | | Vibrio cholera/ (11300) |
| 22 | | (cholera* or choleric*).tw. (29259) |
| 23 | | or/1-22 (43736) |
| 24 | | exp infection control/ (81662) |
| 25 | | cross infection/pc [Prevention] (9917) |
| 26 | | epidemic/pc [Prevention] (6764) |
| 27 | | exp disease transmission/pc [Prevention] (3791) |
| 28 | | exp virus infection/pc [Prevention] (123409) |
| 29 | | ((diseas* or infect* or virus*) adj5 (prevent* or precaution* or control* or eliminat* or manag* or reduc* or stop*)).tw. (434319) |
| 30 | | ((epidemic* or outbreak* or pandemic*) adj5 (prevent* or precaution* or control* or eliminat* or manag* or reduc* or stop*)).tw. (13540) |
| 31 | | or/24-30 (600526) |
| 32 | | 23 and 31 (4393) |
| 33 | | protective equipment/ (9597) |
| 34 | | protective clothing/ (10016) |
| 35 | | ((precaution* or protect* or prophyla*) adj3 (apparel* or attire* or barrier* or cloth* or device* or equipment* or garment* or gear or layer* or material* or workwear)).tw. (15792) |
| 36 | | PPE.tw. (2251) |
| 37 | | eye protective device/ (988) |
| 38 | | (eyecover* or eyeglasses or eyevisor* or eyewear* or glasses* or goggle$1 or (eye$1 adj2 visor*)).tw. (9868) |
| 39 | | mask/ (2358) |
| 40 | | exp face mask/ (4348) |
| 41 | | surgical mask/ (474) |
| 42 | | (mask$1 or facemask* or facialmask* or faceseal* or facialseal* or faceshield* or facialshield*).tw. (31871) |
| 43 | | ((eye or eyes or face or faces or facial* or ear or ears or mouth$1 or body or bodies or skin or hand or hands) adj3 (cover* or hygien* or precaution* or protect* or prophyla* or seal* or shield*)).tw. (22956) |
| 44 | | glove/ (4005) |
| 45 | | (glove$1 or gloving or single-glov* or double-glov* or triple-glov*).tw. (10466) |
| 46 | | exp surgical attire/ (3632) |
| 47 | | (apron* or gown or gowns or scrubs or theatre blues).tw. (2352) |
| 48 | | ((surgical or surger*) adj3 (apparel* or attire* or cloth* or garment* or workwear)).tw. (126) |
| 49 | | occupational exposure/pc [Prevention] (2665) |
| 50 | | (occupation* adj3 (expos* or contaminat* or infect* or transmission* or transmit*) adj5 (prevent* or precaution* or control* or eliminat* or manag* or reduc* or stop*)).tw. (1783) |
| 51 | | ((personnel* or HCW or HCWs or worker* or (health* adj3 professional*) or clinician* or doctor* or nurse* or physician* or practitioner*) adj3 (expos* or contaminat* or infect* or transmission* or transmit*) adj5 (prevent* or precaution* or control* or eliminat* or manag* or reduc* or stop*)).tw. (4027) |
| 52 | | (occupational adj3 (hygiene or safety)).tw. (7629) |
| 53 | | ((safety or standard* or universal) adj3 precaution*).tw. (2934) |
| 54 | | equipment contamination/pc [Prevention] (83) |
| 55 | | (contaminat* adj5 (prevent* or precaution* or control* or eliminat* or manag* or reduc* or stop*)).tw. (13992) |
| 56 | | or/33-55 (135502) |
| 57 | | 23 and 56 (274) |
| 58 | | 32 or 57 (4544) |
| 59 | | exp animal experimentation/ or exp models animal/ or exp animal experiment/ or nonhuman/ or exp vertebrate/ (20714718) |
| 60 | | exp humans/ or exp human experimentation/ or exp human experiment/ (15136790) |
| 61 | | 59 not 60 (5578940) |
| 62 | | 58 not 61 (3457) |
| 63 | | exp meta-analysis as topic/ (14465) |
| 64 | | (meta-analy* or metanaly* or metaanaly* or met analy* or integrative research or integrative review* or integrative overview* or research integration or research overview* or collaborative review*).tw. (85502) |
| 65 | | (systematic review* or systematic overview* or evidence-based review* or evidence-based overview* or (evidence adj3 (review* or overview*)) or meta-review* or meta-overview* or meta-synthes* or "review of reviews" or technology assessment* or HTA or HTAs).tw. (101128) |
| 66 | | exp Technology assessment, biomedical/ (11384) |
| 67 | | (cochrane or health technology assessment or evidence report).jw. (12623) |
| 68 | | or/63-67 (188713) |
| 69 | | 62 and 68 (22) |
| 70 | | limit 62 to (meta analysis or "systematic review") (16) |
| 71 | | 69 or 70 (28) |
| 72 | | exp practice guideline/ (316164) |
| 73 | | (guidance* or guideline* or standards or recommendation*).ti. (128894) |
| 74 | | (expert consensus or consensus statement* or consensus conference* or practice parameter* or position statement* or policy statement* or CPG or CPGs).tw. (42244) |
| 75 | | or/72-74 (423890) |
| 76 | | 62 and 75 (168) |
| 77 | | randomized controlled trial/ or controlled clinical trial/ (483022) |
| 78 | | exp "clinical trial (topic)"/ (110903) |
| 79 | | (randomi#ed or randomly or RCT$1 or placebo*).tw. (815927) |
| 80 | | ((singl* or doubl* or trebl* or tripl*) adj (mask* or blind* or dumm*)).tw. (168863) |
| 81 | | trial.ti. (169283) |
| 82 | | or/77-81 (1156433) |
| 83 | | 62 and 82 (100) |
| 84 | | controlled clinical trial/ (386257) |
| 85 | | "controlled clinical trial (topic)"/ (3043) |
| 86 | | (control* adj2 trial*).tw. (183027) |
| 87 | | (nonrandom* or non-random* or quasi-random* or quasi-experiment*).tw. (40468) |
| 88 | | (nRCT or nRCTs or non-RCT$1).tw. (390) |
| 89 | | (control* adj3 ("before and after" or "before after")).tw. (3419) |
| 90 | | time series analysis/ (14223) |
| 91 | | time series.tw. (18324) |
| 92 | | pretest posttest control group design/ (203) |
| 93 | | (pre- adj3 post-).tw. (69822) |
| 94 | | (pretest adj3 posttest).tw. (3347) |
| 95 | | controlled study/ (4382229) |
| 96 | | (control* adj2 stud$3).tw. (195456) |
| 97 | | control group/ (75547) |
| 98 | | (control$ adj2 group$1).tw. (441322) |
| 99 | | trial.ti. (169283) |
| 100 | | or/84-99 (4921749) |
| 101 | | 62 and 100 (414) |
| 102 | | cohort analysis/ (173485) |
| 103 | | cohort$1.tw. (408355) |
| 104 | | retrospective study/ (354313) |
| 105 | | longitudinal study/ (68961) |
| 106 | | prospective study/ (258015) |
| 107 | | (longitudinal or prospective or retrospective).tw. (1008397) |
| 108 | | follow up/ (842704) |
| 109 | | ((followup or follow-up) adj (study or studies)).tw. (51557) |
| 110 | | observational study/ (58555) |
| 111 | | (observation$2 adj (study or studies)).tw. (66834) |
| 112 | | population research/ (69573) |
| 113 | | ((population or population-based) adj (study or studies or analys#s)).tw. (14861) |
| 114 | | ((multidimensional or multi-dimensional) adj (study or studies)).tw. (110) |
| 115 | | exp comparative study/ (1025275) |
| 116 | | ((comparative or comparison) adj (study or studies)).tw. (107065) |
| 117 | | exp case control study/ (86948) |
| 118 | | ((case-control* or case-based or case-comparison) adj (study or studies)).tw. (78329) |
| 119 | | or/102-118 (3197633) |
| 120 | | 62 and 119 (254) |
| 121 | | 71 or 76 or 83 or 101 or 120 (769) |
| 122 | | (editorial or letter).pt. (1304084) |
| 123 | | 121 not 122 (755) |
|  | | |
| **Virus Type:**Filoviruses & Viral Hemorrhagic Fevers  **PPE:** Gloves, Face Protection and Gowns  **Search Date:** July 31, 2014  **Study Design Types:** Case-Reports and Cross-Sectional Design | | |
| 1 | | expfiloviridae/ (2551) |
| 2 | | expfilovirus infection/ (456) |
| 3 | | filovirida*.tw. (147) |
| 4 | | (Ebolavir* or Ebola vir* or Marburgvir* or Marburg vir* or Filovir* or Filo vir*).tw. (1902) |
| 5 | | (BDBV or EBOV or RESTV or SUDV or TAFV).tw. (293) |
| 6 | | (ebola* adj10 (disease* or fever* or infect* or strain$1 or virus* or epidemic* or outbreak* or pandemic*)).tw. (1770) |
| 7 | | (marburg* adj10 (disease* or fever* or infect* or strain$1 or virus* or epidemic* or outbreak* or pandemic*)).tw. (1046) |
| 8 | | Hemorrhagic Fever, Ebola/ (343) |
| 9 | | (h?emorrhagic fever$1 adj10 (virus* or viral*)).tw. (3044) |
| 10 | | Nairo virus/ (656) |
| 11 | | Nairovirus infection/ (4) |
| 12 | | (nairovir* or nairovir* or dugbevir* or dugbevir*).tw. (217) |
| 13 | | Crimean Congo hemorrhagic fever/ (540) |
| 14 | | ((congo* or crimean*) adj10 (disease* or fever* or infect* or strain$1 or virus* or epidemic* or outbreak* or pandemic*)).tw. (3349) |
| 15 | | arenavirus infection/ (114) |
| 16 | | (arenavir* or arena vir*).tw. (1104) |
| 17 | | Lassa fever/ (635) |
| 18 | | Lassa virus/ (696) |
| 19 | | (lassa adj10 (disease* or fever* or infect* or strain$1 or virus* or epidemic* or outbreak* or pandemic*)).tw. (947) |
| 20 | | Cholera/ (10884) |
| 21 | | Vibrio cholera/ (11303) |
| 22 | | (cholera* or choleric*).tw. (29264) |
| 23 | | or/1-22 (43748) |
| 24 | | exp infection control/ (81684) |
| 25 | | cross infection/pc [Prevention] (9918) |
| 26 | | epidemic/pc [Prevention] (6765) |
| 27 | | exp disease transmission/pc [Prevention] (3792) |
| 28 | | exp virus infection/pc [Prevention] (123452) |
| 29 | | ((diseas* or infect* or virus*) adj5 (prevent* or precaution* or control* or eliminat* or manag* or reduc* or stop*)).tw. (434497) |
| 30 | | ((epidemic* or outbreak* or pandemic*) adj5 (prevent* or precaution* or control* or eliminat* or manag* or reduc* or stop*)).tw. (13547) |
| 31 | | or/24-30 (600759) |
| 32 | | 23 and 31 (4395) |
| 33 | | protective equipment/ (9599) |
| 34 | | protective clothing/ (10018) |
| 35 | | ((precaution* or protect* or prophyla*) adj3 (apparel* or attire* or barrier* or cloth* or device* or equipment* or garment* or gear or layer* or material* or workwear)).tw. (15798) |
| 36 | | PPE.tw. (2251) |
| 37 | | eye protective device/ (988) |
| 38 | | (eyecover* or eyeglasses or eyevisor* or eyewear* or glasses* or goggle$1 or (eye$1 adj2 visor*)).tw. (9873) |
| 39 | | mask/ (2358) |
| 40 | | exp face mask/ (4350) |
| 41 | | surgical mask/ (474) |
| 42 | | (mask$1 or facemask* or facialmask* or faceseal* or facialseal* or faceshield* or facialshield*).tw. (31880) |
| 43 | | ((eye or eyes or face or faces or facial* or ear or ears or mouth$1 or body or bodies or skin or hand or hands) adj3 (cover* or hygien* or precaution* or protect* or prophyla* or seal* or shield*)).tw. (22963) |
| 44 | | glove/ (4005) |
| 45 | | (glove$1 or gloving or single-glov* or double-glov* or triple-glov*).tw. (10469) |
| 46 | | exp surgical attire/ (3633) |
| 47 | | (apron* or gown or gowns or scrubs or theatre blues).tw. (2353) |
| 48 | | ((surgical or surger*) adj3 (apparel* or attire* or cloth* or garment* or workwear)).tw. (126) |
| 49 | | occupational exposure/pc [Prevention] (2667) |
| 50 | | (occupation* adj3 (expos* or contaminat* or infect* or transmission* or transmit*) adj5 (prevent* or precaution* or control* or eliminat* or manag* or reduc* or stop*)).tw. (1783) |
| 51 | | ((personnel* or HCW or HCWs or worker* or (health* adj3 professional*) or clinician* or doctor* or nurse* or physician* or practitioner*) adj3 (expos* or contaminat* or infect* or transmission* or transmit*) adj5 (prevent* or precaution* or control* or eliminat* or manag* or reduc* or stop*)).tw. (4029) |
| 52 | | (occupational adj3 (hygiene or safety)).tw. (7629) |
| 53 | | ((safety or standard* or universal) adj3 precaution*).tw. (2936) |
| 54 | | equipment contamination/pc [Prevention] (83) |
| 55 | | (contaminat* adj5 (prevent* or precaution* or control* or eliminat* or manag* or reduc* or stop*)).tw. (13995) |
| 56 | | or/33-55 (135541) |
| 57 | | 23 and 56 (274) |
| 58 | | 32 or 57 (4546) |
| 59 | | exp animal experimentation/ or exp models animal/ or exp animal experiment/ or nonhuman/ or exp vertebrate/ (20719757) |
| 60 | | exp humans/ or exp human experimentation/ or exp human experiment/ (15140856) |
| 61 | | 59 not 60 (5579913) |
| 62 | | 58 not 61 (3459) |
| 63 | | exp meta-analysis as topic/ (14489) |
| 64 | | (meta-analy* or metanaly* or metaanaly* or met analy* or integrative research or integrative review* or integrative overview* or research integration or research overview* or collaborative review*).tw. (85568) |
| 65 | | (systematic review* or systematic overview* or evidence-based review* or evidence-based overview* or (evidence adj3 (review* or overview*)) or meta-review* or meta-overview* or meta-synthes* or "review of reviews" or technology assessment* or HTA or HTAs).tw. (101199) |
| 66 | | exp Technology assessment, biomedical/ (11385) |
| 67 | | (cochrane or health technology assessment or evidence report).jw. (12623) |
| 68 | | or/63-67 (188840) |
| 69 | | 62 and 68 (22) |
| 70 | | limit 62 to (meta analysis or "systematic review") (16) |
| 71 | | 69 or 70 (28) |
| 72 | | exp practice guideline/ (316299) |
| 73 | | (guidance* or guideline* or standards or recommendation*).ti. (128931) |
| 74 | | (expert consensus or consensus statement* or consensus conference* or practice parameter* or position statement* or policy statement* or CPG or CPGs).tw. (42259) |
| 75 | | or/72-74 (424045) |
| 76 | | 62 and 75 (168) |
| 77 | | randomized controlled trial/ or controlled clinical trial/ (483125) |
| 78 | | exp "clinical trial (topic)"/ (111041) |
| 79 | | (randomi#ed or randomly or RCT$1 or placebo*).tw. (816214) |
| 80 | | ((singl* or doubl* or trebl* or tripl*) adj (mask* or blind* or dumm*)).tw. (168901) |
| 81 | | trial.ti. (169335) |
| 82 | | or/77-81 (1156841) |
| 83 | | 62 and 82 (100) |
| 84 | | controlled clinical trial/ (386283) |
| 85 | | "controlled clinical trial (topic)"/ (3050) |
| 86 | | (control* adj2 trial*).tw. (183104) |
| 87 | | (nonrandom* or non-random* or quasi-random* or quasi-experiment*).tw. (40485) |
| 88 | | (nRCT or nRCTs or non-RCT$1).tw. (390) |
| 89 | | (control* adj3 ("before and after" or "before after")).tw. (3420) |
| 90 | | time series analysis/ (14229) |
| 91 | | time series.tw. (18332) |
| 92 | | pretest posttest control group design/ (204) |
| 93 | | (pre- adj3 post-).tw. (69855) |
| 94 | | (pretest adj3 posttest).tw. (3349) |
| 95 | | controlled study/ (4383574) |
| 96 | | (control* adj2 stud$3).tw. (195505) |
| 97 | | control group/ (75560) |
| 98 | | (control$ adj2 group$1).tw. (441470) |
| 99 | | trial.ti. (169335) |
| 100 | | or/84-99 (4923254) |
| 101 | | 62 and 100 (414) |
| 102 | | cohort analysis/ (173606) |
| 103 | | cohort$1.tw. (408568) |
| 104 | | retrospective study/ (354572) |
| 105 | | longitudinal study/ (68993) |
| 106 | | prospective study/ (258158) |
| 107 | | (longitudinal or prospective or retrospective).tw. (1008871) |
| 108 | | follow up/ (843048) |
| 109 | | ((followup or follow-up) adj (study or studies)).tw. (515564) |
| 110 | | observational study/ (58604) |
| 111 | | (observation$2 adj (study or studies)).tw. (66876) |
| 112 | | population research/ (69588) |
| 113 | | ((population or population-based) adj (study or studies or analys#s)).tw. (14863) |
| 114 | | ((multidimensional or multi-dimensional) adj (study or studies)).tw. (110) |
| 115 | | exp comparative study/ (102558) |
| 116 | | ((comparative or comparison) adj (study or studies)).tw. (107087) |
| 117 | | exp case control study/ (86993) |
| 118 | | ((case-control* or case-based or case-comparison) adj (study or studies)).tw. (78354) |
| 119 | | or/102-118 (3198853) |
| 120 | | 62 and 119 (254) |
| 121 | | 71 or 76 or 83 or 101 or 120 (769) |
| 122 | | (editorial or letter).pt. (1304380) |
| 123 | | 121 not 122 (752) |
| 124 | | case report/ (1966670) |
| 125 | | case study/ (36209) |
| 126 | | (case adj (report$1 or study or studies or history or histories or series)).tw. (471626) |
| 127 | | cross-sectional study/ (118309) |
| 128 | | (cross-section* adj3 (study or studies or analy* or survey*)).tw. (152630) |
| 129 | | or/124-128 (2328709) |
| 130 | | 62 and 129 (118) |
| 131 | | 130 not 121 (94) |
| 132 | | 131 not 122 (91) |
|  | | |
| **Virus Type:**Filoviruses & Viral Hemorrhagic Fevers  **PPE:** Respirators, Foot and Scalp Protection  **Search Date:** August 7, 2014  **Study Design Types:** Systematic reviews, Meta-Analyses, Health Technology Assessments, Guidelines,  Protocols, Randomized Controlled Trials, Quasi-Experimental designs, Comparative  Cohort and Case-Control Studies, Single Arm Cohorts, Case-Reports and Cross-  Sectional Design | | |
| 1 | | expfiloviridae/ (2555) |
| 2 | | expfilovirus infection/ (463) |
| 3 | | filovirida*.tw. (147) |
| 4 | | (Ebolavir* or Ebola vir* or Marburgvir* or Marburg vir* or Filovir* or Filo vir*).tw. (1905) |
| 5 | | (BDBV or EBOV or RESTV or SUDV or TAFV).tw. (293) |
| 6 | | (ebola* adj10 (disease* or fever* or infect* or strain$1 or virus* or epidemic* or outbreak* or pandemic*)).tw. (1771) |
| 7 | | (marburg* adj10 (disease* or fever* or infect* or strain$1 or virus* or epidemic* or outbreak* or pandemic*)).tw. (1047) |
| 8 | | Hemorrhagic Fever, Ebola/ (349) |
| 9 | | (h?emorrhagic fever$1 adj10 (virus* or viral*)).tw. (3050) |
| 10 | | Nairo virus/ (655) |
| 11 | | Nairovirus infection/ (4) |
| 12 | | (nairovir* or nairovir* or dugbevir* or dugbevir*).tw. (217) |
| 13 | | Crimean Congo hemorrhagic fever/ (540) |
| 14 | | ((congo* or crimean*) adj10 (disease* or fever* or infect* or strain$1 or virus* or epidemic* or outbreak* or pandemic*)).tw. (3358) |
| 15 | | arenavirus infection/ (113) |
| 16 | | (arenavir* or arena vir*).tw. (1108) |
| 17 | | Lassa fever/ (636) |
| 18 | | Lassa virus/ (696) |
| 19 | | (lassa adj10 (disease* or fever* or infect* or strain$1 or virus* or epidemic* or outbreak* or pandemic*)).tw. (948) |
| 20 | | or/1-19 (10035) |
| 21 | | Shoe/ (8055) |
| 22 | | (shoe or shoes or boot or boots or bootwear* or footwear* or shoewear*).tw. (12750) |
| 23 | | ((foot or feet) adj3 (apparel* or cover* or hygien* or precaution* or protect* or prophyla* or seal* or shield* or wear*)).tw. (922) |
| 24 | | Scalp/ (17759) |
| 25 | | ((head$1 or hair$1 or scalp$1) adj3 (apparel* or cover* or hygien* or precaution* or protect* or prophyla* or seal* or shield* or wear*)).tw. (2830) |
| 26 | | Gas mask/ (1229) |
| 27 | | (respirator or respirators or gasmask*).tw. (6918) |
| 28 | | (respiratory adj3 (device* or mask$1 or facecover* or face cover* or facialcover* or facial cover* or facemask* or facialmask* or facepiece* or face piece* or facialpiece* or facial piece* or faceseal* or face seal* or facialseal* or facial seal* or faceshield* or face shield* or facialshield* or facial shield*)).tw. (879) |
| 29 | | ((N95 or N-95) adj3 (device* or mask$1 or facecover* or face cover* or facialcover* or facial cover* or facemask* or facialmask* or facepiece* or face piece* or facialpiece* or facial piece* or faceseal* or face seal* or facialseal* or facial seal* or faceshield* or face shield* or facialshield* or facial shield*)).tw. (203) |
| 30 | | or/21-29 (44197) |
| 31 | | 20 and 30 (8) |
| 32 | | exp animal experimentation/ or exp models animal/ or exp animal experiment/ or nonhuman/ or exp vertebrate/ (20759208) |
| 33 | | exp humans/ or exp human experimentation/ or exp human experiment/ (15173597) |
| 34 | | 32 not 33 (5586623) |
| 35 | | 31 not 34 (6) |
| 36 | | limit 35 to (meta analysis or "systematic review") (0) |
| 37 | | exp meta-analysis as topic/ (14688) |
| 38 | | (meta-analy* or metanaly* or metaanaly* or met analy* or integrative research or integrative review* or integrative overview* or research integration or research overview* or collaborative review*).tw. (86130) |
| 39 | | (systematic review* or systematic overview* or evidence-based review* or evidence-based overview* or (evidence adj3 (review* or overview*)) or meta-review* or meta-overview* or meta-synthes* or "review of reviews" or technology assessment* or HTA or HTAs).tw. (101742) |
| 40 | | exp Technology assessment, biomedical/ (11390) |
| 41 | | (cochrane or health technology assessment or evidence report).jw. (12627) |
| 42 | | or/37-41 (189873) |
| 43 | | 35 and 42 (0) |
| 44 | | 36 or 43 (0) |
| 45 | | exp practice guideline/ (317013) |
| 46 | | (guidance* or guideline* or standards or recommendation*).ti. (129199) |
| 47 | | (expert consensus or consensus statement* or consensus conference* or practice parameter* or position statement* or policy statement* or CPG or CPGs).tw. (42409) |
| 48 | | or/45-47 (425043) |
| 49 | | 35 and 48 (0) |
| 50 | | randomized controlled trial/ or controlled clinical trial/ (484018) |
| 51 | | exp "clinical trial (topic)"/ (112362) |
| 52 | | (randomi#ed or randomly or RCT$1 or placebo*).tw. (818854) |
| 53 | | ((singl* or doubl* or trebl* or tripl*) adj (mask* or blind* or dumm*)).tw. (169311) |
| 54 | | trial.ti. (169922) |
| 55 | | or/50-54 (1160730) |
| 56 | | 35 and 55 (0) |
| 57 | | controlled clinical trial/ (386510) |
| 58 | | "controlled clinical trial (topic)"/ (3101) |
| 59 | | (control* adj2 trial*).tw. (183833) |
| 60 | | (nonrandom* or non-random* or quasi-random* or quasi-experiment*).tw. (40587) |
| 61 | | (nRCT or nRCTs or non-RCT$1).tw. (393) |
| 62 | | (control* adj3 ("before and after" or "before after")).tw. (3429) |
| 63 | | time series analysis/ (14284) |
| 64 | | time series.tw. (18388) |
| 65 | | pretest posttest control group design/ (205) |
| 66 | | (pre- adj3 post-).tw. (70226) |
| 67 | | (pretest adj3 posttest).tw. (3359) |
| 68 | | controlled study/ (4392797) |
| 69 | | (control* adj2 stud$3).tw. (196131) |
| 70 | | control group/ (76180) |
| 71 | | (control$ adj2 group$1).tw. (442995) |
| 72 | | trial.ti. (169922) |
| 73 | | or/57-72 (4934533) |
| 74 | | 35 and 73 (0) |
| 75 | | cohort analysis/ (174579) |
| 76 | | cohort$1.tw. (411252) |
| 77 | | retrospective study/ (355961) |
| 78 | | longitudinal study/ (69294) |
| 79 | | prospective study/ (259228) |
| 80 | | (longitudinal or prospective or retrospective).tw. (1012969) |
| 81 | | follow up/ (847046) |
| 82 | | ((followup or follow-up) adj (study or studies)).tw. (51677) |
| 83 | | observational study/ (59135) |
| 84 | | (observation$2 adj (study or studies)).tw. (67273) |
| 85 | | population research/ (69751) |
| 86 | | ((population or population-based) adj (study or studies or analys#s)).tw. (14902) |
| 87 | | ((multidimensional or multi-dimensional) adj (study or studies)).tw. (110) |
| 88 | | exp comparative study/ (1026953) |
| 89 | | ((comparative or comparison) adj (study or studies)).tw. (107262) |
| 90 | | exp case control study/ (87332) |
| 91 | | ((case-control* or case-based or case-comparison) adj (study or studies)).tw. (78663) |
| 92 | | or/75-91 (3209316) |
| 93 | | 35 and 92 (0) |
| 94 | | case report/ (1968769) |
| 95 | | case study/ (36402) |
| 96 | | (case adj (report$1 or study or studies or history or histories or series)).tw. (472580) |
| 97 | | cross-sectional study/ (119180) |
| 98 | | (cross-section* adj3 (study or studies or analy* or survey*)).tw. (153493) |
| 99 | | or/94-98 (2332274) |
| 100 | | 35 and 99 (1) |
| 101 | | 44 or 49 or 56 or 74 or 93 or 100 (1) |
| 102 | | (editorial or letter).pt. (1306204) |
| 103 | | 101 not 102 (1) |
|  | | |
| **The Cochrane Database of Systematic Reviews**  **(Limited to the Cochrane Infectious Disease Review Group)** | | |
| **Virus Type:** Filoviruses (Ebola, Marburg)  **Search Date:** July 28, 2014 | | |
| 1 | | [mhFiloviridae] 2 |
| 2 | | [mh "Filoviridae Infections"] 3 |
| 3 | | filovirida*:ti,ab,kw 0 |
| 4 | | Ebolavir* or Marburgvir* or Filovir* or ((Ebola or Marburg or Filo) next vir*):ti,ab,kw 3 |
| 5 | | (BDBV or EBOV or RESTV or SUDV or TAFV):ti,ab,kw 1 |
| 6 | | [mh "Hemorrhagic Fever, Ebola"] 3 |
| 7 | | (ebola* near/10 (disease* or fever* or infect* or strain* or virus* or epidemic* or outbreak* or pandemic*)):ti,ab,kw 4 |
| 8 | | (marburg* near/10 (disease* or fever* or infect* or strain* or virus* or epidemic* or outbreak* or pandemic*)):ti,ab,kw 2 |
| 9 | | {or #1-#8} 6 |
| When restricted to Infectious Disease Group | | |
| 9 | | {or #1-#8} with Infectious Diseases Group in Review Groups 0 |
|  | | |
| **The Cochrane Database of Systematic Reviews**  **(CDSR & Central)** | | |
| **Virus Type:**Filoviruses & Viral Hemorrhagic Fevers  **PPE:** Gloves, Face protection and Gowns  **Search Date:** July 28, 2014 | | |
| 1 | | [mhFiloviridae] 2 |
| 2 | | [mh "Filoviridae Infections"] 3 |
| 3 | | filovirida*:ti,ab,kw 0 |
| 4 | | Ebolavir* or Marburgvir* or Filovir* or ((Ebola or Marburg or Filo) next vir*):ti,ab,kw 3 |
| 5 | | (BDBV or EBOV or RESTV or SUDV or TAFV):ti,ab,kw 1 |
| 6 | | [mh "Hemorrhagic Fever, Ebola"] 3 |
| 7 | | (ebola* near/10 (disease* or fever* or infect* or strain* or virus* or epidemic* or outbreak* or pandemic*)):ti,ab,kw 4 |
| 8 | | [mh "Marburg Virus Disease"] 0 |
| 9 | | (marburg* near/10 (disease* or fever* or infect* or strain* or virus* or epidemic* or outbreak* or pandemic*)):ti,ab,kw 2 |
| 10 | | [mh ^"Hemorrhagic Fevers, Viral"] 1 |
| 11 | | (h*emorrhagic next fever*) near/10 (virus* or viral*):ti,ab,kw 31 |
| 12 | | [mh ^Nairovirus] 0 |
| 13 | | nairovir* or dugbevir* or ((nairo or dugbe) next vir*):ti,ab,kw 0 |
| 14 | | [mh "Hemorrhagic Fever Virus, Crimean-Congo"] 1 |
| 15 | | [mh "Hemorrhagic Fever, Crimean"] 5 |
| 16 | | ((congo* or crimean*) near/10 (disease* or fever* or infect* or strain* or virus* or epidemic* or outbreak* or pandemic*)):ti,ab,kw 28 |
| 17 | | [mh ^"Arenaviridae Infections"] 0 |
| 18 | | arenavir* or (arena next vir*):ti,ab,kw 1 |
| 19 | | [mh "Lassa Fever"] 0 |
| 20 | | [mh "Lassa virus"] 0 |
| 21 | | (lassa near/10 (disease* or fever* or infect* or strain* or virus* or epidemic* or outbreak* or pandemic*)):ti,ab,kw 0 |
| 22 | | [mh Cholera] 195 |
| 23 | | [mh ^"Vibrio cholerae"] 87 |
| 24 | | (cholera* or choleric*):ti,ab,kw 433 |
| 25 | | {or #1-#24} 491 |
| 26 | | [mh ^"Communicable Disease Control"] 110 |
| 27 | | [mh "Infection Control"] 1178 |
| 28 | | [mh "Cross Infection"/pc] 773 |
| 29 | | [mh "Disease Outbreaks"/pc] 143 |
| 30 | | [mh "Disease Transmission, Infectious"/pc] 472 |
| 31 | | [mh "Virus Diseases"/pc] 5758 |
| 32 | | ((diseas* or infect* or virus*) near/5 (prevent* or precaution* or control* or eliminat* or manag* or reduc* or stop*)):ti,ab,kw 45393 |
| 33 | | (epidemic* or outbreak* or pandemic*) near/5 (prevent* or precaution* or control* or eliminat* or manag* or reduc* or stop):ti,ab,kw 340 |
| 34 | | {or #26-#33} 49178 |
| 35 | | #25 and #34 66 |
| 36 | | [mh ^"Protective Devices"] 227 |
| 37 | | [mh "Protective Clothing"] 418 |
| 38 | | ((precaution* or protect* or prophyla*) near/3 (apparel* or attire* or barrier* or cloth* or device* or equipment* or garment* or gear or layer* or material* or workwear)):ti,ab,kw 1256 |
| 39 | | PPE:ti,ab,kw 47 |
| 40 | | [mh "Eye Protective Devices"] 43 |
| 41 | | eyecover* or eyeglasses or eyevisor* or eyewear* or glasses* or goggle* or (eye* near/2 visor*):ti,ab,kw 766 |
| 42 | | [mh ^Masks] 354 |
| 43 | | (mask or masks or facemask* or facialmask* or faceseal* or facialseal* or faceshield* or facialshield*):ti,ab,kw 10609 |
| 44 | | ((eye or eyes or face or faces or facial* or ear or ears or mouth* or body or bodies or skin or hand or hands) near/3 (cover* or hygien* or precaution* or protect* or prophyla* or seal* or shield*)):ti,ab,kw 1321 |
| 45 | | glove or gloves or gloving or ((single or double or triple) next glov*):ti,ab,kw 620 |
| 46 | | [mh ^"Surgical Attire"] 3 |
| 47 | | apron* or gown or gowns or scrubs or (theatre next blues):ti,ab,kw 424 |
| 48 | | ((surgical or surger*) near/3 (apparel* or attire* or cloth* or garment* or workwear)):ti,ab,kw 42 |
| 49 | | [mh "Occupational exposure"/pc] 63 |
| 50 | | (occupation* near/3 (expos* or contaminat* or infect* or transmission* or transmit*) near/5 (prevent* or precaution* or control* or eliminat* or manag* or reduc* or stop*)):ti,ab,kw 169 |
| 51 | | ((personnel* or HCW or HCWs or worker* or (health* near/3 professional*) or clinician* or doctor* or nurse* or physician* or practitioner*) near/3 (expos* or contaminat* or infect* or transmission* or transmit*) near/5 (prevent* or precaution* or control* or eliminat* or manag* or reduc* or stop*)):ti,ab,kw 189 |
| 52 | | (occupational near/3 (hygiene or safety)):ti,ab,kw 113 |
| 53 | | [mh "Universal precautions"] 12 |
| 54 | | ((safety or standard* or universal) near/3 precaution*):ti,ab,kw 55 |
| 55 | | [mh "Equipment Contamination"/pc] 172 |
| 56 | | (contaminat* near/5 (prevent* or precaution* or control* or eliminat* or manag* or reduc* or stop*)):ti,ab,kw 630 |
| 57 | | {or #36-#56} 15194 |
| 58 | | #25 and #57 8 |
| 59 | | #35 or #58 with Infectious Diseases Group in Review Groups 25 |
| CDSR-5; Central-20 | | |
|  | | |
| **Virus Type:**Filoviruses & Viral Hemorrhagic Fevers  **PPE:** Respirators, Foot and Scalp Protection  **Search Date:** August 7, 2014 | | |
| 1 | [mhFiloviridae] 2 | |
| 2 | [mh "Filoviridae Infections"] 3 | |
| 3 | filovirida*:ti,ab,kw 0 | |
| 4 | Ebolavir* or Marburgvir* or Filovir* or ((Ebola or Marburg or Filo) next vir*):ti,ab,kw 3 | |
| 5 | (BDBV or EBOV or RESTV or SUDV or TAFV):ti,ab,kw 1 | |
| 6 | [mh "Hemorrhagic Fever, Ebola"] 3 | |
| 7 | (ebola* near/10 (disease* or fever* or infect* or strain* or virus* or epidemic* or outbreak* or pandemic*)):ti,ab,kw 4 | |
| 8 | [mh "Marburg Virus Disease"] 0 | |
| 9 | (marburg* near/10 (disease* or fever* or infect* or strain* or virus* or epidemic* or outbreak* or pandemic*)):ti,ab,kw 2 | |
| 10 | [mh ^"Hemorrhagic Fevers, Viral"] 1 | |
| 11 | (h*emorrhagic next fever*) near/10 (virus* or viral*):ti,ab,kw 31 | |
| 12 | [mh ^Nairovirus] 0 | |
| 13 | nairovir* or dugbevir* or ((nairo or dugbe) next vir*):ti,ab,kw 0 | |
| 14 | [mh "Hemorrhagic Fever Virus, Crimean-Congo"] 1 | |
| 15 | [mh "Hemorrhagic Fever, Crimean"] 5 | |
| 16 | ((congo* or crimean*) near/10 (disease* or fever* or infect* or strain* or virus* or epidemic* or outbreak* or pandemic*)):ti,ab,kw 28 | |
| 17 | [mh ^"Arenaviridae Infections"] 0 | |
| 18 | arenavir* or (arena next vir*):ti,ab,kw 1 | |
| 19 | [mh "Lassa Fever"] 0 | |
| 20 | [mh "Lassa virus"] 0 | |
| 21 | (lassa near/10 (disease* or fever* or infect* or strain* or virus* or epidemic* or outbreak* or pandemic*)):ti,ab,kw 0 | |
| 22 | {or #1-#21} 58 | |
| 23 | [mh Shoes] 285 | |
| 24 | (shoe or shoes or boot or boots or bootwear* or footwear* or shoewear*):ti,ab,kw 712 | |
| 25 | ((foot or feet) near/3 (apparel* or cover* or hygien* or precaution* or protect* or prophyla* or seal* or shield* or wear*)):ti,ab,kw 41 | |
| 26 | [mh Scalp] 224 | |
| 27 | ((head or heads or hair or hairs or scalp*) near/3 (apparel* or cover* or hygien* or precaution* or protect* or prophyla* or seal* or shield* or wear*)):ti,ab,kw 269 | |
| 28 | [mh "Respiratory Protective Devices"] 44 | |
| 29 | (respirator or respirators or gasmask*):ti,ab,kw 174 | |
| 30 | (respiratory near/3 (device* or mask or masks or facecover* or (face next cover*) or facialcover* or (facial next cover*) or facemask* or facialmask* or facepiece* or (face next piece*) or facialpiece* or (facial next piece*) or faceseal* or (face next seal*) or facialseal* or (facial next seal*) or faceshield* or (face next shield*) or facialshield* or (facial next shield*))):ti,ab,kw 137 | |
| 31 | (N95 or "N-95") near/3 (device* or mask or masks or facecover* or (face next cover*) or facialcover* or (facial next cover*) or facemask* or facialmask* or facepiece* or (face next piece*) or facialpiece* or (facial next piece*) or faceseal* or (face next seal*) or facialseal* or (facial next seal*) or faceshield* or (face next shield*) or facialshield* or (facial next shield*)):ti,ab,kw 10 | |
| 32 | {or #23-#31} 1504 | |
| 33 | #22 and #32 0 | |
|  | | |
| **African Index Medicus** | | |
| **Search Date:**July 29, 2014 | | |
| Filoviridae OR filovirus OR filoviral OR Ebola OR Ebolavirus OR Marburg or "Hemorragic fever" or "Haemorragic fever"or "Hemorragic fevers" or "Haemorragic fevers"  BDBV OR EBOV OR RESTV OR SUDV OR TAFV  Nairovirus OR dugbevirus OR arenavirus  Nairo AND virus  dugbe AND virus  arenavirus  cholera or choleric (8) | | |
|  | | |
| **Grey Literature Search** | | |
| **Search Date:**August 20 & 22, 2014 | | |
| **ProQuest Dissertations & Theses Full Text ( 20 August 2014)**  53 results  all(Filoviridae OR Ebola OR Ebolavir* OR Marburgvir* OR Filovir* OR "Marburg Virus " OR "Hemorrhagic Fever*" OR "hemorrhagic fever*") AND ((“Protective Devices” OR “Protective Clothing” OR PPE OR (eyecover* OR eyeglasses OR eyevisor* OR eyewear* OR glasses* OR goggles OR goggle OR ((eyes OR eye) and visor*)) OR (masks OR mask OR facemask* OR facialmask* OR faceseal* OR facialseal* OR faceshield* OR facialshield*) OR gloves OR glove OR gloving* OR apron* OR gown OR gowns OR scrubs OR “theatre blues” OR ((surgical OR surger*) AND (apparel* OR attire* OR cloth* OR garment* OR workwear*))) AND (Filoviridae OR Ebola OR Ebolavir* OR Marburgvir* OR Filovir* OR "Marburg Virus " OR "Hemorrhagic Fever*" OR "hemorrhagic fever*"))  **Google search : 22 August 2014**  **Using Google search interface, the following sites were searched using**  Ebola and « personal protection » and site :cdc.gov  Ebola and « personal protection » and site :europa.eu  Ebola and « personal protection » and site :se  Ebola and « personal protection » and site :gov.au  Ebola and « personal protection » and site :gov.uk  Ebola and « personal protection » and site :gc.ca  Ebola and « personal protection » and site :books.google.com  Ebola and « personal protection » and site :msf.org  Ebola and « personal protection » and site :opengrey.eu  Ebola and « personal protection » and site :gao.gov  Ebola and « personal protection » and site :no  Ebola and « personal protection » and site :gov.sa  Results   \| Ebola and « personal protection » and site :cdc.gov filetype:pdf \| 162 \| \| --- \| --- \| \| Ebola and « personal protection » and site:europa.eu filetype:pdf \| 204 \| \| Ebola and « personal protection » and site:se filetype:pdf \| 74 \| \| Ebola and « personal protection » and site:gov.au filetype:pdf \| 115 \| \| Ebola and « personal protection » and site:gov.uk filetype:pdf \| 114 \| \| Ebola and « personal protection » and site:gc.ca filetype:pdf \| 65 \| \| Ebola and « personal protection » and site:books.google.com filetype:pdf \| 4 \| \| Ebola and « personal protection » and site:msf.org filetype:pdf \| 31 \| \| Ebola and « personal protection » and site:opengrey.eu filetype:pdf \| 0 \| \| Ebola and « personal protection » and site:gao.gov filetype:pdf \| 23 \| \| Ebola and « personal protection » and site:no filetype:pdf \| 32 \| \| Ebola and « personal protection » and site:gov.sa filetype:pdf \| 3 \| | | |
