# Supplementary material for: Effectiveness of Personal Protective Equipment for Healthcare Workers Caring for Patients with Filovirus Disease: A Rapid Review
Source: PLoS One. 2015 Oct 9;10(10):e0140290. doi: 10.1371/journal.pone.0140290 (PMC4599797; doi:10.1371/journal.pone.0140290)
Supplement: S4 Appendix — (DOCX) [file pone.0140290.s004.docx]

S4 Appendix. List of studies excluded (and reasons) during full text screening.

| **Bibliography** |
| --- |
| **Full-text not available / Potentially relevant studies** |
| Ajazaj-Berisha, L., Ahmeti, S., Dreshaj, Sh, Namani, S., Qehaja-Buqaj, E., Vishaj, A., and Halili, B.. Nosocomial infection of Crimean-Congo hemorrhagic fever in Kosovo. European Journal of Internal Medicine 2013; 24: e207. |
| Caylan, R., Yapar, D., Keske, S., Hasanoglu, I., and Tasyaran, M. A.. Nosocomial transmission of Crimean-Congo haemorrhagic fever. Clinical Microbiology and Infection 2010; 16: S700. |
| Centers for Disease Control (CDC). Viral hemorrhagic fever: initial management of suspected and confirmed cases. MMWR - Morbidity & Mortality Weekly Report 1983; 32 Suppl : 27S-38S. |
| Chowa, GCE. What you need to know about cholera: reminder of the dangers of cholera disease. Moyo XXIII 2014; 15-18. |
| Elshner, M.. BSL3 and BSL4 Agents: Epidemiology, Microbiology and Practical Guidelines. Wiley-Blackwell 2012. |
| Ettenger, V.. Ebola and Marburg: History of natural epidemics and assessment of potential BW threat. Anne Arbor: George Mason University 2006; 1-222. |
| Grant, AM.. Countermeasures against viral hemorrhagic fevers. Anne Arbor: The University of Texas Medical Branch Graduate School of Biomedical Sciences 2012; 1-73. |
| Lapios, JC.. Les fievres hemorragiques virales: mise a jour des connaissances sur les filoviridae 2002. |
| Malawi Ministry of Health. Community Health Sciences Unit. Epidemiologic exercise: ebola virus haemorrhagic fever 2014; 1-38. |
| Niklasson, B.. Ebola virus: causing a rare but deadly disease in Africa: haemorrhagic fever viruses. Africa Review 1996; 21-22. |
| Snowden, A.. Marburg disease: the 20th Century. Zimbabwe Rhodesia Nurse 1979; 59-81. |
| Vorou, R.. Control of vector-borne diseases in Europe: Mini-review article. Review of Clinical Pharmacology and Pharmacokinetics, International Edition 2010; 24: 239-246. |
| **Study was not in English or French** |
| Barreto, A, Tallarico, M, and Lopez, MA. C. Revista m 6 1995; 2-5. |
| Domingo-Carrasco, C. and Gascon-Bustrenga, J.. Dengue and other hemorrhagic viral fevers. Enfermedades Infecciosas y Microbiologia Clinica 2005; 23: 615-626. |
| Ferrari, C. K. and Torres, E. A.. Viral contamination of food products: a poorly understood public health problem. Pan American Journal of Public Health 1998; 3: 359-366. |
| Fleischer, K., Kohler, B., Kirchner, A., and Schmid, J.. Lassa fever. Medizinische Klinik 2000; 95: 340-345. |
| Furnsinn, G. and Harbich, H.. Filoviral infections in humans - Epidemiology, therapy and prevention. Wiener Klinische Wochenschrift 2000; 112: 30-35. |
| Harboe, Z. B., Qureshi, K. M., Skinhoj, P., and Heegaard, E. D.. Marburg haemorrhagic fever in Angola, 2005. Ugeskrift for Laeger 2005; 167: 4087-4090. |
| Hofler, W.. Recommendations for traveling. ZFA. Zeitschrift fur Allgemeinmedizin 1983; 59: 1407-1410. |
| Hofler, W.. Recommendations for traveling. ZFA - Zeitschrift fur Allgemeinmedizin 1983; 59: 1407-1410. |
| Hwang, H. S.. The strategic plan for preparedness and response to bioterrorism in Korea. Journal of Preventive Medicine & Public Health / Yebang Uihakhoe Chi 2008; 41: 209-213. |
| Hwang, H. S.. The strategic plan for preparedness and response to bioterrorism in Korea. Journal of Preventive Medicine & Public Health / Yebang Uihakhoe Chi 2008; 41: 209-213. |
| Jezek, Z.. Ebola fever: An emerging disease. Epidemiologie, Mikrobiologie, Imunologie 2001; 50: 54-66. |
| Jezek, Z. and Vacek, V.. Filoviruses - Growing threat. Klinicka Mikrobiologie a Infekcni Lekarstvi 2001; 7: 30-38. |
| Kusnick, C.. Permanent Vaccination Commission: New vaccination recommendations from the Permanent Vaccination Commission. Deutsche Apotheker Zeitung 2010; 150: 35-36. |
| Lentze, M.. Vaccination recommendations of the standing vaccination commission (STIKO) at the Robert Koch Institut. Monatsschrift fur Kinderheilkunde 2001; 149: 332-343. |
| Lutkes, P., Witzke, O., Philipp, Th, Schmitt, H. J., and Heemann, U.. Immunization and medical recommendations for patients travelling after transplantation. Deutsche Medizinische Wochenschrift 2000; 125: 1011-1016. |
| Raoofi, R., Pourahamad, M., Nazer, M. R., Pournia, Y., and Chinikar, S.. Case series of Crimean-Congo disease: An outbreak in south of Fars, Iran. Journal of Babol University of Medical Sciences 2012; 14: 96-100. |
| Ritter, M.. ... Another new wonder drug!.Chirurgische Praxis 2001; 58: 769-770. |
| Sallent, L. V. and Cuevas, O. M.. Advice to travelers: Prevention of non-infectious diseases. FMC Formacion Medica Continuada en Atencion Primaria 2008; 15: 1-39. |
| Schienger, R.. Healthy through the holidays - Current travel recommendations. Deutsche Apotheker Zeitung 2009; 149: 56-63. |
| Schwarz, T. F.. Travelling during pregnancy. Vaccination, yes or no?.Munchener Medizinische Wochenschrift 1999; 141: 12-14. |
| Stock, I.. Infectious diseases. Part 1: Tuberculosis, diphteria, anthrax and cholera. PZ Prisma 2003; 10: 229-243. |
| Takeda, Y. and Nomura, T.. [Future direction of medical care system for patients with infectious disease and the new infectious diseases control law in Japan--centering around a category 1 hospital]. [Japanese]. Kansenshogaku Zasshi - Journal of the Japanese Association for Infectious Diseases 2000; 74: 687-693. |
| Takeda, Y. and Nomura, T.. Future direction of medical care system for patients with infectious disease and the new infectious diseases control law in Japan--centering around a category 1 hospital. Kansenshogaku zasshi.The Journal of the Japanese Association for Infectious Diseases 2000; 74: 687-693. |
| Tanaka, T., Takahashi, H., Ohyama, T., Okabe, N., and Uchida, Y.. [Management of patients with viral hemorrhagic fever in Germany a comparison with the current Japanese system based on the who guidelines on emerging viral diseases]. [Japanese]. Nippon Koshu Eisei Zasshi - Japanese Journal of Public Health 2002; 49: 564-573. |
| Tanaka, T., Takahashi, H., Ohyama, T., Okabe, N., and Uchida, Y.. Management of patients with viral hemorrhagic fever in Germany a comparison with the current Japanese system based on the who guidelines on emerging viral diseases. [Nippon koshu eisei zasshi] Japanese journal of public health 2002; 49: 564-573. |
| Tent, M.. Made-to-measure advice: Vaccinations for travelers. Pharmaceutisch Weekblad 2008; 143: 20-23. |
| Vaccination recommendations of the permanent vaccination commission (STIKO) - State: October 1994. Zeitschrift fur Arztliche Fortbildung 1995; 89: 253-264. |
| Yamaguchi, T., Tsuji, M., and Imagawa, Y.. A first Lassa fever from Sierra Leone treated in high security ward. Nippon Rinsho - Japanese Journal of Clinical Medicine 1989; 47: 76-81. |
| **No PPE use among HCWs in Ebola treatment centres/general wards** |
| A case of cholera. Communicable diseases intelligence 1998; 22: 154. |
| Abrams, A. and Kredo, T.. Vaccine research, adolescents and Africa. Human Vaccines and Immunotherapeutics 2013; 9: 108-111. |
| Abuova, G., Pshenichnaya, N., Irsimbetova, N., and Apsatarov, Z.. Clinical and epidemiological aspects of Crimean-Congo hemorrhagic fever in pregnant women in South Kazakhstan. International Journal of Infectious Diseases 2012; 16: e66. |
| Adjemian, J., Farnon, E. C., Tschioko, F., Wamala, J. F., Byaruhanga, E., Bwire, G. S., Kansiime, E., Kagirita, A., Ahimbisibwe, S., Katunguka, F., Jeffs, B., Lutwama, J. J., Downing, R., Tappero, J. W., Formenty, P., Amman, B., Manning, C., Towner, J., Nichol, S. T., and Rollin, P. E.. Outbreak of Marburg hemorrhagic fever among miners in kamwenge and ibanda Districts, Uganda, 2007. Journal of Infectious Diseases 2011; 204: S796-S799. |
| Ajelli, M. and Merler, S.. Transmission Potential and Design of Adequate Control Measures for Marburg Hemorrhagic Fever. PLoS ONE [Electronic Resource] 7 (2012): e50948-1-8. |
| Al, Nsour M., Kaiser, R., Elkreem, E. A., Walke, H., Kandeel, A., and Bloland, P.. Highlights and conclusions from the eastern mediterranean public health network (EMPHNET) conference 2011. Eastern Mediterranean Health Journal 2012; 18: 189-191. |
| Al-Tawfiq, J. A. and Memish, Z. A.. The Hajj: Updated health hazards and current recommendations for 2012. Eurosurveillance 2012; 17: 1-5. |
| Al-Tikriti, S. K., Al-Ani, F., Jurji, F. J., Tantawi, H., Al-Moslih, M., Al-Janabi, N., Mahmud, M. I., Al-Bana, A., Habib, H., Al-Munthri, H., Al-Janabi, S., AL-Jawahry, K., Yonan, M., Hassan, F., and Simpson, D. I.. Congo/Crimean haemorrhagic fever in Iraq. Bull World Health Organ 1981; 59: 85-90. |
| An isolated case of Marburg. Nursing Times 1977; 73: 262-263. |
| Antosia, R., Cahill, J.D.. Handbook of bioterrorism and disaster medicine. Springer Sciences & Business Media. 2006: 1-510. |
| Aradaib, I.E., Erickson, B.R., Mustafa, M.E., Khristova, M.L., Saeed, N.S., Elageb, R.M., Nichol, S.T.. Nosocomial outbreak of Crimean-Congo Hemorrhagic Fever, Sudan. 2010: 837-839. |
| Atkinson, B., Chamberlain, J., Jameson, L. J., Logue, C. H., Lewis, J., Belobrova, E. A., Valikhodzhaeva, M., Mullojonova, M., Tishkova, F. H., and Hewson, R.. Identification and analysis of Crimean-Congo hemorrhagic fever virus from human sera in Tajikistan. International Journal of Infectious Diseases 2013; 17: 1031-1037. |
| Baird, R. and Cohen, J.. Travellers' diarrhoea in general practice. Medicine Today 2002; 3: 69-72. |
| Baltazar, J., Briscoe, J., Mesola, V., Moe, C., Solon, F., Vanderslice, J., and Young, B.. Can the case-control method be used to assess the impact of water supply and sanitation on diarrhoea? A study in the Philippines. Bulletin of the World Health Organization 1988; 66: 627-635. |
| Bangash, S. A. and Khan, E. A.. Treatment and prophylaxis with ribavirin for Crimean-Congo Hemorrhagic Fever--is it effective?.JPMA - Journal of the Pakistan Medical Association 2003; 53: 39-41. |
| Baron, R. C., McCormick, J. B., and Zubeir, O. A.. Ebola virus disease in southern Sudan: hospital dissemination and intrafamilial spread. Bull World Health Organ 1983; 61: 997-1003. |
| Bateman, C.. Doctors overwhelmed at the AIDS coalface. South African Medical Journal 2002; 92: 402-403. |
| Bateman, C.. Sharp eyes on cholera flashpoints. South African Medical Journal 91 (2001): 278-279. |
| Bausch, D. G., Towner, J. S., Dowell, S. F., Kaducu, F., Lukwiya, M., Sanchez, A., Nichol, S. T., Ksiazek, T. G., and Rollin, P. E.. Assessment of the risk of Ebola virus transmission from bodily fluids and fomites. Journal of Infectious Diseases 2007; 196: S142-S147. |
| Bignardi, G. E.. The new viral haemorrhagic fever infection control guidelines. Journal of Hospital Infection 1998; 39: 169-172. |
| Bowell, E.. Journal of Infection Control Nursing. Nursing the isolated patient: Lassa fever. Nursing Times 1986; 82: 72-81. |
| Bowen, E. T., Lloyd, G., Harris, W. J., Platt, G. S., Baskerville, A., and Vella, E. E.. Viral haemorrhagic fever in southern Sudan and northern Zaire. Preliminary studies on the aetiological agent. Lancet 1977; 1: 571-573. |
| Calain, P., Fiore, N., Poncin, M., and Hurst, S. A.. Research ethics and international epidemic response: The case of ebola and marburg hemorrhagic fevers. Public Health Ethics 2009; 2: 7-29. |
| Catheters and epidemics. South African Medical Journal 2002; 92: 559. |
| Centre for Disease Control and Prevention. Agent summary statements: arboviruses and related zoonotic viruses: 233-267. |
| Centre for Disease Control and Prevention. Crafting ebola prevention messages in Uganda. 2009. |
| Centre for Disease Control and Prevention. NIOSH interim guidance on the use of chemical, biological, radiological and nuclear (CBRN) full facepiece, air-purifying respirators/gas masks certified under 42 CFR part 84. 2005. |
| Centre for Disease Control and Prevention. Infectious Diseases- Snapshot 2008. 2008:1-72. |
| Centre for Disease Control and Prevention. Promoting health and quality of life by preventing and controlling vector-borne diseases. 2011: 1-2. |
| Cholera. CDSC Surveillance and Information Division. CDR (London, England : Review) 1991; 1: R48-R50. |
| Cholera in Europe. Releve epidemiologique hebdomadaire / Section d'hygiene du Secretariat de la Societe des Nations = Weekly epidemiological record / Health Section of the Secretariat of the League of Nations 1994; 69: 322-323. |
| Clark, D. V., Jahrling, P. B., and Lawler, J. V.. Clinical management of filovirus-infected patients. Viruses 2012; 4: 1668-1686. |
| Clemens, J. and Bloom, B.. The work of the International Vaccine Institute. Vaccines: Children and Practice 2003; 6: 43-45. |
| Cohen, J.. Australian immunisation guidelines for international travellers. Medicine Today 2014; 15: 57-61. |
| Communicable disease report United Kingdom--January to March 1982. Community Medicine 1982; 4: 238-241. |
| Conrad, J. L., Isaacson, M., Smith, E. B., Wulff, H., Crees, M., Geldenhuys, P., and Johnston, J.. Epidemiologic investigation of Marburg virus disease, Southern Africa, 1975. Am J Trop Med Hyg. 1978; 27: 1210-1215. |
| Control of diarrhoeal diseases: WHO's programme takes shape. WHO Chronicle 32 (1978): 369-372. |
| Cooper, C. B., Gransden, W. R., Webster, M., King, M., O'Mahony, M., Young, S., and Banatvala, J. E.. A case of Lassa fever: experience at St Thomas's Hospital. British Medical Journal Clinical Research Ed . 1982; 285: 1003-1005. |
| Corrections to 5 year efficacy of a bivalent killed whole-cell oral cholera vaccine in Kolkata, India: A cluster-randomised, double-blind, placebo-controlled trial [Lancet Infect Dis (2013)]. The Lancet Infectious Diseases 2013; 13: 1050-1056. |
| Crowcroft, N. S., Meltzer, M., Evans, M., Shetty, N., Maguire, H., Bahl, M., Gair, R., Brink, N., Lockwood, D., Gregor, S., Jones, J., Nicoll, A., Gopal, R., Brown, D., and Bannister, B.. The public health response to a case of Lassa fever in London in 2000. Journal of Infection 2004; 48: 221-228. |
| Danzinger-Isakov, L. and Kumar, D.. Guidelines for vaccination of solid organ transplant candidates and recipients. American Journal of Transplantation 2009; 9: S258-S262. |
| de, Wit E., Feldmann, H., and Munster, V. J.. Tackling Ebola: New insights into prophylactic and therapeutic intervention strategies. Genome Medicine 2011; 3: 1-10. |
| DEFRA Advisory Committee on Dangerous Pathogens. Biological agents- the principles, design and operation of Containment Level 4 facilities. 2006: 1-83. |
| Desai, S. N., Sahastrabuddhe, S., Leon, Ochiai R., and Wierzba, T. F.. Enteric vaccines for resource-limited countries:Current status and future prospects. Pediatric Annals 2011; 40: 351-357. |
| E-alert 24 July: Case of Lassa fever imported into Germany from Sierra Leone. Euro Surveillance: Bulletin Europeen sur les Maladies Transmissibles = European Communicable Disease Bulletin 2006; 11: E060727. |
| Ebola haemorrhagic fever. Wkly Epidemiol Rec. 1996; 71: 359. |
| Eeckels, R. Choléra. 1992: 1075-1083. |
| Emerging infectious diseases: memorandum from a WHO meeting. Bull World Health Organ 1994; 72: 845-850. |
| Enria, D. A. and Barrera Oro, J. G.. Junin virus vaccines. Current Topics in Microbiology & Immunology 2002; 263: 239-261. |
| Enserink, M.. A Global Fire Brigade Responds to Disease Outbreaks. Science 2004; 303: 1605-1606. |
| Ergonul, O., Zeller, H., Kilic, S., Kutlu, S., Kutlu, M., Cavusoglu, S., Esen, B., and Dokuzoguz, B.. Zoonotic infections among veterinarians in Turkey: Crimean-Congo hemorrhagic fever and beyond. International Journal of Infectious Diseases 2006; 10: 465-469. |
| European Centre for Disease Prevention and Control. Rapid Risk Assessment: Outbreak of Haemorrhagic fever in Guinea. 2014: 1-7. |
| European Commission. Annex 3- Action fiche for establishment of mobile laboratories for pathogens up to risk group 4 in combination with CBRN capacity building in Sub-Saharan Africa.: 1-12. |
| Evans, N.. Travel with care. Occupational Health 2005; 57: 22-25. |
| Feldmann, H., Jones, S., Klenk, H. D., and Schnittler, H. J.. Ebola virus: from discovery to vaccine. Nature Reviews Immunology. 2003; 3: 677-685. |
| Fernandes, V., Sim, F. M., Kearns, W. E., and Lau, Y. K.. Lassa fever in London: environmental health aspects. Community Medicine 1984; 6: 140-144. |
| Ferreira, R. B. R., Antunes, L. C. M., and Brett, Finlay B.. Should the human microbiome be considered when developing vaccines?.PLoS Pathogens 2010; 6: e1001190-1-2. |
| Frame, J. D., Baldwin, J. M., Jr., Gocke, D. J., and Troup, J. M.. Lassa fever, a new virus disease of man from West Africa. I. Clinical description and pathological findings. Am J Trop Med Hyg. 1970; 19: 670-676. |
| Frame, J. D., Lange, W. R., and Frankenfield, D. L.. Mortality trends of American missionaries in Africa, 1945-1985. American journal of tropical medicine and hygiene 1992; 46: 686-690. |
| Fraser, D. W., Campbell, C. C., Monath, T. P., Goff, P. A., and Gregg, M. B.. Lassa fever in the Eastern Province of Sierra Leone, 1970-1972. I. Epidemiologic studies. Am J Trop Med Hyg. 1974; 23: 1131-1139. |
| Friedrich, B. M., Trefry, J. C., Biggins, J. E., Hensley, L. E., Honko, A. N., Smith, D. R., and Olinger, G. G.. Potential vaccines and post-exposure treatments for filovirus infections. Viruses 2012; 4: 1619-1650. |
| From the Centers for Disease Control. Cholera--international travel, 1992. JAMA 1992; 268: 1648-1649. |
| From the Centers for Disease Control. Cholera associated with imported coconut milk. JAMA 1993; 267: 1320. |
| From the Centers for Disease Control. Update: cholera--Western Hemisphere, and recommendations for treatment of cholera. JAMA 1992; 266: 1186 & 1189. |
| From the Centers for Disease Control and Prevention. Arenavirus infection--Connecticut, 1994. JAMA 1994; 272: 998-999. |
| From the Centers for Disease Control and Prevention. Cholera associated with food transported from El Salvador--Indiana, 1994. JAMA 1995; 273: 1823. |
| From the Centers for Disease Control and Prevention. Fatal illnesses associated with a New World arenavirus--California, 1999-2000. JAMA 2000; 284: 1237-1238 |
| From the Centers for Disease Control and Prevention. Imported cholera associated with a newly described toxigenic Vibrio cholerae O139 strain--California, 1993. JAMA 1993; 270: 428-429. |
| Gandsman, E. J., Aaslestad, H. G., Ouimet, T. C., and Rupp, W. D.. Sabia virus incident at Yale University. American Industrial Hygiene Association Journal 1997; 58: 51-53. |
| Gaya, H. and Barrett, S. P.. Viral haemorrhagic fever guidelines. The Journal of hospital infection 1998; 40: 325-328. |
| Geretti, A. M.. British HIV Association guidelines for immunization of HIV-infected adults 2008. HIV Medicine 2008; 9: 795-848. |
| Greene, M. H.. Impact of the Sahelian drought in Mauritania, West Africa. Lancet 1974; 1: 1093-1097. |
| Gurbuz, Y., Sencan, I., Ozturk, B., and Tutuncu, E.. A case of nosocomial transmission of Crimean-Congo hemorrhagic fever from patient to patient. Int J Infect Dis 2009; 13: e105-e107. |
| Haemorrhagic fever vaccine. Drugs in R & D 1999; 2: 197-198. |
| Hamer, D. H.. Prevention of travel-related illness. Infectious Diseases in Clinical Practice 1997; 6: 221-231. |
| Hammarstrom, L. and Weiner, C. K.. Targeted antibodies in dairy-based products. Advances in Experimental Medicine and Biology 2008; 606: 321-343. |
| Harris, J. B., Ivers, L. C., and Ferraro, M. J.. Case records of the Massachusetts General Hospital. Case 19-2011. A 4-year-old Haitian boy with vomiting and diarrhea. New England Journal of Medicine 2011; 364: 2452-2461. |
| Harxhi, A., Pilaca, A., Delia, Z., Pano, K., and Rezza, G.. Crimean-Congo hemorrhagic fever: a case of nosocomial transmission. Infection 2005; 33: 295-296. |
| Hasan, Z., Mahmood, F., Jamil, B., Atkinson, B., Mohammed, M., Samreen, A., Altaf, L., Moatter, T., and Hewson, R.. Crimean-Congo hemorrhagic fever nosocomial infection in a immunosuppressed patient, Pakistan: case report and virological investigation. Journal of Medical Virology 2013; 85: 501-504. |
| Hewlett, B. S. and Amolat, R. P.. Cultural contexts of Ebola in Northern Uganda. Emerging Infectious Diseases 2003; 9: 1242-1248. |
| Heymann, D. L., Weisfeld, J. S., Webb, P. A., Johnson, K. M., Cairns, T., and Berquist, H.. Ebola hemorrhagic fever: Tandala, Zaire, 1977-1978. J Infect Dis 1980; 142: 372-376. |
| Holmes, G. P., McCormick, J. B., Trock, S. C., Chase, R. A., Lewis, S. M., Mason, C. A., Hall, P. A., Brammer, L. S., Perez-Oronoz, G. I., and McDonnell, M. K.. Lassa fever in the United States. Investigation of a case and new guidelines for management. New England Journal of Medicine 1990; 323: 1120-1123. |
| Holmgren, J., Clemens, J., Sack, D. A., Sanchez, J., and Svennerholm, A. M.. Oral immunization against cholera. Current topics in microbiology and immunology 1989; 146: 197-204. |
| Hotez, P. J.. The four horsemen of the apocalypse: Tropical medicine in the fight against plague, death, famine, and war. American journal of tropical medicine and hygiene 2012; 87: 3-10. |
| Inegbenebor, U., Okosun, J., and Inegbenebor, J.. Prevention of lassa Fever in Nigeria. Transactions of the Royal Society of Tropical Medicine & Hygiene 2010; 104: 51-54. |
| Islam, M. S., Bhuiya, A., and Yunus, M.. Socioeconomic differentials of diarrhoea morbidity and mortality in selected villages of Bangladesh. Journal of Diarrhoeal Diseases Research 1984; 2: 232-237. |
| Johnson, K. M.. Gleanings from the harvest: suggestions for priority actions against Ebola virus epidemics. Journal of Infectious Diseases 1999; 179 Suppl 1: S287-S288. |
| Kadish, C. B. and Steele, R. W.. Preventative health advice for families moving to developing countries. Clinical Pediatrics 2014; 53: 515-523. |
| Karti, S. S., Odabasi, Z., Korten, V., Yilmaz, M., Sonmez, M., Caylan, R., Akdogan, E., Eren, N., Koksal, I., Ovali, E., Erickson, B. R., Vincent, M. J., Nichol, S. T., Comer, J. A., Rollin, P. E., and Ksiazek, T. G.. Crimean-Congo hemorrhagic fever in Turkey. Emerg.Infect Dis 2004; 10: 1379-1384. |
| Khan, A. M., Rahman, A. K. S. M., Hossain, M. S., Faruque, A. S. G., Huq, S., Chisti, M. J., and Salam, M. A.. Nosocomial infections among patients admitted to an urban diarrhoeal-diseases treatment facility in Bangladesh: A preliminary survey. Annals of Tropical Medicine and Parasitology 2008; 102: 89-92. |
| Klenk, H.-D.. Will we have and why do we need an Ebola vaccine?.Nature Medicine 2000; 6: 1322-1323. |
| Kramer, A., Schwebke, I., and Kampf, G.. How long do nosocomial pathogens persist on inanimate surfaces? A systematic review. BMC Infectious Diseases 2006; 6: 1-8. |
| Kuns, M. L.. Epidemiology of Machupo virus infection. II. Ecological and control studies of hemorrhagic fever. American Journal of Tropical Medicine & Hygiene 1965; 14: 813-816. |
| Lassa fever 1982. British Medical Journal Clinical Research Ed . 1983; 287: 48. |
| Laurent, C. and Sorge, F.. Consultation with a child before departure for a developing country. Medecine Therapeutique Pediatrie 2003; 6: 240-251. |
| Lazcano-Ponce, E., Allen, B., and Gonzalez, C. C.. The contribution of international agencies to the control of communicable diseases. Archives of Medical Research 2005; 36: 731-738. |
| Le, Guenno B., Formenty, P., Wyers, M., Gounon, P., Walker, F., and Boesch, C.. Isolation and partial characterisation of a new strain of Ebola virus. Lancet 1995; 345: 1271-1274. |
| Leblebicioglu, H., Bodur, H., Dokuzoguz, B., Elaldi, N., Guner, R., Koksal, I., Kurt, H., and Senturk, G. C.. Case management and supportive treatment for patients with Crimean-Congo hemorrhagic fever. Vector-Borne and Zoonotic Diseases 2012; 12: 805-811. |
| Legrand, J., Grais, R. F., Boelle, P. Y., Valleron, A. J., and Flahault, A.. Understanding the dynamics of Ebola epidemics. Epidemiology & Infection 2007; 135: 610-621. |
| Levine, M. M., Losonsky, G., Herrington, D., Kaper, J. B., Tacket, C., Rennels, M. B., and Morris, J. G.. Pediatric diarrhea: the challenge of prevention. Pediatric Infectious Disease 1986; 5: S29-S43. |
| Lilienfeld, D. E. and Lilienfeld, A. M.. Epidemiology: a retrospective study. American Journal of Epidemiology 1977; 106: 445-459. |
| Lotz, E. and Raffin, H.. Aeromedical evacuation using an aircraft transit isolator of a patient with Lassa fever. Aviation Space & Environmental Medicine 2012; 83: 527-530. |
| Mardani, M., Keshtkar-Jahromi, M., Ataie, B., and Adibi, P.. Crimean-Congo hemorrhagic fever virus as a nosocomial pathogen in Iran. American Journal of Tropical Medicine & Hygiene 2009; 81: 675-678. |
| Mardani, M., Rahnavardi, M., Rajaeinejad, M., Holakoui, Naini K., Chinikar, S., Pourmalek, F., Rostami, M., and Hashemi, Shahri M.. Crimean-Congo hemorrhagic fever among health care workers in Iran: A seroprevalence study in two endemic regions. American journal of tropical medicine and hygiene 2007; 76: 443-445. |
| McLellan, S. L. F.. Vaccines for travelers. Infections in Medicine 2000; 17: 168-171. |
| Medecins Sans Frontieres. Public Health Engineering. In Precarious Situations. 2010:1-414. |
| Meeting of the Strategic Advisory Group of Experts on immunization, April 2014 -- conclusions and recommendations. Weekly Epidemiological Record 2014; 89: 221-236. |
| Meeting of the Strategic Advisory Group of Experts on immunization, October 2009 - conclusions and recommendations. Releve epidemiologique hebdomadaire / Section d'hygiene du Secretariat de la Societe des Nations = Weekly epidemiological record / Health Section of the Secretariat of the League of Nations 2009; 84: 517-532. |
| Memish, Z. A., Fagbo, S. F., Assiri, A. M., Rollin, P., Zaki, A. M., Charrel, R., Mores, C., and MacNeil, A.. Alkhurma viral hemorrhagic fever virus: proposed guidelines for detection, prevention, and control in Saudi Arabia. PLoS Neglected Tropical Diseases [electronic resource] 2012; 6: e1604-1-6. |
| Menon, M. P., Haddock, R. L., Ruben, K., Cooper, K., Greene, K., and Mintz, E. D.. Investigation of an outbreak of cholera among Chuukese residents of Guam, 2005. Pacific Health Dialog 2011; 17: 139-147. |
| Montaner, J. S., Hogg, R., Wood, E., Kerr, T., Tyndall, M., Levy, A. R., and Harrigan, P. R.. The case for expanding access to highly active antiretroviral therapy to curb the growth of the HIV epidemic. Lancet 2006; 368: 531-536. |
| Mupere, E., Kaducu, O. F., and Yoti, Z.. Ebola haemorrhagic fever among hospitalised children and adolescents in northern Uganda: epidemiologic and clinical observations. African Health Sciences 2001; 1: 60-65. |
| Muyembe, T. and Kipasa, M.. Ebola haemorrhagic fever in Kikwit, Zaire. International Scientific and Technical Committee and WHO Collaborating Centre for Haemorrhagic Fevers. Lancet 1995; 345: 1448. |
| Nabeth, P., Thior, M., Faye, O., and Simon, F.. Human Crimean-Congo hemorrhagic fever, Senegal. Emerg.Infect Dis 2004; 10: 1881-1882. |
| National Institute of Justice. Guide for the selection of personal protective equipment for emergency first responders. 2002: 1-118. |
| O'Brien, J.. Vaccine research - 11th annual conference: Cutaneous formulations, universal vaccinations and recently licensed vaccines. IDrugs 2008; 11: 471-474. |
| Okabe, N.. 5. Infectious disease surveillance designated by the Infectious Disease Control Law, and the situation of emerging/re-emerging infectious diseases in Japan. Internal Medicine 2002; 41: 61-62. |
| Orr, K. E. and Wallis, J.. Viral haemorrhagic fever guidelines. The Journal of hospital infection 1998; 40: 325-326. |
| Osborne, N. G.. Infection risks of travelers: Malaria. Journal of Gynecologic Surgery 2003; 19: 103-104. |
| Osterholm, M. T.. Centers for Disease Control says arenavirus spread unlikely. Clinical infectious diseases : an official publication of the Infectious Diseases Society of America 2000; 31: i-ii. |
| Outbreak of Ebola haemorrhagic fever, Uganda, August 2000-January 2001. Releve epidemiologique hebdomadaire / Section d'hygiene du Secretariat de la Societe des Nations = Weekly epidemiological record / Health Section of the Secretariat of the League of Nations 2001; 76: 41-46 |
| Peters, CJ. Viral Hemorrhagic Fevers. Viral Pathogenesis. Ed. Neal Nathanson. Philadelphia: Lippincott-Raven Publishers, 1997: 779-799. |
| Petrovsky, N.. The vaccine renaissance. Human Vaccines 2011; 7: 149-152. |
| Pettineo, C., Aitchison, R., Leikin, S. M., Vogel, S. N., and Leikin, J. B.. Biological and chemical weapons of mass destruction: Updated clinical therapeutic countermeasures since 2003. American Journal of Therapeutics 2009; 16: 35-43. |
| Pourahmad, M., Raoofi, R., Chinikar, S., Ghiasi, S. M., and Ghalyanchi-Langeroudi, A.. Nosocomial transmission of Crimean-Congo hemorrhagic fever in a health care worker, Fars province, Iran. Iranian Journal of Clinical Infectious Diseases 2011; 6: 47-50. |
| Poveda, J.-D., Raccurt, C. P., Le, Fur R., M'Bailara, L., Malvy, J. M. D., Le, Bras M., Saliou, P., and Fleury, H. J. A.. What about the inhibition effect of cholera vaccination on yellow fever immunization? Results of a retrospective study. Bulletin de la Societe de Pathologie Exotique et de ses Filiales 1990; 83: 529-536. |
| Prasad, B. G.. Epidemiological research and its impact on health and medical care. Indian Journal of Medical Research 1972; 60: 501-511. |
| Pre-ART guidelines: Amended November 2004. Southern African Journal of HIV Medicine 2004: 18-31. |
| Prophylactic gamma globulin for prevention of endemic hepatitis. Effects of US gamma globulin upon the incidence of viral hepatitis and other infectious diseases in US Soldiers abroad. Archives of Internal Medicine 1971; 128: 723-738. |
| Public Health Agency of Canada. Marburg Virus: Pathogen safety data sheet- infectious substances. 2011. |
| Public Safety Canada. Consequence management workshop on the terrorist use of nuclear, chemical and biological (NBC) weapons of mass destruction. 1998: 1-179. |
| Punj, P., Nelson, R., Gardiner, S., Lodge, M., Graves, S. R., and Warner, M. S.. A pilgrim's progress: Severe Rickettsia conorii infection complicated by gangrene. Medical Journal of Australia 2013; 198: 629-631. |
| Rai, M. A., Khanani, M. R., Warraich, H. J., Hayat, A., and Ali, S. H.. Crimean-Congo hemorrhagic fever in Pakistan. Journal of Medical Virology 2008; 80: 1004-1006. |
| Rawlinson, T., Siqueira, A. M., Fontes, G., Beltrao, R. P. L., Monteiro, W. M., Martins, M., Silva-Junior, E. F., Mourao, M. P. G., Albuquerque, B., da Alecrim, M. G. C., and Lacerda, M. V. G.. From Haiti to the Amazon: Public Health Issues Related to the Recent Immigration of Haitians to Brazil. PLoS Neglected Tropical Diseases 2014; 8: e2685-1-4. |
| Robb, G. H.. Letter: Use of chloramphenicol. Lancet 1973; 2: 909. |
| Roddy, P., Howard, N., Van Kerkhove, M. D., Lutwama, J., Wamala, J., Yoti, Z., Colebunders, R., Palma, P. P., Sterk, E., Jeffs, B., Van, Herp M., and Borchert, M.. Clinical manifestations and case management of Ebola haemorrhagic fever caused by a newly identified virus strain, Bundibugyo, Uganda, 2007-2008. PLoS ONE [Electronic Resource] 2012; 7: e52986-1-12. |
| Saris prevent cholera infection. Medicine Today 2003; 4: 9-10. |
| Schafer, I. J., McCollum, A. M., Knust, B., Muyembe, J.-J., Shongo, R., Kebela, B., Musa, K., Bergeron, E., Spiropoulou, C., Bird, B., Pukuta, E., Kobinger, G., Adima, F., Nseye, C., Mossoko, M., Mulangu, F., Lampaert, E., Nanclares, C., Cannon, D., Balinandi, S., Tumusiime, A., De La Rosa, O., Alia, M., Rae, Erickson B., Shoemaker, T., Stroher, U., Rollin, P. E., and Nichol, S.. Re-emergence of bundibugyo virus after a five year hiatus-isiro, the democratic republic of the Congo, 2012. American journal of tropical medicine and hygiene 2013; 89: 420. |
| Scrimgeour, E. M., Zaki, A., Mehta, F. R., Abraham, A. K., Al-Busaidy, S., El-Khatim, H., Al-Rawas, S. F. S., Kamal, A. M., and Mohammed, A. J.. Crimean-Congo haemorrhagic fever in Oman. Transactions of the Royal Society of Tropical Medicine and Hygiene 1996; 90: 290-291. |
| Singh, N. and Levi, M. E.. Arenavirus and West Nile virus in solid organ transplantation. American Journal of Transplantation 2013; 13: 361-371. |
| Smego, R. A., Jr., Sarwari, A. R., and Siddiqui, A. R.. Crimean-Congo hemorrhagic fever: prevention and control limitations in a resource-poor country. Clin Infect Dis 2004; 38: 1731-1735. |
| Smith, E. A., Fabiyi, A., Kuteyi, O. E., and Tomori, O.. Epidemiological aspect of the 1976 Pankshin Lassa fever outbreak. Nigerian Medical Journal 1979; 9: 20-22. |
| Sommer, A., Khan, M., and Mosley, W. H.. Efficacy of vaccination of family contacts of cholera cases. Lancet 1973; 1: 1230-1232. |
| Stankus, T.. Ebola and Marburg: Two closely related, highly lethal viruses that cause hemorrhagic fever in Africa and may still be sought by terrorists and rogue states to weaponize and attack anywhere. 2010. |
| Stefanakis, R., Robertson, A. S., Ponder, E. L., and Moree, M.. Analysis of Neglected Tropical Disease Drug and Vaccine Development Pipelines to Predict Issuance of FDA Priority Review Vouchers over the Next Decade. PLoS Neglected Tropical Diseases 2012; 6: e1803-1-7. |
| Steffen, R. and Connor, B. A.. Vaccines in travel health: From risk assessment to priorities. Journal of Travel Medicine 2005; 12: 26-35. |
| Sureau, P. H.. Firsthand clinical observations of hemorrhagic manifestations in Ebola hemorrhagic fever in Zaire. Rev Infect Dis 1989; 11 Suppl 4: S790-S793. |
| Trexler, P. C.. The development of isolators. Postgrad.Med J 1976; 52: 545-549. |
| Tuffs, A.. Experimental vaccine may have saved Hamburg scientist from Ebola fever. BMJ (Clinical research ed.) 2009; 338: b1223. |
| Waldman, R. J., Mintz, E. D., and Papowitz, H. E.. The cure for cholera - Improving access to safe water and sanitation. New England Journal of Medicine 2013; 368: 592-594. |
| Williams, E. H.. 44 contacts of Ebola virus infection--Salisbury. Public Health 1979; 93: 67-75. |
| WHO Headquarters - Library.Geneva Switzerland. Stratégie de prévention et de lutte contre le choléra en Afrique : déclaration lors d' une réunion tenue le 7 mai 1992 Ã  Genève, pendant la Quarante-Cinquième Assemblée mondiale de la Santé, les ministres de la santé des Etats Membres de l' OMS en Afrique et leurs représentants. Relev 1992; 67: 241. |
| Wolfe, M. S.. Protection of travelers. Clinical Infectious Diseases 1997; 25: 177-184. |
| Woodruff, A. W., Monath, T. P., Mahmoud, A. A., Pain, A. K., and Morris, C. A.. Lassa fever in Britain: an imported case. Br Med J 1973; 3: 616-617. |
| World Health Organization. Crimean-Congo haemorrhagic fever. [Internet]. <http://www.who.int/mediacentre/factsheets/fs208/en/>. 2013. |
| Viral haemorrhagic fever surveillance. Weekly Epidemiology Record 1982; 46: 359. |
| **Unclear PPE use among HCWs in Ebola treatment centres/general wards** |
| Ali, F., Saleem, T., Khalid, U., Mehmood, S. F., and Jamil, B.. Crimean-Congo hemorrhagic fever in a dengue-endemic region: lessons for the future. Journal of Infection in Developing Countries 2010; 4: 459-463. |
| Atkinson, B., Latham, J., Chamberlain, J., Logue, C., O'Donoghue, L., Osborne, J., Carson, G., Brooks, T., Carroll, M., Jacobs, M., Hopkins, S., and Hewson, R**.**. Sequencing and phylogenetic characterisation of a fatal Crimean - Congo haemorrhagic fever case imported into the United Kingdom, October 2012. Euro Surveillance: Bulletin Europeen sur les Maladies Transmissibles = European Communicable Disease Bulletin 2012; 17: 1-4. |
| Bitekyerezo, M., Kyobutungi, C., Kizza, R., Mugeni, J., Munyarugero, E., Tirwomwe, F., Twongyeirwe, E., Muhindo, G., Nakibuuka, V., Nakate, M., John, L., Ruiz, A., Frame, K., Priotto, G., Pepper, L., Kabakyenga, J., Baingana, S., and Ledo, D.. The outbreak and control of Ebola viral haemorrhagic fever in a Ugandan medical school. Tropical Doctor 2002; 32: 10-15. |
| Clayton, A. J.. Lassa fever, Marburg and Ebola virus diseases and other exotic diseases: is there a risk to Canada?.Can Med Assoc J 120 (20-1-1979): 146-155. |
| Crowcroft, N. S.. Management of Lassa fever in European countries. [Review] [1 refs]. Euro Surveillance: Bulletin Europeen sur les Maladies Transmissibles = European Communicable Disease Bulletin 2002; 7: 50-52. |
| Crowcroft, N., Brown, D., Gopal, R., and Morgan, D.. Current management of patients with viral haemorrhagic fevers in the United Kingdom. [Review] [5 refs]. Euro Surveillance: Bulletin Europeen sur les Maladies Transmissibles = European Communicable Disease Bulletin 2002; 7: 44-48. |
| Elata, A. T., Karsany, M. S., Elageb, R. M., Hussain, M. A., Eltom, K. H., Elbashir, M. I., and Aradaib, I. E.. A nosocomial transmission of crimean-congo hemorrhagic fever to an attending physician in North Kordufan, Sudan. Virology Journal 2011; 8: 303. |
| Emond, R. T., Evans, B., Bowen, E. T., and Lloyd, G.. A case of Ebola virus infection. British Medical Journal 1977; 2: 541-544. |
| Evans, R. G. and Lawrence, S. J.. Preparing for and responding to bioterrorist attacks: The role of disease management initiatives. Disease Management and Health Outcomes 2006; 14: 265-274. |
| Fusco, F. M., Schilling, S., De, Iaco G., Brodt, H.-R., Brouqui, P., Maltezou, H. C., Bannister, B., Gottschalk, R., Thomson, G., Puro, V., and Ippolito, G**.**. Infection control management of patients with suspected highly infectious diseases in emergency departments: Data from a survey in 41 facilities in 14 European countries. BMC Infectious Diseases 2012; 12: 1-7. |
| Kitching, A., Addiman, S., Cathcart, S., Bischop, L., Krahe, D., Nicholas, M., Coakley, J., Lloyd, G., Brooks, T., Morgan, D., and Turbitt, D.. A fatal case of Lassa fever in London, January 2009.[Erratum appears in Euro Surveill. 2009;14(11):pii/19155]. Euro Surveillance: Bulletin Europeen sur les Maladies Transmissibles = European Communicable Disease Bulletin 2009; 14: 1-3. |
| Lamb, D.. Evaluation of infection control practices during an AE. British Journal of Nursing 15 (25-5-2006): 543-547. |
| Maltezou, H. C., Papa, A., Tsiodras, S., Dalla, V., Maltezos, E., and Antoniadis, A.. Crimean-Congo hemorrhagic fever in Greece: a public health perspective. International Journal of Infectious Diseases 2009; 13: 713-716. |
| Mehrabi, Tavana A.. A review on Crimean-Congo Haemorrhagic Fever in Asia. Journal of Medical Sciences 2006; 6: 901-905. |
| Okware, S. I., Omaswa, F. G., Zaramba, S., Opio, A., Lutwama, J. J., Kamugisha, J., Rwaguma, E. B., Kagwa, P., and Lamunu, M.. An outbreak of Ebola in Uganda. Tropical Medicine and International Health 2002; 7: 1068-1075. |
| Saleem, J., Usman, M., Nadeem, A., Sethi, S. A., and Salman, M.. Crimean-Congo hemorrhagic fever: a first case from Abbottabad, Pakistan. International Journal of Infectious Diseases 2009; 13: e121-e123. |
| Shapira, S. C. and Shemer, J.. Medical management of terrorist attacks. Israel Medical Association Journal 2002; 4: 489-492. |
| Suleiman, M. N., Muscat-Baron, J. M., Harries, J. R., Satti, A. G., Platt, G. S., Bowen, E. T., and Simpson, D. I.. Congo/Crimean haemorrhagic fever in Dubai. An outbreak at the Rashid Hospital. Lancet 1980; 2: 939-941. |
| Tarantola, A., Golliot, F., Astagneau, P., Fleury, L., Brucker, G., and Bouvet, E.. Occupational blood and body fluids exposures in health care workers: four-year surveillance from the Northern France network. Am J Infect Control 2003; 31: 357-363. |
| Wamala, J. F., Lukwago, L., Malimbo, M., Nguku, P., Yoti, Z., Musenero, M., Amone, J., Mbabazi, W., Nanyunja, M., Zaramba, S., Opio, A., Lutwama, J. J., Talisuna, A. O., and Okware, S. I.. Ebola hemorrhagic fever associated with novel virus strain, Uganda, 2007-2008. Emerging Infectious Diseases 2010; 16: 1087-1092. |
| **Infectious agent not relevant (Cholera or other)** |
| D. Abiteboul, F. Lamontagne, I. Lolom, A. Tarantola, J.M. Descamps, E. Bouvet. Incidence des accidents exposant au sang chez le personnel infirmier en France métropolitaine, 1999-2000: résultats d’une enquête multi centrique dans 32 hôpitaux. BEH 2002: 256-259. |
| Enria, D. A. and Pinheiro, F.. Rodent-borne emerging viral zoonosis. Hemorrhagic fevers and hantavirus infections in South America. Infect Dis Clin North Am 2000; 14: 167-84, x. |
| Gbary, A. R., Sossou, R. A., Dossou, J. P., Mongbo, V., and Massougbodji, A.. [The determinants of the low case fatality rate of the cholera epidemic in the Littoral department of Benin in 2008]. [French]. Sante Publique (Vandoeuvre-Les-Nancey) 2011; 23: 345-358. |
| Memish, Z. A., Balkhy, H. H., Francis, C., Cunningham, G., Hajeer, A. H., and Almuneef, M. A.. Alkhumra haemorrhagic fever: case report and infection control details. British Journal of Biomedical Science 2005; 62: 37-39. |
| Sewlall, N.. Arenavirus outbreak with nosocomial transmission: Infection control and the lessons learned. Southern African Journal of Anaesthesia and Analgesia 2011; 17: 54-55. |
| **Infectious agent not relevant (Unclear)** |
| Ngatu, N. R., Phillips, E. K., Wembonyama, O. S., Hirota, R., Kaunge, N. J., Mbutshu, L. H., Perry, J., Yoshikawa, T., Jagger, J., and Suganuma, N.. Practice of universal precautions and risk of occupational blood-borne viral infection among Congolese health care workers. American Journal of Infection Control 2012; 40: 68-70. |
| **Irrelevant Study Design (Narrative Review)** |
| Amundsen, S. B.. Historical analysis of the Ebola virus: prospective implications for primary care nursing today. Clinical Excellence for Nurse Practitioners 1998; 2: 343-351. |
| Bannister, B.. Viral haemorrhagic fevers imported into non-endemic countries: Risk assessment and management. British Medical Bulletin 2010; 95: 193-225. |
| Bausch, D. G., Sprecher, A. G., Jeffs, B., and Boumandouki, P.. Treatment of Marburg and Ebola hemorrhagic fevers: a strategy for testing new drugs and vaccines under outbreak conditions. [Review] [110 refs]. Antiviral Research 2008; 78: 150-161. |
| Bruce, J. and Brysiewicz, P.. Ebola fever: the African emergency. International Journal of Trauma Nursing 2002; 8: 36-41. |
| Casillas, A. M., Nyamathi, A. M., Sosa, A., Wilder, C. L., and Sands, H.. A current review of Ebola virus: pathogenesis, clinical presentation, and diagnostic assessment. Biological Research for Nursing 2003; 4: 268-275. |
| Colebunders, R., Van, Esbroeck M., Moreau, M., and Borchert, M.. Imported viral haemorrhagic fever with a potential for person-to-person transmission: review and recommendations for initial management of a suspected case in Belgium. Acta Clinica Belgica 2002; 57: 233-240. |
| dani, M. and Keshtkar-Jahromi, M.. Crimean-Congo hemorrhagic fever. Archives of Iranian Medicine 2007; 10: 204-214. |
| Ergonul, O.. Crimean-Congo haemorrhagic fever. Lancet Infect Dis 2006; 6: 203-214. |
| Ftika, L. and Maltezou, H. C.. Viral haemorrhagic fevers in healthcare settings. J Hosp Infect 2013; 83: 185-192. |
| Gould, D.. Isolation precautions to prevent the spread of contagious diseases. Nursing Standard 2009; 23: 47-55. |
| Handy, J. M.. Viral haemorrhagic fevers - Implications in intensive care. Current Anaesthesia and Critical Care 2004; 15: 137-142. |
| Idemyor, V.. Lassa virus infection in Nigeria: Clinical perspective overview. Journal of the National Medical Association 2010; 102: 1243-1246. |
| Isaacson, M.. Viral hemorrhagic fever hazards for travelers in Africa. Clin Infect Dis 2001; 33: 1707-1712. |
| Mardani, M. and Pourkaveh, B.. Crimean-Congo hemorrhagic fever. Iranian Journal of Clinical Infectious Diseases 2013; 7: 36-42. |
| Monath, T. P. and Casals, J.. Diagnosis of Lassa fever and the isolation and management of patients. Bull World Health Organ 1975; 52: 707-715. |
| Murthy, S., Keystone, J., and Kissoon, N.. Infections of the Developing World. Critical Care Clinics 2013; 29: 485-507. |
| Nzerue, M. C.. Lassa fever: review of virology, immunopathogenesis, and algorithms for control and therapy. Central African Journal of Medicine 1992; 38: 247-252. |
| Pedrosa, P. B. S. and Cardoso, T. A. O.. Viral infections in workers in hospital and research laboratory settings: A comparative review of infection modes and respective biosafety aspects. International Journal of Infectious Diseases 2011; 15: e366-e376. |
| Pigott, D. C.. Hemorrhagic fever viruses. Critical Care Clinics 2005; 21: 765-783. |
| Raabe, Vanessa N. and Matthias, B.. Infection control during filoviral hemorrhagic fever outbreaks. Journal of Global Infectious Diseases 2012; 4: 69-74. |
| Sarwar, U. N., Sitar, S., and Ledgerwood, J. E.. Filovirus emergence and vaccine development: a perspective for health care practitioners in travel medicine. Travel Medicine & Infectious Disease 2011; 9: 126-134. |
| S.P. Fisher-Hoch. Stringent Precautions are not advisable when caring for patients with viral haemorrhagic fevers. Forum 1993: 7-13. |
| Vorou, R., Pierroutsakos, I. N., and Maltezou, H. C.. Crimean-Congo hemorrhagic fever. Current Opinion in Infectious Diseases 2007; 20: 495-500. |
| Walker, L.. Ebola haemorrhagic fever. Nursing Standard 2001; 15: 40-42. |
| Weber, D. J. and Rutala, W. A.. Risks and prevention of nosocomial transmission of rare zoonotic diseases. Clinical Infectious Diseases 2001; 32: 446-456. |
| Wood, J.. Exotic diseases--communicable, dangerous, and, yes, possible!.Canadian Nurse 1982; 78: 18-22. |
| **Irrelevant Study Design (Protocols and/or non-evidence based clinical practice guidelines/recommendations)** |
| Advisory Committee on Dangerous Pathogens. Management of Hazard Group 4 viral haemorrhagic fevers and similar human infectious diseases of high consequence. 2014: 1-102. |
| Armignacco, O., Lauria, F. N., Puro, V., Macri, G., Petrecchia, A., and Ippolito, G.. The model of response to viral haemorrhagic fevers of the National Institute for Infectious Diseases Lazzaro Spallanzani. Journal of Biological Regulators and Homeostatic Agents 2001; 15: 314-321. |
| Borio, L., Inglesby, T., Peters, C. J., Schmaljohn, A. L., Hughes, J. M., Jahrling, P. B., Ksiazek, T., Johnson, K. M., Meyerhoff, A., O'Toole, T., Ascher, M. S., Bartlett, J., Breman, J. G., Eitzen, E. M., Jr., Hamburg, M., Hauer, J., Henderson, D. A., Johnson, R. T., Kwik, G., Layton, M., Lillibridge, S., Nabel, G. J., Osterholm, M. T., Perl, T. M., Russell, P., Tonat, K., and Working Group on Civilian Biodefense.. Hemorrhagic fever viruses as biological weapons: medical and public health management. JAMA 2002; 287: 2391-2405. |
| Bossi, P., Tegnell, A., Baka, A., Van, Loock F., Hendriks, J., Werner, A., Maidhof, H., Gouvras, G., and Task Force on Biological and Chemical Agent Threats, Public Health Directorate European Commission Luxembourg.. Bichat guidelines for the clinical management of haemorrhagic fever viruses and bioterrorism-related haemorrhagic fever viruses. Euro Surveillance: Bulletin Europeen sur les Maladies Transmissibles = European Communicable Disease Bulletin 2004; 9: E11-E12. |
| Brouqui, P., Puro, V., Fusco, F. M., Bannister, B., Schilling, S., Follin, P., Gottschalk, R., Hemmer, R., Maltezou, H. C., Ott, K., Peleman, R., Perronne, C., Sheehan, G., Siikamaki, H., Skinhoj, P., and Ippolito, G.. Infection control in the management of highly pathogenic infectious diseases: consensus of the European Network of Infectious Disease. Lancet Infect Dis 2009; 9: 301-311. |
| Canadian contingency plan for viral hemorrhagic fevers and other related diseases. Canada Communicable Disease Report 2001; 23 Suppl 1: i-iii. |
| Centers for Disease Control (CDC). Management of patients with suspected viral hemorrhagic fever. [Review] [54 refs]. MMWR - Morbidity & Mortality Weekly Report 1988; 37 Suppl 3: 1-16. |
| Centers for Disease Control and Prevention (CDC). Healthcare Infection Control Practices Advisory Committee (HICPAC) 2007: 1-40. |
| Centers for Disease Control and Prevention (CDC). Recognition of illness associated with the intentional release of a biologic agent. MMWR - Morbidity & Mortality Weekly Report 2001; 50: 893-897. |
| Centers for Disease Control and Prevention (CDC). The public health response to biological and chemical terrorism. Interim planning guidance for state public health officials. 2001: 1-95. |
| Ebolavirus disease (EVD) outbreaks in West Africa. 2014: 1-8. |
| Formenty, P., Epelboin, A., Allarangar, Y., Libama, F., Boumandouki, P., Kone, L., Molamou, A., Gami, N., Mombouli, J. V., Martinez, M. G., and Ngampo, S.. [Training the trainers seminar and analysis of the Ebola virus hemorrhagic fever outbreaks in central Africa from 2001 to 2004. (Brazzaville, Republic of Congo, April 6-8, 2004]. Bulletin de la Societe de Pathologie Exotique 2005; 98: 244-254. |
| Frangoulidis, D. and Meyer, H.. Measures undertaken in the German armed forces field hospital deployed in Kosovo to contain a potential outbreak of Crimean-Congo hemorrhagic fever. Military Medicine 2005; 170: 366-369. |
| From the Centers for Disease Control and Prevention. Update: management of patients with suspected viral hemorrhagic fever--United States. JAMA 1995; 274: 374-375. |
| Garner, J. S.. Guideline for isolation precautions in hospitals. The Hospital Infection Control Practices Advisory Committee. Infection control and hospital epidemiology : the official journal of the Society of Hospital Epidemiologists of America 1996; 17: 53-80. |
| Hall, R. C., Hall, R. C., and Chapman, M. J.. The 1995 Kikwit Ebola outbreak: lessons hospitals and physicians can apply to future viral epidemics. General Hospital Psychiatry 2008; 30: 446-452. |
| Heath Department of Western Australia. Contingency plan for cases of suspected viral hemorrhagic fever within Western Australia. 2007: 1-26. |
| Infection control guidelines for management of suspected or confirmed ebola virus disease (EVD). 2014: 1-10. |
| Isouard, N.. Infection control in health care settings. Guidelines for MSF projects. 2006: 1-382. |
| Lloyd, E. S., Zaki, S. R., Rollin, P. E., Tshioko, K., Bwaka, M. A., Ksiazek, T. G., Calain, P., Shieh, W.-J., Konde, M. K., Verchueren, E., Perry, H. N., Manguindula, L., Kabwau, J., Ndambi, R., and Peters, C. J.. Long-term disease surveillance in Bandundu region, Democratic Republic of the Congo: A model for early detection and prevention of Ebola hemorrhagic fever. Journal of Infectious Diseases 1999; 179: S274-S280. |
| Nkoghe, D., Formenty, P., Nnegue, S., Mve, M. T., Hypolite, I., Leonard, P., Leroy, E., and Comite International de Coordination Technique et Scientifique.. [Practical guidelines for the management of Ebola infected patients in the field]. Medecine Tropicale 2004; 64: 199-204. |
| OSHA's bloodborne pathogens standard: analysis and recommendations. Health Devices 1993; 22: 35-92. |
| Paverd, N.. Crimean-Congo haemorrhagic fever. A protocol for control and containment in a health care facility--Part 1. Nursing RSA Verpleging 1988; 3: 22-29. |
| Public Health England. Summary Guidance for acute trust staff: Identifying and managing patients who require assessment for Ebola virus disease. 2014: 1-3. |
| Public Health Ontario. Infection Prevention and Control Guidance for Patients With Suspected or Confirmed Ebola Virus Disease (EVD) in Ontario Health Care Settings. 2014: 1-23. |
| Sardesai, A. M., Brown, N. M., and Menon, D. K.. Deliberate release of biological agents. Anaesthesia 2002; 57: 1067-1082. |
| Siegel, J. D., Rhinehart, E., Jackson, M., and Chiarello, L.. 2007 Guideline for Isolation Precautions: Preventing Transmission of Infectious Agents in Health Care Settings. American Journal of Infection Control 2007; 35: S65-S164. |
| Smith, P. W., Anderson, A. O., Christopher, G. W., Cieslak, T. J., Devreede, G. J., Fosdick, G. A., Greiner, C. B., Hauser, J. M., Hinrichs, S. H., Huebner, K. D., Iwen, P. C., Jourdan, D. R., Kortepeter, M. G., Landon, V. P., Lenaghan, P. A., Leopold, R. E., Marklund, L. A., Martin, J. W., Medcalf, S. J., Mussack, R. J., Neal, R. H., Ribner, B. S., Richmond, J. Y., Rogge, C., Roselle, G. A., Rupp, M. E., Sambol, A. R., Schaefer, J. E., Sibley, J., Streifel, A. J., Von Essen, S. G., and Warfield, K. L.. Designing a biocontainment unit to care for patients with serious communicable diseases: A consensus statement. Biosecurity and Bioterrorism 2006; 4: 351-365. |
| Valenti, W. M., Hruska, J. F., Menegus, M. A., and Freeburn, M. J.. Nosocomial viral infections: III. Guidelines for prevention and control of exanthematous viruses, gastroenteritis viruses, picornaviruses, and uncommonly seen viruses. Infection Control 1981; 2: 38-49. |
| Wirtz, A., Niedrig, M., and Fock, R.. Management of patients in Germany with suspected viral haemorrhagic fever and other potentially lethal contagious infections. Euro Surveillance: Bulletin Europeen sur les Maladies Transmissibles = European Communicable Disease Bulletin 2002; 7: 36-42. |
| Woodruff, A. W.. Handling patients with suspected Lassa fever entering Great Britain. Bull World Health Organ 1975; 52: 717-721. |
| World Health Organization. WHO recommended strategies for the prevention and control of communicable diseases. 2001: 1-190. |
| **Not of interest (editorials, commentaries)** |
| European Centre for Disease Prevention and Control. Rapid Risk Assessment: Outbreak of Ebola virus disease in West Africa. 2014: 1-13. |
| **Other reason: Intent not to provide data on use of PPE (knowledge and values of PPE use).** |
| Sheikh, N. S., Sheikh, A. S., and Sheikh, A. A.. Knowledge, attitude and practices regarding Crimean-Congo haemorrhagic fever among healthcare workers in Balochistan. Journal of Ayub Medical College, Abbottabad: JAMC 2004; 16: 39-42. |
