# Supplementary material for: Effectiveness of Personal Protective Equipment for Healthcare Workers Caring for Patients with Filovirus Disease: A Rapid Review
Source: PLoS One. 2015 Oct 9;10(10):e0140290. doi: 10.1371/journal.pone.0140290 (PMC4599797; doi:10.1371/journal.pone.0140290)
Supplement: S1 Table — (DOCX) [file pone.0140290.s005.docx]

S1 Table. Study characteristics table of non-comparative studies of healthcare workers wearing gloves

| **Study (year of publication)**  **Location**  **Setting**  **Sources of support** | **Year of outbreak** | **Surveillance details**  **Number of participants**  **Type of HCWs** | **PPE protocol**  **Protocol violations (if reported)** | **Outcomes and results** |
| --- | --- | --- | --- | --- |
| **Marburg Virus Disease** | | | | |
| Martini, GA. (1969)  Marburg, Germany  University of Marburg, Medical Department  NR | 1967 | NA (CR)  Total number of HCW contacts not reported. PPE described for one HCW  Doctor (nurse also contracted disease nosocomially but PPE use not described) | Doctor who contracted disease wore rubber gloves (use of additional PPE not described) | **Virus transmission** – Virus transmission to doctor. Diagnosis according to symptoms  **Needle-stick injury -**  Doctor had a percutaneous exposure to infected blood after needle pricked his gloves |
| **Crimean-Congo Hemorrhagic Fever** | | | | |
| Athar, MN. (2005)  Rawalpindi, Pakistan  Hospital, tertiary care center  NR | 2002 | Unclear  154^a^ HCW contacts (PPE use reported for only one HCW)  NR for all HCWs. HCW who contracted disease was an intern. | Intern used latex gloves but no face mask or shield. | **Virus transmission –** Virus transmission to intern. Diagnosed according to symptoms, serology (IgM and IgG positive; ELISA), and RT-PCR confirmation. |
| Altaf, A. (1998)  Quetta and Karachi, Pakistan  Three hospitals (one of which was a private hospital)  USAMRIID provided reagents for ELISA | 1994 | Contacts interviewed and serologically tested  42^a^ HCW contacts (PPE use described for 28)  Assistant and operating surgeons, theatre assistant, anaesthesiologist, nurses, gastroenterologists, assistants, internists, intern, faculty members | PPE use varied across HCWs:  Glove use (other PPE not described) was specifically noted for 7 HCW. Some HCWs did not wear gloves or glove use was unclear.    21 HCWs in one hospital wore gloves, aprons and face masks (corresponding data reported in Table 2) | **Virus transmission -**  Gloves (other PPE not described): 2/7 HCW tested positive for Crimean-Congo IgG/IgM antibodies (ELISA) and developed disease symptoms  **Needle-stick injuries -**  reported by one HCW who wore gloves (tested positive for antibodies)  **Glove perforation** - reported by 2 HCWs. One tested positive for antibodies (tore gloves twice; possible percutaneous contact with blood) and the other negative. |
| Naderi, HR. (2011)  Mashhad, Iran  Hospital, Department of Gynaecology  NR | 2009 | NA (CR)  4 (PPE described for only 2 HCWs)  Carers | One carer wore perforated gloves with no further description of PPE  The other carer always used intact gloves but did not always use a face shield or surgical mask and eye protection (corresponding data reported in Table 4) | **Virus transmission –** carer developed symptoms and diagnosis was virologically confirmed using RT-PCR |

^a^HCW may include personnel that did not provide direct patient care.

Abbreviations: ELISA=enzyme-linked immunosorbent assay; HCW=healthcare worker; IgG=immunoglobulin G; IgM=immunoglobulin M; NA=not applicable; NR=not reported; PPE=personal protective equipment; RT-PCR=reverse transcription polymerase chain reaction; USAMRIID=United States Army Medical Research Institute for Infectious Diseases

**References**

Martini GA. Marburg agent disease: in man. Trans R Soc Trop Med Hyg 1969;63(3):295-302. [PMID: 5815873]

Athar MN, Khalid MA, Ahmad AM, Bashir N, Baqai HZ, Ahmad M, et al. Crimean-Congo hemorrhagic fever outbreak in Rawalpindi, Pakistan, February 2002: contact tracing and risk assessment. Am J Trop Med Hyg 2005 Apr;72(4):471-3. [PMID: 15827289]

Altaf A, Luby S, Ahmed AJ, Zaidi N, Khan AJ, Mirza S, et al. Outbreak of Crimean-Congo haemorrhagic fever in Quetta, Pakistan: contact tracing and risk assessment. Trop Med Int Health 1998 Nov;3(11):878-82. [PMID: 9855399]

Naderi HR, Sarvghad MR, Bojdy A, Hadizadeh MR, Sadeghi R, Sheybani F. Nosocomial outbreak of Crimean-Congo haemorrhagic fever. Epidemiol Infect 2011 Jun;139(6):862-6. [PMID: 20800007]
