# Supplementary material for: Effectiveness of Personal Protective Equipment for Healthcare Workers Caring for Patients with Filovirus Disease: A Rapid Review
Source: PLoS One. 2015 Oct 9;10(10):e0140290. doi: 10.1371/journal.pone.0140290 (PMC4599797; doi:10.1371/journal.pone.0140290)
Supplement: S2 Table — (DOCX) [file pone.0140290.s006.docx]

**S2 Table. Study characteristics table of non-comparative studies of healthcare workers wearing gloves, gowns/aprons, and masks**

| **Study (year of publication)**  **Location**  **Setting**  **Sources of support** | **Year of outbreak** | **Surveillance details**  **Number of participants**  **Type of HCWs** | **PPE protocol**  **Protocol violations (if reported)** | **Outcomes and results** |
| --- | --- | --- | --- | --- |
| **Ebola Virus Disease** | | | | |
| Formenty, P. (1999) [1]  Basel, Switzerland  University hospital ("single, double-door isolation room with negative pressure")  NR | 1994 | Contacts interviewed and serologically tested  40^†^  Hospital (and laboratory staff). Not further described. | All HCWs wore high-quality gloves, gowns, and dust-and-mist masks.  Infection control practices according to Centers for Disease Control and Prevention. | **Virus transmission –** No transmission according to serologic analysis (no antibodies detected by ELISA or IFA). |
| Shoemaker, T. (2012) [2]  Luwero District, Uganda  Military hospital  Support provided by National Task Force on Ebola, Uganda Virus Research Institute, United States Agency for International Development, Emerging Pandemic Threats, PREDICT and RESPOND, African Field Epidemiology Network, and United Nations, Children's Fund. Support provided by WHO and CDC-Uganda was also noted. | 2011 | Unclear  12^†^  NR | Gloves, gowns, masks (unclear if other equipment was used) | **Virus transmission –** No transmission (method of confirmation for HCWs not reported) |
| **Crimean-Congo Hemorrhagic Fever** | | | | |
| Papa, A. (2002) [3]  Kosovo, Yugoslavia  Hospital  NR | 2001 | Follow-up duration NR; contacts identified and asked to inform physician if symptoms developed  NR  NR | “All personnel wore protective clothing, such as gowns, masks, and gloves" | **Virus transmission –** No transmission (according to symptomology) |
| van de Wal, BW. (1985) [4]  South Africa  Medical ward of hospital dedicated to care of CCHF patients  NR | 1984 | Followed daily or every other day until end of incubation period  Unclear (459 contacts - proportion of HCWs not reported. 217 of 459 contacts screened in hospital)  NR | "… full theatre clothes, including masks, gowns, and gloves" | **Virus transmission -** Only one case developed following implementation of infection control measures (nurse injured during skin biopsy of a secondary case). Remaining cases (n=6; unclear if all were HCWs) were contacts of index case and apparently contracted disease prior to infection control measures.  **Needle-stick injury –** Nine Needle-stick injuries occurred (includes one injury during skin biopsy procedure). Approximately two occurred post implementation of infection control measures. Unclear if remaining injuries occurred prior to protocol implementation, |
| Altaf, A. (1998) [5]  Quetta and Karachi, Pakistan  Three hospitals (one of which was a private hospital)  USAMRIID provided reagents for ELISA | 1994 | Contacts interviewed and serologically tested  42^†^ HCW contacts (PPE use described for 28)  Assistant and operating surgeons, theatre assistant, anaesthesiologist, nurses, gastroenterologists, assistants, internists, intern, faculty members | PPE use varied across HCWs:  21 nurses working in Aga Khan University Hospital wore gloves, aprons and face masks. This protocol was implemented 18 hours after admitting patient. Prior to implementation, nurses were exposed to infected patients without PPE, however, they had no physical contact with patient.  Glove use (other PPE not described) was specifically noted for 7 HCW (corresponding data in S1 Table). Some HCWs did not wear gloves or glove use was unclear. | **Virus transmission -** 0/21 HCWs tested positive for antibodies. All 21 had no physical contact with infected patients. |
| Gozel, MG. (2013) [6]  Sivas, Turkey  University Education and Research Hospital - a tertiary care reference hospital  Supported by Scientific Research Project Fund of Cumhuriyet University | NA | Serological assessment of HCWs exposed to CCHF cases over a ten-year period  104 (excluding laboratory workers and housekeeping staff)  Nurses (30%), physicians (24·7%) | 95·2% wore gowns, 80·8% wore gloves, 49% wore masks | **Antibody prevalence -** 1/104 positive for CCHF IgG (ELISA) – this nurse had visited endemic region. 0/104 positive for CCHF IgM. 0/4 high-risk contacts developed disease symptoms or tested positive for antibodies.  **Needle-stick injury –** reported by two HCWs |
| Ergonul, O. (2007) [7]  Ankara, Turkey  Education and Research Hospital - a referral tertiary-care community hospital  NR | NA | Serological assessment of HCWs exposed to CCHF cases over a one-year period  41 (excluding laboratory workers and housekeeping staff)  Nurses (37%), residents (18%), specialists (11%) | Use of general PPE precautions was suggested (i.e. glove use during phlebotomy and use of masks and gowns to protect against body fluids)  Of 62 HCWs (including laboratory workers and housekeeping staff), 80% reported use of gloves and 11% reported use of masks | **Antibody prevalence –**  None of the HCWs exposed to CCHF patients tested positive for IgM or IgG antibodies (ELISA);  **Needle-stick injury -** No needle-stick injury reported |
| **Lassa fever** | | | | |
| Monath, TP. (1973) [8]  Zorzor, Liberia  Hospital, Obstetrical ward  NR | 1972 | Unclear  26  OB ward staff: Graduate nurses, midwives, aides (heavy patient care responsibilities), student nurses. | Full gown, glove and mask isolation implemented 28 days after admission of index case to obstetrical ward | **Virus transmission –** Seven hospital staff developed disease (unclear whether disease developed prior or post PPE protocol implementation). Serological and/or virological confirmation for all 7 HCWs. |
| Helmick, CG. (1986) [9]  Segbwerna, Panguma, and Kenema Sierra Leone  Three hospitals - 100 to 200 beds in each  NR | NA (HCWs assessed between 1972-1983 for prospective cohort analysis); | Serologic assessment conducted at irregular intervals between 1972 and 1977 or 1978 and 1983; median observation was 633 days [range: 34-3905])  Analysis of virus transmission: 305^†^ hospital staff provided two to six blood specimens over median of 633 days (range: 34-3905).  Analysis of seroprevalence: 496^†^ hospital staff  Hospital staff included office workers, drivers, laboratory workers, cleaners, plumbers, launderers, nurses, physicians. | Gloves, gowns, masks recommended but adherence unclear (possibly varied) | **Virus transmission -** 48/305 (16%) hospital staff showed serological evidence of infection  **Antibody prevalence (IFA) –** 108/496 (22%) |
| Fisher-Hoch, SP (1985) [10]  Sierra Leone  Hospital A (place of employment of case)  Hospital B (Lassa fever referral centre; 200 beds)  World Health Organization | 1985 | Serologic assessment immediately and after four weeks  Unclear (50 contacts - proportion of HCWs unclear). Attending medical and nursing staff plus social and medical contacts: n=20; casual contacts (all of which were employed in hospital A and had no direct contact with the case): n=30  NR | Hospital A: Prior to outbreak, "barrier techniques...had been relaxed". Full PPE protocol implemented following detection of outbreak (gloves, gowns, masks).  Hospital B: Full barrier nursing at all times (disposable gloves, paper masks, gowns)  **Instruments, masks, gloves, and disposable items cleaned with bleach (dilution of 1:10). Larger items were burnt following cleaning. | **Virus transmission:**  *Hospital A (delayed implementation of full PPE protocol - gloves, gowns, masks) and Hospital B (full barrier nursing at all times - disposable gloves, paper masks, gowns) contacts:* 0/20 attending medical and nursing staff and close social and medical contacts were seropositive (IFA) and no seroconversions occurred.  *Hospital A (delayed implementation of full PPE protocol - gloves, gowns, masks):* 9/30 casual contacts tested positive for Lassa virus antibodies with initial IFA titres ranging from 16 to 256. Further three seroconverted to titre of 64. None of these developed symptoms. |

^†^HCW may include personnel that did not provide direct patient care.

Abbreviations: CCHF=Crimean-Congo Hemorrhagic Fever; CDC=Centers for Disease Control and Prevention; ELISA=enzyme-linked immunosorbent assay; HCW=healthcare worker; IFA=indirect fluorescent antibody; IgG=immunoglobulin G; IgM=immunoglobulin M; NA=not applicable; NR=not reported; PPE=personal protective equipment; RT-PCR=reverse transcription polymerase chain reaction; USAMRIID=United States Army Medical Research Institute for Infectious Diseases; WHO=World Health Organization

**References**

1. Formenty P, Hatz C, Le GB, Stoll A, Rogenmoser P, Widmer A. Human infection due to Ebola virus, subtype Cote d'Ivoire: clinical and biologic presentation. J Infect Dis 1999 Feb;179 Suppl 1:S48-S53. [PMID: 9988164] 2. Athar MN, Khalid MA, Ahmad AM et al. Crimean-Congo hemorrhagic fever outbreak in Rawalpindi, Pakistan, February 2002: contact tracing and risk assessment. Am J Trop Med Hyg 2005; 72(4):471-473.

2. Shoemaker T, MacNeil A, Balinandi S, Campbell S, Wamala JF, McMullan LK, et al. Reemerging Sudan Ebola virus disease in Uganda, 2011. Emerg Infect Dis 2012 Sep;18(9):1480-3. [PMID: 22931687]

3. Papa A, Bozovi B, Pavlidou V, Papadimitriou E, Pelemis M, Antoniadis A. Genetic detection and isolation of crimean-congo hemorrhagic fever virus, Kosovo, Yugoslavia. Emerg Infect Dis 2002 Aug;8(8):852-4. [PMID: 12141973]

4. van de Wal BW, Joubert JR, van Eeden PJ, King JB. A nosocomial outbreak of Crimean-Congo haemorrhagic fever at Tygerberg Hospital. Part IV. Preventive and prophylactic measures. S Afr Med J 1985 Nov 9;68(10):729-32. [PMID: 2414853]

5. Altaf A, Luby S, Ahmed AJ, Zaidi N, Khan AJ, Mirza S, et al. Outbreak of Crimean-Congo haemorrhagic fever in Quetta, Pakistan: contact tracing and risk assessment. Trop Med Int Health 1998 Nov;3(11):878-82. [PMID: 9855399]

6. Gozel MG, Dokmetas I, Oztop AY, Engin A, Elaldi N, Bakir M. Recommended precaution procedures protect healthcare workers from Crimean-Congo hemorrhagic fever virus. Int J Infect Dis 2013;17(11):1046-50.

7. Ergonul O, Zeller H, Celikbas A, Dokuzoguz B. The lack of Crimean-Congo hemorrhagic fever virus antibodies in healthcare workers in an endemic region. Int J Infect Dis 2007;11(1):48-51.

8. Monath TP, Mertens PE, Patton R, Moser CR, Baum JJ, Pinneo L, et al. A hospital epidemic of Lassa fever in Zorzor, Liberia, March-April 1972. Am J Trop Med Hyg 1973 Nov;22(6):773-9. [PMID: 4745236]

9. Helmick CG, Webb PA, Scribner CL, Krebs JW, McCormick JB. No evidence for increased risk of Lassa fever infection in hospital staff. Lancet 1986 Nov 22;2(8517):1202-5. [PMID: 2877335]

10. Fisher-Hoch SP, Price ME, Craven RB, Price FM, Forthall DN, Sasso DR, et al. Safe intensive-care management of a severe case of Lassa fever with simple barrier nursing techniques. Lancet 1985 Nov 30;2(8466):1227-9. [PMID: 2866301]
