# Supplementary material for: Effectiveness of Personal Protective Equipment for Healthcare Workers Caring for Patients with Filovirus Disease: A Rapid Review
Source: PLoS One. 2015 Oct 9;10(10):e0140290. doi: 10.1371/journal.pone.0140290 (PMC4599797; doi:10.1371/journal.pone.0140290)
Supplement: S3 Table — (DOCX) [file pone.0140290.s007.docx]

**S3 Table. Study characteristics of non-comparative studies of healthcare workers wearing gloves, mask or face shield, and eye protection**

| **Study (year of publication)**  **Location**  **Setting**  **Sources of support** | **Year of outbreak** | **Surveillance details**  **Number of participants**  **Type of HCWs** | **PPE protocol**  **Protocol violations (if reported)** | **Outcomes and results** |
| --- | --- | --- | --- | --- |
| **Crimean-Congo Hemorrhagic Fever** | | | | |
| Naderi, HR. (2011) [1]  Mashhad, Iran  Hospital, Department of Gynaecology  NR | 2009 | NA (CR)  4 (PPE described for only 2 HCWs)  Carers | One carer always used intact gloves but did not always use a face shield or surgical mask and eye protection  The other carer wore perforated gloves with no further description of PPE  (corresponding data reported in Table 1) | **Virus transmission –** carer developed symptoms and diagnosis was virologically confirmed using RT-PCR |

†HCW may include personnel that did not provide direct patient care.

Abbreviations: HCW=healthcare worker; NR=not reported; PPE=personal protective equipment; RT-PCR=reverse transcription polymerase chain reaction

**References**

1. Naderi HR, Sarvghad MR, Bojdy A et al. Nosocomial outbreak of Crimean-Congo haemorrhagic fever. Epidemiol Infect 2011; 139(6):862-866.
