# Supplementary material for: Effectiveness of Personal Protective Equipment for Healthcare Workers Caring for Patients with Filovirus Disease: A Rapid Review
Source: PLoS One. 2015 Oct 9;10(10):e0140290. doi: 10.1371/journal.pone.0140290 (PMC4599797; doi:10.1371/journal.pone.0140290)
Supplement: S4 Table — (DOCX) [file pone.0140290.s008.docx]

**S4 Table. Study characteristics of non-comparative studies of healthcare workers wearing gloves, masks, gowns, and shoe covers**

| **Study (year of publication)**  **Location**  **Setting**  **Sources of support** | **Year of outbreak** | **Surveillance details**  **Number of participants**  **Type of HCWs** | **PPE protocol**  **Protocol violations (if reported)** | **Outcomes and results** |
| --- | --- | --- | --- | --- |
| **Ebola Virus Disease** | | | | |
| Kalongi, Y. (1999) [1]  Kinshasa, Democratic Republic of Congo  Private clinic  NR | 1995 | Unclear  NR (>50 contacts exposed prior to implementation of PPE protocol)  NR | Upon admission of case: 'Standard barrier-nursing precautions' (not further defined);  10 days after admission: 'Enhanced barrier-nursing' initiated (not clearly described but use of gloves, gowns, masks, and shoe covers noted)  PPE removed in anteroom (also used for handwashing and disinfection of non-disposable items using bleach). Disposable items were incinerated. | **Virus transmission -** No secondary transmission of disease.  No symptoms developed among 50 contacts (proportion of HCWs NR) exposed prior to PPE implementation.  All 53 contacts (proportion of HCWs NR) tested for Ebola virus antibodies (IgM and IgG) were negative. |
| **Suspected viral hemorrhagic fever** | | | | |
| Loeb, M. (2003) [2]  Canada  Tertiary care  university hospital (ER, ICU)  NR | 2001 | Self-reported temperature [twice daily] and symptoms  79 (stratified by risk level) | After suspicion of VHF: fluid-resistant gowns and gloves, fluid-resistant masks (filtered 0.03 µm, negative air flow), shoe covers;  Patient kept in isolation and access restricted to HCW. Equipment used on patient did not leave the room. Body secretions, excretions, and fluids disinfected with sodium hypochlorite. Room cleaned with quaternary ammonia compound. Disposable items double-bagged and incinerated. | No outcomes reported. VHF ruled out as possible diagnosis. |

†HCW may include personnel that did not provide direct patient care.

Abbreviations: ER=emergency room; HCW=healthcare worker; ICU=intensive care unit; IgG=immunoglobulin G; IgM=immunoglobulin M; NR=not reported; PPE=personal protective equipment; VHF=viral hemorrhagic fever

**References**

1. Kalongi Y, Mwanza K, Tshisuaka M et al. Isolated case of Ebola hemorrhagic fever with mucormycosis complications, Kinshasa, Democratic Republic of the Congo. J Infect Dis 1999; 179 Suppl 1:S15-S17.

2. Loeb M, MacPherson D, Barton M et al. Implementation of the Canadian contingency plan for a case of suspected viral hemorrhagic fever. Infect Control Hosp Epidemiol 2003; 24(4):280-283.
