# Supplementary material for: Effectiveness of Personal Protective Equipment for Healthcare Workers Caring for Patients with Filovirus Disease: A Rapid Review
Source: PLoS One. 2015 Oct 9;10(10):e0140290. doi: 10.1371/journal.pone.0140290 (PMC4599797; doi:10.1371/journal.pone.0140290)
Supplement: S5 Table — (DOCX) [file pone.0140290.s009.docx]

**S5 Table. Study characteristics of non-comparative studies of healthcare workers wearing gloves, masks, gowns, caps, and shoe covers**

| **Study (year of publication)**  **Location**  **Setting**  **Sources of support** | **Year of outbreak** | **Surveillance details**  **Number of participants**  **Type of HCWs** | **PPE protocol**  **Protocol violations (if reported)** | **Outcomes and results** |
| --- | --- | --- | --- | --- |
| **Lassa fever** | | | | |
| Zweighaft, RM.(1977) [1]  Repatriated to Washington District of Columbia, USA  University Medical Centre (private room with negative air pressure)  NR | 1976 | Surveillance conducted over 21 day incubation period and involved daily self-reporting of temperature and symptoms  87†  NR | High-efficiency biologic filter mask (Custom Comfo Respirator with type H ultra filter cartridge), disposable paper gown, cap, booties and double gloves | **Virus transmission -** No secondary transmission of disease. No symptoms developed among all 12 high risk and 75 low risk hospital contacts. 29 of 33 high risk contacts (proportion of HCW tested unknown) did not have antibodies for Lassa fever. |

†HCW may include personnel that did not provide direct patient care.

Abbreviations: HCW=healthcare worker; NR=not reported; PPE=personal protective equipment

**References**

1. Zweighaft RM, Fraser DW, Hattwick MA et al. Lassa fever: response to an imported case. N Engl J Med 1977; 297(15):803-807.
