# Supplementary material for: Effectiveness of Personal Protective Equipment for Healthcare Workers Caring for Patients with Filovirus Disease: A Rapid Review
Source: PLoS One. 2015 Oct 9;10(10):e0140290. doi: 10.1371/journal.pone.0140290 (PMC4599797; doi:10.1371/journal.pone.0140290)
Supplement: S6 Table — (DOCX) [file pone.0140290.s010.docx]

**S6 Table. Study characteristics of non-comparative studies of healthcare workers wearing gloves, masks, gowns, and respirators**

| **Study (year of publication)**  **Location**  **Setting**  **Sources of support** | **Year of outbreak** | **Surveillance details**  **Number of participants**  **Type of HCWs** | **PPE protocol**  **Protocol violations (if reported)** | **Outcomes and results** |
| --- | --- | --- | --- | --- |
| **Marburg Virus Disease** | | | | |
| Smith, DH. (1982) [1]  Nairobi, Kenya    Hospital (single room in ICU)  Support provided by South African Institute of Medical Research, National Institute of Virology (South Africa), Special Pathogens Section of the CDC, South African Institute of Medical Research and South African Department of Health, WHO, and Kenyan Ministry of Health | 1980 | Contacts quarantined for 14 days from last contact; self-reporting of temperature [twice daily] and symptoms  67† (excluding remote contacts who were not involved in patient care)  Nurses, doctors, attendants, laboratory staff, surgical team | From admission to day 17 of illness (approx. 14 days in-hospital): Unclear  On 17th day of illness (approx. 14th day of hospitalization): Disposable gloves, masks, gowns  Within 48 hours of PPE implementation: Vickers positive-pressure respirators added | **Virus transmission –** No confirmed transmission (symptom and serologic assessment) |

†HCW may include personnel that did not provide direct patient care.

Abbreviations: CDC=Centers for Disease Control and Prevention; HCW=healthcare worker; ICU=intensive care unit; NR=not reported; PPE=personal protective equipment; WHO=World Health Organization

**References**

1. Smith DH, Johnson BK, Isaacson M et al. Marburg-virus disease in Kenya. Lancet 1982; 1(8276):816-820.
