# Supplementary material for: Effectiveness of Personal Protective Equipment for Healthcare Workers Caring for Patients with Filovirus Disease: A Rapid Review
Source: PLoS One. 2015 Oct 9;10(10):e0140290. doi: 10.1371/journal.pone.0140290 (PMC4599797; doi:10.1371/journal.pone.0140290)
Supplement: S7 Table — (DOCX) [file pone.0140290.s011.docx]

**S7 Table. Study characteristics of non-comparative studies of healthcare workers wearing gloves, masks, gowns, face visors, respirators, and shoes**

| **Study (year of publication)**  **Location**  **Setting**  **Sources of support** | **Year of outbreak** | **Surveillance details**  **Number of participants**  **Type of HCWs** | **PPE protocol**  **Protocol violations (if reported)** | **Outcomes and results** |
| --- | --- | --- | --- | --- |
| **Viral Hemorrhagic Fever (unspecified)** | | | | |
| Foberg, U. (1991) [1]  Linkoping, Sweden  University hospital (Department of Infectious Diseases and ICU)  NR | 1990 | Unclear  44 (not including laboratory personnel)  Physicians, volunteer medical personnel | Upon hospitalization: Unclear  Upon admission to ICU: Gloves, masks, gowns  One day later: Face visors added  One day later: Disposable gloves, gowns, masks, and shoes added  Six days after admission: Military gas masks or respirators with HEPA-filters and hoods added  Timing unclear: Military gas masks or respirators with HEPA-filters and hoods replaced with transparent hood with battery-drived HEPA-filtered air blower  During fifth week of illness: Return to simple barrier nursing | **Virus transmission –** No secondary transmission even among those with close contact prior to implementation of strict PPE protocol (method of confirmation NR)  **Needle-stick injury –** 3 HCWs experienced needle-stick injury or cuts |

†HCW may include personnel that did not provide direct patient care.

Abbreviations: HCW=healthcare worker; HEPA=high-efficiency particulate air; ICU=intensive care unit; NR=not reported; PPE=personal protective equipment

**References**

1. Foberg U, Fryden A, Isaksson B et al. Viral haemorrhagic fever in Sweden: experiences from management of a case. Scand J Infect Dis 1991; 23(2):143-151.
