# Supplementary material for: Effectiveness of Personal Protective Equipment for Healthcare Workers Caring for Patients with Filovirus Disease: A Rapid Review
Source: PLoS One. 2015 Oct 9;10(10):e0140290. doi: 10.1371/journal.pone.0140290 (PMC4599797; doi:10.1371/journal.pone.0140290)
Supplement: S8 Table — (DOCX) [file pone.0140290.s012.docx]

**S8 Table. Study characteristics of non-comparative studies of healthcare workers wearing gloves, gowns, eye protection, respirators, and boots**

| **Study (year of publication)**  **Location**  **Setting**  **Sources of support** | **Year of outbreak** | **Surveillance details**  **Number of participants**  **Type of HCWs** | **PPE protocol**  **Protocol violations (if reported)** | **Outcomes and results** |
| --- | --- | --- | --- | --- |
| **Ebola Virus Disease** | | | | |
| Khan, AS. (1999) [1]  Kikwit, Democratic Republic of Congo  Two hospitals (general and maternity)  WHO, CDC, Institut Pasteur, Epicentre, Médecins Sans Frontières, Institute of Tropical Medicine, and the International Red Cross provided outbreak support | 1995 | Unclear  NR  NR | Initial barrier nursing protocol: Unclear  Extended PPE protocol: Not clearly described. Use of disposable HEPA filter mask and eye protection, gloves, gowns, boots was described for one HCW. | **Virus transmission -** One HCW was infected after implementation of extended PPE protocol. Unclear if serologically confirmed  **Inadvertent touching of face –** Possible touching of face with soiled glove reported by one HCW who contracted the virus |

†HCW may include personnel that did not provide direct patient care.

Abbreviations: CDC=Centers for Disease Control and Prevention; HCW=healthcare worker; HEPA=high-efficiency particulate air; NR=not reported; PPE=personal protective equipment; WHO=World Health Organization

**References**

1. Khan AS, Tshioko FK, Heymann DL et al. The reemergence of Ebola hemorrhagic fever, Democratic Republic of the Congo, 1995. Commission de Lutte contre les Epidemies a Kikwit. J Infect Dis 1999; 179 Suppl 1:S76-S86.
