# Supplementary material for: Effectiveness of Personal Protective Equipment for Healthcare Workers Caring for Patients with Filovirus Disease: A Rapid Review
Source: PLoS One. 2015 Oct 9;10(10):e0140290. doi: 10.1371/journal.pone.0140290 (PMC4599797; doi:10.1371/journal.pone.0140290)
Supplement: S9 Table — (DOCX) [file pone.0140290.s013.docx]

**S9 Table. Study characteristics of non-comparative studies of healthcare workers wearing gloves, gowns, respirators or goggles plus mask, scalp protection, and shoe covers**

| **Study (year of publication)**  **Location**  **Setting**  **Sources of support** | **Year of outbreak** | **Surveillance details**  **Number of participants;**  **Type of HCWs** | **PPE protocol**  **Protocol violations (if reported)** | **Outcomes and results** |
| --- | --- | --- | --- | --- |
| **Ebola Virus Disease** | | | | |
| International Commission, (1978) [1];  Kinshasa, Zaire (Democratic Republic of Congo) and surrounding areas- Bumba Zone  Hospital;  Participation of several organizations in outbreak efforts: Government of Zaire, Belgium, Canada, France, South Africa, United Kingdom, United States of America and the WHO, Fonds Medical de Coordination (FOMECO), Fonds Medical Tropical (FOMETRO), Mission medicale Francaise, The US Agency for International Development. Nongovernmental agencies: Oxfam, The Baptists Mission Hospitals, Protestant Mission Aviation Fellowship, Catholic Church (Belgium) | 1976 | Contacts quarantined for 21 days  14†  NR | 'Barrier precautions': cotton gowns and cotton masks. Later replaced with disposable gowns and masks, but gowns and plastic overshoes were re-used when supplies were running low.  ‘Extended barrier precautions' later instated. Following were added:  - Single use items only.  - Balaclava type helmets covering entire head with opening for face.  -Full face respirators (airtight isolation of the face- filter 99·98% effective against 0.3 micron - flow rate of 85L/min) or goggles and disposable surgical mask  Doffing procedure described (Supplement 3) | **Virus transmission -** No secondary transmission. 25/37 total contacts received serological testing (IFA) and all tested negative (proportion of HCWs tested unclear). |

†HCW may include personnel that did not provide direct patient care.

Abbreviations: FOMECO= Fonds Medical de Coordination; FOMETRO= Fonds Medical Tropical; HCW=healthcare worker; IFA=indirect fluorescent antibody; NR=not reported; PPE=personal protective equipment; WHO=World Health Organization

**References**

1. Ebola haemorrhagic fever in Zaire, 1976. Bull World Health Organ 1978; 56(2):271-293.
