# Supplementary material for: Effectiveness of Personal Protective Equipment for Healthcare Workers Caring for Patients with Filovirus Disease: A Rapid Review
Source: PLoS One. 2015 Oct 9;10(10):e0140290. doi: 10.1371/journal.pone.0140290 (PMC4599797; doi:10.1371/journal.pone.0140290)
Supplement: S10 Table — (DOCX) [file pone.0140290.s014.docx]

**S10 Table. Study characteristics of non-comparative studies of healthcare workers wearing gloves, masks, gowns, goggles, respirators, and caps**

| **Study (year of publication)**  **Location**  **Setting**  **Sources of support** | **Year of outbreak** | **Surveillance details**  **Number of participants;**  **Type of HCWs** | **PPE protocol**  **Protocol violations (if reported)** | **Outcomes and results** |
| --- | --- | --- | --- | --- |
| **Ebola Virus Disease** | | | | |
| International Study Team, (1978) [1]  Maridi, Sudan    Hospital, Isolation ward;  Participation of several organizations in outbreak efforts:  Sudanese Ministry of Health, WHO, Federal Embassies of Federal Republic of German and the United States of America, UNESCO, UNDP, ACROSS AND ACORD, CDC. | 1976 | Unclear  230†  Medical assistants, student nurses, workers obtaining samples from isolated patients | Early October: "Strict barrier nursing"  Mid- October: "Strict barrier nursing plus disposable isolation equipment"  Shortly before arrival of WHO on 29-Oct-1976 (after isolation ward erected and likely after large # of HCWs infected): disposable gowns, gloves, masks, caps, full face biological respirators, goggles, and naso-oral respirators.  Workers who visited the isolation ward to extract samples for virological and serological studies: Protective clothing and biological respirators | **Virus transmission –** Unclear. Strict barrier protocol followed with enhanced PPE. Authors provide the number of cases but unclear how many occurred before enhanced PPE. |

†HCW may include personnel that did not provide direct patient care.

Abbreviations: ACORD=Agency for Cooperation and Research Development; CDC=Centers for Disease Control and Prevention; HCW=healthcare worker; NR=not reported; PPE=personal protective equipment; UNDP=United Nations Development Programme; UNESCO=United Nations Educational, Scientific, and Cultural Organization;WHO=World Health Organization

**References**

1. Ebola haemorrhagic fever in Sudan, 1976. Report of a WHO/International Study Team. Bull World Health Organ 1978; 56(2):247-270.
