# Supplementary material for: Effectiveness of Personal Protective Equipment for Healthcare Workers Caring for Patients with Filovirus Disease: A Rapid Review
Source: PLoS One. 2015 Oct 9;10(10):e0140290. doi: 10.1371/journal.pone.0140290 (PMC4599797; doi:10.1371/journal.pone.0140290)
Supplement: S11 Table — (DOCX) [file pone.0140290.s015.docx]

**S11 Table. Study characteristics of non-comparative studies of healthcare workers wearing gloves and barrier techniques (possibly including respirators)**

| **Study (year of publication)**  **Location**  **Setting**  **Sources of support** | **Year of outbreak** | **Surveillance details**  **Number of participants**  **Type of HCWs** | **PPE protocol**  **Protocol violations (if reported)** | **Outcomes and results** |
| --- | --- | --- | --- | --- |
| **Lassa fever** | | | | |
| Haas, WH (2003) [1]  Germany  Local hospital and later transferred to specialized hospital;  Financial support from the Field Epidemiology Training Porgramme, Budesministerium fur Gesundheit, European Programme for Interventions, European Commission and Bundesamt fur ehrtechnik und Baschaffung | 2000 | Contacts monitored and serologic testing conducted 3 weeks after incubation period  Unclear (66† hospital contacts in total; PPE known for casual contacts only but sample size for this subgroup unclear)  NR | Not clearly reported. Casual contacts included those who used gloves and barrier techniques (possibly including protective respirators) during patient care/handling of specimens. PPE worn by high risk contacts or those reporting close physical contact NR | **Virus transmission -** No symptomatic secondary infections among all HCWs. All HCWs reporting use of gloves and barrier techniques (i.e. 'casual contacts') had negative serological test results.  One physician reporting close physical contact tested positive for Lassa virus antibodies (PPE worn NR) |

†HCW may include personnel that did not provide direct patient care.

Abbreviations: HCW=healthcare worker; NR=not reported; PPE=personal protective equipment

**References**

1. Haas WH, Breuer T, Pfaff G et al. Imported Lassa fever in Germany: surveillance and management of contact persons. Clin Infect Dis 2003; 36(10):1254-1258.
