# Supplementary material for: Effectiveness of Personal Protective Equipment for Healthcare Workers Caring for Patients with Filovirus Disease: A Rapid Review
Source: PLoS One. 2015 Oct 9;10(10):e0140290. doi: 10.1371/journal.pone.0140290 (PMC4599797; doi:10.1371/journal.pone.0140290)
Supplement: S12 Table — (DOCX) [file pone.0140290.s016.docx]

**S12 Table. Study characteristics of non-comparative studies of healthcare workers wearing gowns and other equipment (not specified)**

| **Study (year of publication)**  **Location**  **Setting**  **Sources of support** | **Year of outbreak** | **Surveillance details**  **Number of participants**  **Type of HCWs** | **PPE protocol**  **Protocol violations (if reported)** | **Outcomes and results** |
| --- | --- | --- | --- | --- |
| **Lassa fever** | | | | |
| Best, EWR. (1976) [1]  Etobicoke, Canada  Hospital (medical/surgical ward and later isolated)  NR | 1976 | Daily temperature reading and throat swabs; white blood cell counts also taked  >170†  Physician and other hospital staff | Upon admission: Unclear  Patient was isolated 6 days after admission: 'strict isolation procedures' were implemented (e.g. single gowning, etc.).    'High security isolation techniques' were adopted between 6-9 days after admission- (e.g. double gowning) | **Virus transmission –** Appears no secondary transmission occurred (not explicitly stated in report). 22 hospital staff placed in isolation as precaution later discharged. |

†HCW may include personnel that did not provide direct patient care.

Abbreviations: HCW=healthcare worker; NR=not reported; PPE=personal protective equipment

**References**

1. Best EW. The Lassa fever episode, Metro Toronto, August, 1976. [French]. Can J Public Health 369; Revue Canadienne de Sante Publique. 67(5):361-366.
