# Supplementary material for: Effectiveness of Personal Protective Equipment for Healthcare Workers Caring for Patients with Filovirus Disease: A Rapid Review
Source: PLoS One. 2015 Oct 9;10(10):e0140290. doi: 10.1371/journal.pone.0140290 (PMC4599797; doi:10.1371/journal.pone.0140290)
Supplement: S13 Table — (DOCX) [file pone.0140290.s017.docx]

**S13 Table. Study characteristics of non-comparative studies of healthcare workers wearing protective clothing and respirators**

| **Study (year of publication)**  **Location**  **Setting**  **Sources of support** | **Year of outbreak** | **Surveillance details**  **Number of participants**  **Type of HCWs** | **PPE protocol**  **Protocol violations (if reported)** | **Outcomes and results** |
| --- | --- | --- | --- | --- |
| **Ebola Virus Disease** | | | | |
| Richards, GA. (2000) [1]  Johannesburg, South Africa  Tertiary care hospital (ICU used for treatment of VHF cases)  NR | 1996 | Ccontacts ‘placed under observation’ – not further described  Unclear (300 contacts in total; proportion of HCWs NR)  NR | Protective clothing (not further defined) plus HEPA-filtered respirators | **Virus transmission –** No transmission (method of confirmation unclear) |

†HCW may include personnel that did not provide direct patient care.

Abbreviations: HCW=healthcare worker; HEPA=high-efficiency particulate air; ICU=intensive care unit; NR=not reported; PPE=personal protective equipment; VHF=viral hemorrhagic fever

**References**

1. Richards GA, Murphy S, Jobson R et al. Unexpected Ebola virus in a tertiary setting: clinical and epidemiologic aspects. Crit Care Med 2000; 28(1):240-244.
