# Supplementary material for: Effectiveness of Personal Protective Equipment for Healthcare Workers Caring for Patients with Filovirus Disease: A Rapid Review
Source: PLoS One. 2015 Oct 9;10(10):e0140290. doi: 10.1371/journal.pone.0140290 (PMC4599797; doi:10.1371/journal.pone.0140290)
Supplement: S14 Table — (DOCX) [file pone.0140290.s018.docx]

**S14 Table. Study characteristics of non-comparative studies of healthcare workers wearing PPE according to the WHO protocol**

| **Study (year of publication)**  **Location**  **Setting**  **Sources of support** | **Year of outbreak** | **Surveillance details**  **Number of participants**  **Type of HCWs** | **PPE protocol**  **Protocol violations (if reported)** | **Outcomes and results** |
| --- | --- | --- | --- | --- |
| **Ebola Virus Disease** | | | | |
| Borchert, M. (2011) [1]  Masindi District, Uganda  Hospital, Ebola ward  Participation of several organizations: CDC, EpiCentre, Health Canada, Institute of Tropical Medicine, MSF, Ministry of Health, Republic of Ghana; WHO, African Region and Headquarters | 2000 | Followed for 21 days or until suspect case cleared  NR  NR | Donning and doffing according to WHO protocol (not further described)  Disinfection of equipment and gloves with 0·05% chlorine solution between patient contacts | **Virus transmission -** Five HCWs infected after implementation of protocol (likely attributed to protocol violations). Confirmation using ELISA or PCR. |
| **Marburg Virus Disease** | | | | |
| Gear, JSS. (1975) [2]  Johannesburg, South Africa  Hospital | 1975 | Contacts isolated and monitored  35  Doctors, Nurses | Admission of index case- 'Barrier nursing' was implemented 2 hours after admission - gowns, gloves, and masks were prescribed.  ‘Strict barrier nursing' was implemented as recommended by the WHO after secondary case identified  PPE use of infected nurse varied - During intubation/resuscitation of index case, wore PPE. Consoled the first secondary case without use of gloves when handling facial tissue. Eight days later, wore PPE and goggles. | **Virus transmission -** Secondary transmission of disease to a nurse. Convalescent blood sample tested positive for antibodies against Marburg virus. Samples taken from her eye 2 months later was isolated and Marburg virus was detected. |

†HCW may include personnel that did not provide direct patient care.

Abbreviations: CDC=Centers for Disease Control and Prevention; ELISA=enzyme-linked immunosorbent assay; HCW=healthcare worker; MSF=Médecins Sans Frontières; NR=not reported; PCR=polymerase chain reaction; PPE=personal protective equipment; WHO=World Health Organization

**References**

1. Borchert M, Mutyaba I, Van Kerkhove MD et al. Ebola haemorrhagic fever outbreak in Masindi District, Uganda: outbreak description and lessons learned. BMC Infect Dis 2011; 11:357.
2. Gear JS, Cassel GA, Gear AJ, Trappler B, Clausen L, Meyers AM, et al. Outbreake of Marburg virus disease in Johannesburg. Br Med J 1975 Nov 29;4(5995):489-93. [PMID: 811315]
