# Supplementary material for: Effectiveness of Personal Protective Equipment for Healthcare Workers Caring for Patients with Filovirus Disease: A Rapid Review
Source: PLoS One. 2015 Oct 9;10(10):e0140290. doi: 10.1371/journal.pone.0140290 (PMC4599797; doi:10.1371/journal.pone.0140290)
Supplement: S15 Table — (DOCX) [file pone.0140290.s019.docx]

**S15 Table. Study characteristics of non-comparative studies of healthcare workers wearing PPE according to the Advisory Committee on Dangerous Pathogens guidelines**

| **Study (year of publication)**  **Location**  **Setting**  **Sources of support** | **Year of outbreak** | **Surveillance details**  **Number of participants**  **Type of HCWs** | **PPE protocol**  **Protocol violations (if reported)** | **Outcomes and results** |
| --- | --- | --- | --- | --- |
| **Crimean-Congo Hemorrhagic Fever** | | | | |
| Barr, DA. (2013) [1]  Glasgow, United Kingdom  Centre for Infectious diseases (isolated in negative-pressure room)  NR | 2012 | Surveillance conducted – not further described  NR  NR | Upon admission - infection control measures as per ACDP guidelines were used (protocol not further described) | **Virus transmission –** No secondary transmission (method of confirmation NR) |

†HCW may include personnel that did not provide direct patient care.

Abbreviations: ACDP=Advisory Committee on Dangerous Pathogens; HCW=healthcare worker; NR=not reported; PPE=personal protective equipment

**References**

1. Barr DA, Aitken C, Bell DJ et al. First confirmed case of Crimean-Congo haemorrhagic fever in the UK. The Lancet 2013; 382(9902):1458.
