# Supplementary material for: Effectiveness of Personal Protective Equipment for Healthcare Workers Caring for Patients with Filovirus Disease: A Rapid Review
Source: PLoS One. 2015 Oct 9;10(10):e0140290. doi: 10.1371/journal.pone.0140290 (PMC4599797; doi:10.1371/journal.pone.0140290)
Supplement: S16 Table — (DOCX) [file pone.0140290.s020.docx]

**S16 Table. Study characteristics of non-comparative studies of healthcare workers wearing PPE according to ‘standard precautions’ including masks**

| **Study (year of publication)**  **Location**  **Setting**  **Sources of support** | **Year of outbreak** | **Surveillance details**  **Number of participants**  **Type of HCWs** | **PPE protocol**  **Protocol violations (if reported)** | **Outcomes and results** |
| --- | --- | --- | --- | --- |
| **Crimean-Congo Hemorrhagic Fever** | | | | |
| Tarantola, A. (2006) [1]  (1) Dakar, Senegal  Hospital (double room)  (2) Repatriated to Rennes, France    University Hospital (single room in ICU, not negative pressure then transferred to single room in infectious disease department)  NR | 2004 | Followed up for 10 days; self-reporting of symptoms and daily follow-up by clinician  90†  Nurses, physicians, medical studies, nurses’ aides, dentist, others (not described) | Only 'standard precautions' used in Senegal  In ICU, strict adherence to 'standard precautions' including use of surgical masks. | **Virus transmission –** No secondary transmission of disease; no contact developed clinical symptoms |

†HCW may include personnel that did not provide direct patient care.

Abbreviations: HCW=healthcare worker; ICU=intensive care unit; NR=not reported; PPE=personal protective equipment

**References**

1. Tarantola A, Nabeth P, Tattevin P et al. Lookback exercise with imported crimean-congo hemorrhagic fever, Senegal and France. Emerg Infect Dis 2006; 12(9):1424-1426.
